# Synthesis and Functionalization of Challenging *meso*-Substituted Aryl Bis-pocket Porphyrins Accessed via Suzuki–Miyaura Cross-Coupling

**DOI:** 10.1021/acs.joc.2c01538

**Published:** 2022-08-17

**Authors:** Daniel G. Droege, A. Leila Parker, Griffin M. Milligan, Robert Jenkins, Timothy C. Johnstone

**Affiliations:** Department of Chemistry and Biochemistry, University of California, Santa Cruz, California 95064, United States

## Abstract

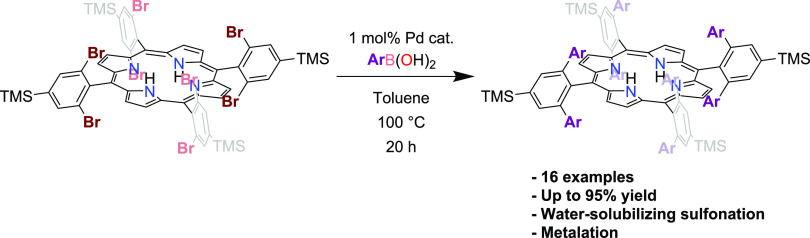

Herein we report an investigation into the synthesis, metalation, and functionalization of bis-pocket porphyrins using the Suzuki–Miyaura cross-coupling reaction. Steric limitations to accessing bis-pocket porphyrins were overcome by using this Pd-catalyzed C–C-bond-forming strategy to introduce steric bulk *after* macrocyclization: 2,6-dibromo-4-trimethylsilybenzaldehyde was condensed with pyrrole, and a variety of boronic acids were coupled to the resulting porphyrin in up to 95% yield. Furthermore, we show that these porphyrins can be metalated with a variety of metals and sulfonated to create water-soluble bis-pocket porphyrins.

## Introduction

The porphyrin macrocycle holds a privileged position in biology and chemistry. Porphyrin-based cofactors play essential roles in O_2_ transport, oxidative metabolism, and photosynthetic light-harvesting (although chlorophylls *a*, *b*, *d*, and *f* feature a chlorin core, chlorophylls *c_1_* and *c_2_* feature a porphyrin core). The earliest systematic investigation of porphyrin compounds was centered on their isolation from organismal sources,^1^ including the work by Hoppe-Seyler in the late 19th century on porphyrins derived from blood and chlorophyll.^2,3^ Subsequent efforts turned to chemical synthesis, and Fischer and Halbig, using a bis(carboxypyrro)methane, reported the first porphyrin synthesis in 1926.^4^ The following decade, Fischer and Gleim reported that the parent hydride, porphine, could be obtained by refluxing pyrrole-2-carboxaldehyde in formic acid.^5^ At this same time, Rothemund reported the now-familiar strategy of condensing pyrrole and an aldehyde to afford either porphine, if formaldehyde was used, or *meso*-substituted porphyrins.^6,7^ Refinement of synthetic strategies to access porphyrins continued, and in 1967, Adler et al. described the ease with which many *meso*-substituted porphyrins can be synthesized and purified when the condensation of pyrrole and aldehyde was performed over a short period of time in refluxing propionic acid.^8^ Lindsey and Wagner subsequently developed a two-step procedure involving BF_3_-catalyzed macrocyclization of pyrrole and aldehyde to form a porphyrinogen followed by oxidation with 2,3-dichloro-5,6-dicyano-l,4-benzoquinone (DDQ) to give a porphyrin.^9^ This strategy proved particularly adept at preparing sterically hindered *meso*-substituted porphyrins, including tetramesitylporphyrin.

**Figure 1:**
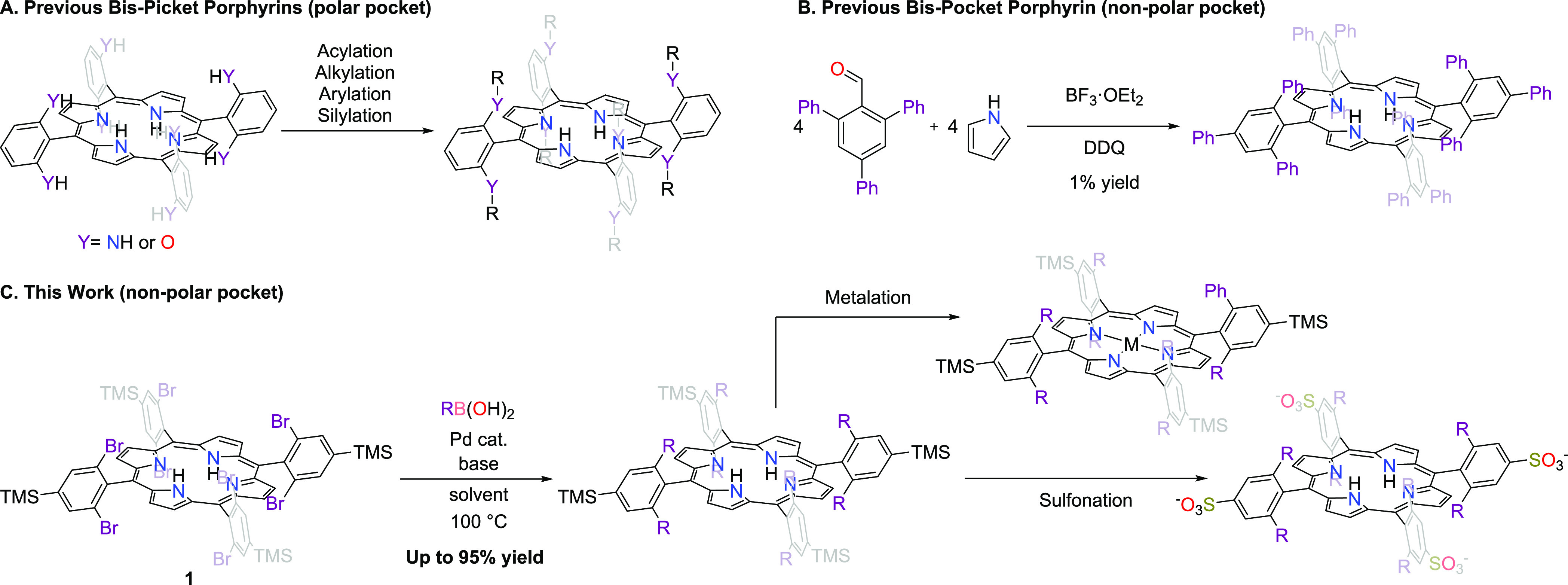
Examples of bis-pocket porphyrin syntheses.

Although motivated originally by a desire to understand naturally occurring porphyrin compounds, subsequent interest in preparing small-molecule models of metalloprotein active sites, catalysts that facilitate important chemical transformations, and synthetic light-harvesting systems drove chemists to develop sophisticated strategies to target increasingly complex porphyrin scaffolds.^10,11^ Concerning metalloporphyrin derivatives, a particular focus has been placed on controlling the environment around the metal ion above and below the plane of the porphyrin. The ″picket-fence″ porphyrins synthesized by Collman and coworkers were prepared by coupling bulky carboxylic acids to amino groups at the *ortho* positions of *meso* phenyl substituents.^12^ Related strategies have been used to prepare capped, strapped, basket, bis-picket, and basket-handle porphyrins.^13^ In addition to amide-bond-forming reactions, various groups have used esterification, etherification, and silylation reactions to build up the environment around the metal center following macrocyclization (Figure 1).

Although these strategies can introduce significant hydrophobic bulk above and below the plane of the porphyrin, they nevertheless install polar functional groups near the metal center. Suslick and Fox succeeded in preparing a ″bis-pocket″ porphyrin featuring fully hydrophobic pockets by coupling pyrrole and 2,4,6-triphenylbenzaldehyde using modified Adler conditions, but it could only be prepared in 1% yield.^14^ They used the Fe complex of this ligand as a model for heme-based proteins and the Mn complex as an alkane hydroxylation catalyst.^14−18^ The hydrophobic bis-pocket motif was more recently used by Che and coworkers to trap and characterize reactive metal-carbene complexes and carry out C–H activation.^19,20^ We recently targeted a bulky Fe(II) porphyrin complex with hydrophobic pockets for exploration as an antidote for carbon monoxide poisoning.^21^ Faced by the same low yields encountered previously when coupling pyrrole and terphenylaldehyde derivatives using Adler or Lindsey conditions, we turned to a strategy in which we installed the steric bulk *after* macrocyclization. To do so without introducing any polarity into the pocket, we used Pd-catalyzed C–C-bond-forming reactions to directly attach phenyl rings to the *ortho* positions of *meso* phenyl groups. We note that, as our work was being published, a similar approach was described by Higuchi and coworkers for the preparation of bis-pocket Ru porphyrin complexes for exploration as alkane oxidation catalysts.^22^ In our original publication, we described the coupling of PhB(OH)_2_ to 5,10,15,20-tetrakis(2,6-dibromo-4-(trimethylsilyl)phenyl)porphyrin (**1**) to give 5,10,15,20-tetrakis(2,6-diphenyl-4-(trimethylsilyl)phenyl)porphyrin (**2a**). Here, we describe an optimization of this coupling reaction, achieved by varying the catalyst, catalyst loading, base, stoichiometry, solvent, and temperature. We demonstrate that the method allows a variety of different groups varying in sterics, electronics, and functional group presentation to be coupled to the porphyrin framework. The TMS groups on the porphyrin derivatives provide excellent organic solubility even when large aromatic groups are installed. We also describe an optimization of the sulfonation reaction reported in our initial paper. In this reaction, the TMS groups are exchanged for SO_3_^–^ groups that confer water solubility on these bulky porphyrins. Finally, we demonstrate that although the bulky substituents can inhibit the insertion of some metals into these porphyrins, refluxing a metal halide, 2,6-lutidine, and the free-base ligand in 1,2,4-trichlorobenzene (1,2,4-TCB) permits facile and rapid metalation. We anticipate that these reactions will be useful in the preparation of porphyrin compounds intended for a range of fundamental and applied studies.

## Results and Discussion

### Reaction Optimization

In our prior work, we described the synthesis of **2a**, which we accessed via Pd-catalyzed cross-coupling of **1** and PhB(OH)_2_.^21^ This Suzuki–Miyaura reaction was performed over 16 h in 20:1 1,4-dioxane/water using a threefold excess of PhB(OH)_2_ (per Ar–Br bond), Cs_2_CO_3_ as a base, and 12.5 mol % (dppf)PdCl_2_ (per Ar–Br bond). We used these conditions as the starting point for the presently described optimization of the coupling of **1** with arylboronic acids. We were particularly interested in optimizing the yield while minimizing the amount of catalyst used; the initial 12.5 mol % (per Ar–Br bond) ensured that the product was obtained in our previous work^21^ but is assuredly in excess of the amount needed to achieve this goal.

We began by iteratively decreasing the amount of Pd catalyst (Table 1, entries 1–4). The yield of the reaction remained unchanged with a catalyst loading as low as 1 mol % (per Ar–Br bond). The loading could be further reduced to 0.5 mol %, but a longer reaction time of 20 h was needed to ensure that the reaction proceeded to completion (Table 1, entry 5). A loading of 0.1 mol % resulted in negligible formation of the product even at the longer reaction time (Table 1, entry 6). Subsequent steps in the optimization were performed using 1 mol % catalyst.

**Table 1:**
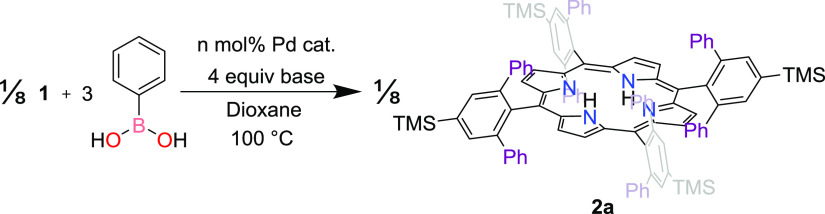
Optimization of Catalyst Loading for Coupling^a^

entry	catalyst	mol % cat	yield	time (h)
1	(dppf)PdCl_2_	12.5	76%	16
2	(dppf)PdCl_2_	5	77%	16
3	(dppf)PdCl_2_	1	73%	16
4	(dppf)PdCl_2_	0.5	60%	16
5	(dppf)PdCl_2_	0.5	72%	20
6	(dppf)PdCl_2_	0.1	0%	20
7	none	0	0%	20

a The mol % of catalyst, equiv PhB(OH)_2_, and equiv base are provided with respect to Ar–Br bonds.

We next screened a panel of Pd catalysts with a focus on complexes known to facilitate similar coupling reactions, especially those used to catalyze the coupling of sterically hindered substituents (Table 2).^23^ We observed that Pd(PPh_3_)_4_ performed comparably to (dppf)PdCl_2_ (Table 2, entry 1). Pd catalysts with bidentate ligands, such as Xantphos and rac-BINAP, afforded greater yields than catalysts with the monodentate ligands SPhos and DavePhos (Table 2, entries 2–5). The precatalyst alone afforded no product (Table 2, entry 6). Under our initial set of reaction conditions, (dppf)PdCl_2_ and Pd(PPh_3_)_4_ were the only catalysts we tested that allowed the reaction to reach completion; all others gave mixtures of the desired product and various partially functionalized intermediates with fewer than eight of the aryl bromides having reacted. These partially substituted intermediates can be separated from the desired product by silica gel column chromatography, but even with extensive optimization, the resolution of the intermediates and product was poor. To quantitatively evaluate the different catalysts (and other reaction conditions, *vide infra*), we collected all partially substituted intermediates along with the product and determined the fraction of that porphyrinic material corresponding to the desired product using NMR spectroscopy. Although the aromatic ^1^H NMR resonances of these species overlap, the N-H signals, characteristically shifted upfield (δ < −2 ppm) because of their position within the center of the strong diamagnetic ring current of the porphyrin macrocycle, are well separated. Indeed, the reactions could be monitored readily by observing the progressive growth and disappearance of the N-H signal(s) of each intermediate. The yields reported below were calculated by multiplying the mass of the total isolated porphyrinic material by the quotient of the integral of the N-H resonance of the desired product and the integral of all N-H resonances in the isolated material. Although Pd(PPh_3_)_4_ afforded a reaction yield comparable to that of (dppf)PdCl_2_, we chose to continue our optimization with (dppf)PdCl_2_ due to its relatively low cost, ease of use, and benchtop stability.

**Table 2:**
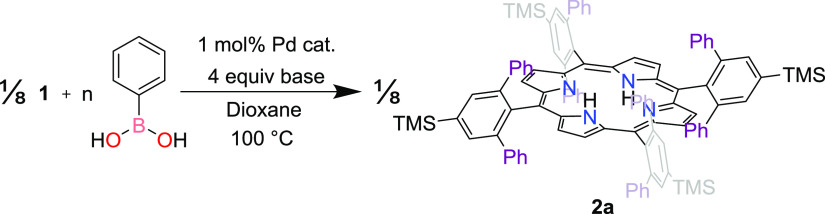
Optimization of Coupling with PhB(OH)_2_^a^

entry	catalyst	equiv PhB(OH)_2_	base	solvent	yield
1	Pd(PPh_3_)_4_	3	Cs_2_CO_3_	dioxane	79%
2	Pd_2_(DBA)_3_/Xantphos^b^	3	Cs_2_CO_3_	dioxane	57%
3	Pd_2_(DBA)_3_/rac-BINAP^b^	3	Cs_2_CO_3_	dioxane	70%
4	Pd_2_(DBA)_3_/Sphos^b^	3	Cs_2_CO_3_	dioxane	8%
5	Pd_2_(DBA)_3_/DavePhos^b^	3	Cs_2_CO_3_	dioxane	3%
6	Pd_2_(DBA)_3_^c^	3	Cs_2_CO_3_	dioxane	0%
7	(dppf)PdCl_2_	3	KOH	dioxane	0%
8	(dppf)PdCl_2_	3	KOAc	dioxane	51%
9	(dppf)PdCl_2_	3	K_3_PO_4_	dioxane	57%
10	(dppf)PdCl_2_	3	K_2_CO_3_	dioxane	36%
11	(dppf)PdCl_2_	3	Cs_2_CO_3_	DMF	0%
12	(dppf)PdCl_2_	3	Cs_2_CO_3_	diglyme	70%
13	(dppf)PdCl_2_	3	Cs_2_CO_3_	toluene	89%
14	(dppf)PdCl_2_	4	Cs_2_CO_3_	toluene	93%
15	(dppf)PdCl_2_	2	Cs_2_CO_3_	toluene	6%
16	(dppf)PdCl_2_	3	Cs_2_CO_3_^d^	toluene	77%

a The mol % of catalyst, equiv PhB(OH)_2_, and equiv base are provided with respect to Ar–Br bonds.

b 1 mol % Pd_2_(DBA)_3_ (per Pd atom) and 2 mol % of the specified ligand were precomplexed prior to the start of the reaction.

c No ligand added.

d 3 equiv.

We next explored the use of different bases that have proven successful in previous Suzuki–Miyaura couplings.^24^ KOH, which has a significantly lower p*K*_b_ than Cs_2_CO_3_, formed no product (Table 2, entry 7). Increasing the p*K*_b_ (KOAc) was detrimental to the yield, and the reaction did not reach completion within 20 h (Table 2, entry 8). Reactions with K_3_PO_4_ and K_2_CO_3_ also failed to reach completion within 20 h and featured a consequent drop in yield (Table 2, entries 9 and 10).

Keeping Cs_2_CO_3_ as the base, we next investigated the influence of changing the solvent (Table 2, entries 11–13). Solvent choices were limited due the solubility of the starting porphyrin. For example, performing the reaction in DMF afforded no product, which we attribute to the poor solubility of the starting porphyrin in this solvent, even at elevated temperatures. Diglyme gave a comparable yield to dioxane (Table 2, entry 12). Switching the solvent to toluene, in which the starting porphyrin is more soluble, increased the yield to 89% (Table 2, entry 13). Including an additional equivalent of PhB(OH)_2_ in the reaction (for a total of 4 equiv) increased the yield slightly to 93% (Table 2, entry 14). It should be noted that this value reflects an isolated yield of purified, recrystallized product. Finally, we confirmed that decreasing the amount of either PhB(OH)_2_ or Cs_2_CO_3_ was detrimental to the reaction yield (Table 2, entries 15 and 16).

### Reaction Scope

**Figure 2:**
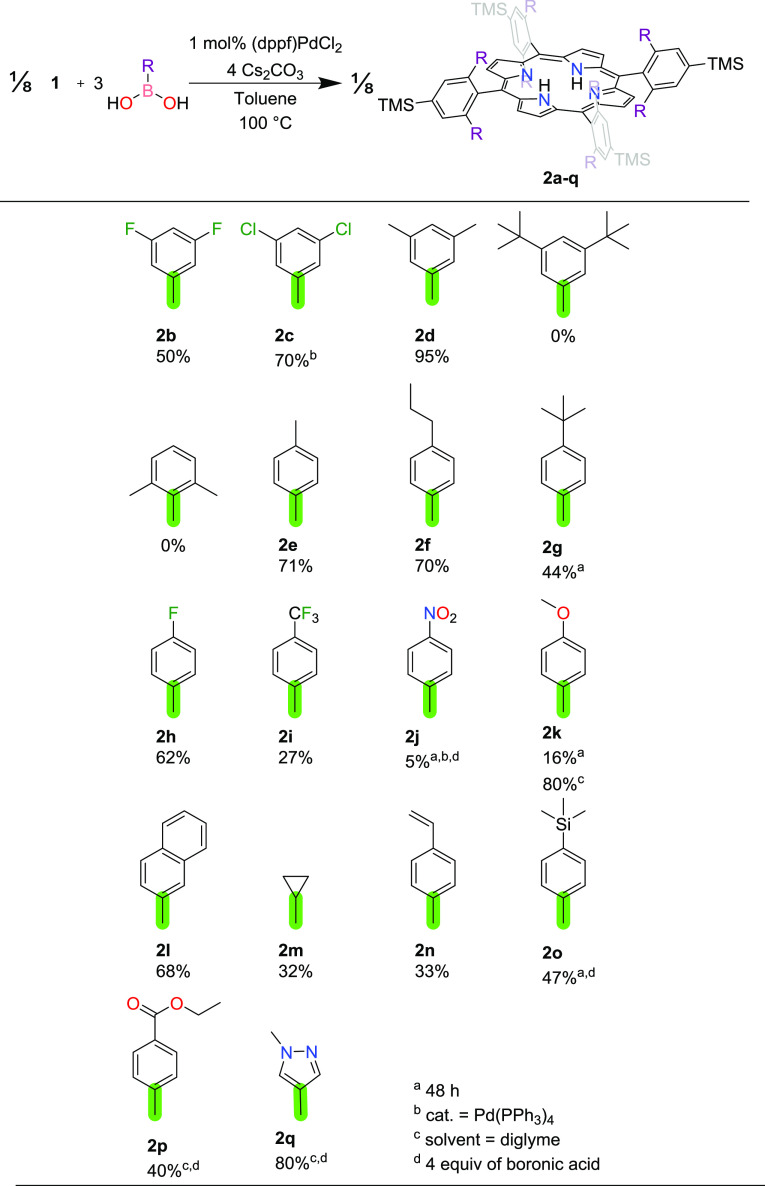
Exploration of the scope of groups that can be coupled to the porphyrin framework according to the depicted reaction. Yields are isolated yields.

With a set of optimized reaction conditions for coupling **1** to PhB(OH)_2_ in hand, we sought to explore the versatility of groups that could be installed on the porphyrin framework in this way (Figure 2). Because of the inherent congestion of the bis-pocket motif, we were particularly interested in exploring the limitations imposed by the size of the substituent to be installed. We began by increasing the bulk at the 3 and 5 positions of phenylboronic acid. Analysis of our previously reported crystal structure of **2a** highlights that the canting of the phenyl rings poised above and below the plane of the porphyrin causes their 3 and 5 positions to point at each other.^21^ The 3,5-difluoro, -dichloro, and -dimethyl derivatives of phenylboronic acid were tolerated in the coupling, but further increase in size to 3,5-di-*tert*-butylphenylboronic acid afforded no product. Unsurprisingly, introduction of steric bulk at the 2 and 6 positions was detrimental, and 2,6-dimethylphenylboronic acid afforded no product. The 4 position of phenylboronic acid could tolerate a range of larger substituents; with methyl, *n*-propyl, or *tert*-butyl groups at this position, the coupling could be successfully performed. In the case of the 4-*tert*-butylphenylboronic acid, the yield was attenuated and the reaction time was increased to 48 h.

We also studied the influence of the electronic properties of the substituents on the coupling of phenylboronic acid derivatives. Using our initially optimized conditions, aromatic rings functionalized with electron-withdrawing halogen substituents, such as 3,5-difluorophenyl and 4-fluorophenyl, could be installed with moderately high yields. The 4-fluorophenyl-substituted product, **2h**, was scaled up to 1 mmol of coupled arylboronic acid, and the product could successfully be obtained in 71% isolated yield (154 mg). We noted a trend whereby an increase in the number/strength of electron-withdrawing groups systematically decreased the yield. For instance, 4-fluorophenyl-, 4-trifluoromethylphenyl-, and 4-nitrophenyl-substituted products were obtained in systematically decreased yield, consistent with their respective σ_p_ values of 0.06, 0.54, and 0.78. Indeed, to obtain even a 5% yield of the 4-nitrophenyl-coupled product, we used an extended reaction time of 48 h, included an extra equivalent of arylboronic acid, and changed the catalyst to Pd(PPh_3_)_4_. Installation of the electron-donating 4-methoxyphenyl group was similarly inhibited under the standard reaction conditions. Although the electronics of the substituents have a notable impact on the yield using the general reaction conditions from Figure 2, we note that the products are still achievable and, as described below, simple changes to the method can allow for the facile optimization of the yield of any particular product. The ability to modulate the electronics of this platform will be useful in many of the applications of porphyrin compounds, not the least of which is the formation of metalloporphyrin complexes, whose properties and reactivity can be a sensitive function of the electron-donating capacity of the porphyrin ligand. Without changing the standard reaction conditions, we were also able to successfully couple **1** to either 2-naphthylboronic acid or cyclopropylboronic acid. The latter example demonstrated that the strategy is not restricted to forming sp^2^–sp^2^ C–C bonds.

Given the steric and electronic trends established thus far, we were initially surprised by the poor yield for the product in which eight 3,5-dichlorophenyl groups were installed; better yields were obtained with groups that were both larger and smaller as well as with groups that were more or less electron-withdrawing. Although (dppf)PdCl_2_ is generally not employed in the coupling of aryl chlorides and arylboronic acids, we hypothesized that the decrease in yield stemmed from overderivatization.^25^ That is, additional 3,5-dichlorophenyl groups were coupled to 3,5-dichlorophenyl groups that had already been attached to the porphyrin scaffold. Mass spectrometric analysis of the crude reaction confirmed this hypothesis; prominent signals were observed for overcoupling (*m*/*z* = 2172.58, 2285.54, and 2394.58). We reasoned that this undesired reactivity could be avoided by using a catalyst that would not readily undergo oxidative addition with aryl chlorides.^26^ To our benefit, we had already established that Pd(PPh_3_)_4_, which should be even less competent to couple aryl chlorides than (dppf)PdCl_2_, worked well for our desired coupling. Indeed, using 1 mol % Pd(PPh_3_)_4_ as the catalyst afforded the desired product in 70% yield without any further change to the reaction conditions (Figure 2).

Having demonstrated the general electronic and steric tolerance of this strategy for porphyrin functionalization, we wanted to further demonstrate its usefulness in preparing porphyrin precursors suitable for further functionalization. In addition to the 4-nitrophenyl derivative described above, which can be reduced to afford reactive amine units, we were also able to successfully install 4-vinylphenyl groups, which are amenable to further functionalization via alkene metathesis. 4-Trimethylsilylphenyl groups could be installed, although the reaction proceeded more slowly than many of the others. Increasing the reaction time to 48 h and including an extra equivalent of boronic acid allowed the product to be obtained in 47% yield.

We were also interested in the installation of ester groups, which would be able to undergo either transesterification or saponification. Unfortunately, coupling of **1** and 4-ethoxycarbonylphenylboronic acid was unsuccessful using our standard conditions. Simple substitution of the solvent for diglyme and inclusion of an extra equivalent of boronic acid provided the desired product in 40% yield. This example highlights that our porphyrin functionalization strategy maintains one of the well-established benefits of Pd-catalyzed C–C-bond-forming reactions: the modular nature of the synthetic protocol allows for rapid and efficient screening of the solvent, catalyst, base, temperature, and reaction time to allow for ready incorporation of a given group. As a further example of the flexibility of the method, we highlight that our standard reaction conditions did not permit the coupling of **1** and 1-methylpyrazole-4-boronic acid but that this product formed in 80% yield using the same toluene-to-diglyme solvent substitution described above and an extra equivalent of boronic acid, but no further optimization of reaction conditions.

Our aim in this work is to highlight that this strategy is amenable to preparing bis-pocket porphyrins with a variety of different substituents. We optimized our reaction conditions with PhB(OH)_2_ to obtain a general set of conditions that would allow us to demonstrate the feasibility of incorporating these groups. If any particular group is desired, then further optimization of the reaction temperature, time, solvent, and stoichiometry will invariably allow yields greater than those in Figure 2 to be obtained. For example, although the coupling with 4-methoxyphenylboronic acid was successful using our standard reaction conditions, changing the solvent to diglyme increased the yield fivefold (Figure 2).

### Bis-pocket Porphyrin Architecture

The aryl substituents introduced at the 2 and 6 positions of the *meso* phenyl groups create pockets above and below the plane of the porphyrin, giving rise to the ″bis-pocket″ moniker introduced by Suslick and Fox.^14^ By varying the nature and positions of the substituents decorating these aryl rings, the pockets can be sculpted. We have successfully crystallized 13 of the free-base TMS-functionalized porphyrin compounds **2**, in addition to **2a**, the crystal structure of which we previously described.^21^ The structures confirm, in all cases, the connectivity of the desired products. In many cases, the porphyrin resides on an inversion center, but in no case is the planarity of the entire porphyrin core crystallographically required. Nevertheless, the porphyrins exhibit little distortion from planarity, with the greatest deviation (RMSD = 0.091 Å) observed for **2c**, R = 3,5-dichlorophenyl.

Because the crystals are not isostructural, we do not anticipate seeing well-defined relationships between the molecular structure and pocket volume. The shapes and volumes of the pockets are impacted significantly by torsion angles, the shallow potential energy profiles of which allow them to be readily deformed by crystal packing forces. The present structures do, however, reveal the variety of pocket shapes and sizes that can be accessed when the substituents are varied. The structures of a number of the compounds (**2b**, **2e**, **2f**, **2h**, **2k**, **2l**, **2o**) feature a pocket on either side of the plane of the porphyrin, and both pockets contain solvent molecules. The structure of **2i** also features two pockets, one above and one below the plane of the porphyrin, but neither contains a solvent molecule. In the structures of **2c** and **2d**, one pocket contains a solvent molecule, and the other does not. Interestingly, these are two of the most sterically congested porphyrins prepared in this study. We hypothesize that the internal motions that open one pocket sufficiently to accommodate a solvent molecule cause the other pocket to collapse. A similar effect is observed in the structure of **2q**, in which adjacent molecules interlock across an inversion center with an *N*-methylpyrazolyl group of each residing in a pocket of the other. The tilting of the *meso* terphenyl groups to open up the pocket on one face leads to the closing of the pocket on the opposite face.

**Figure 3:**
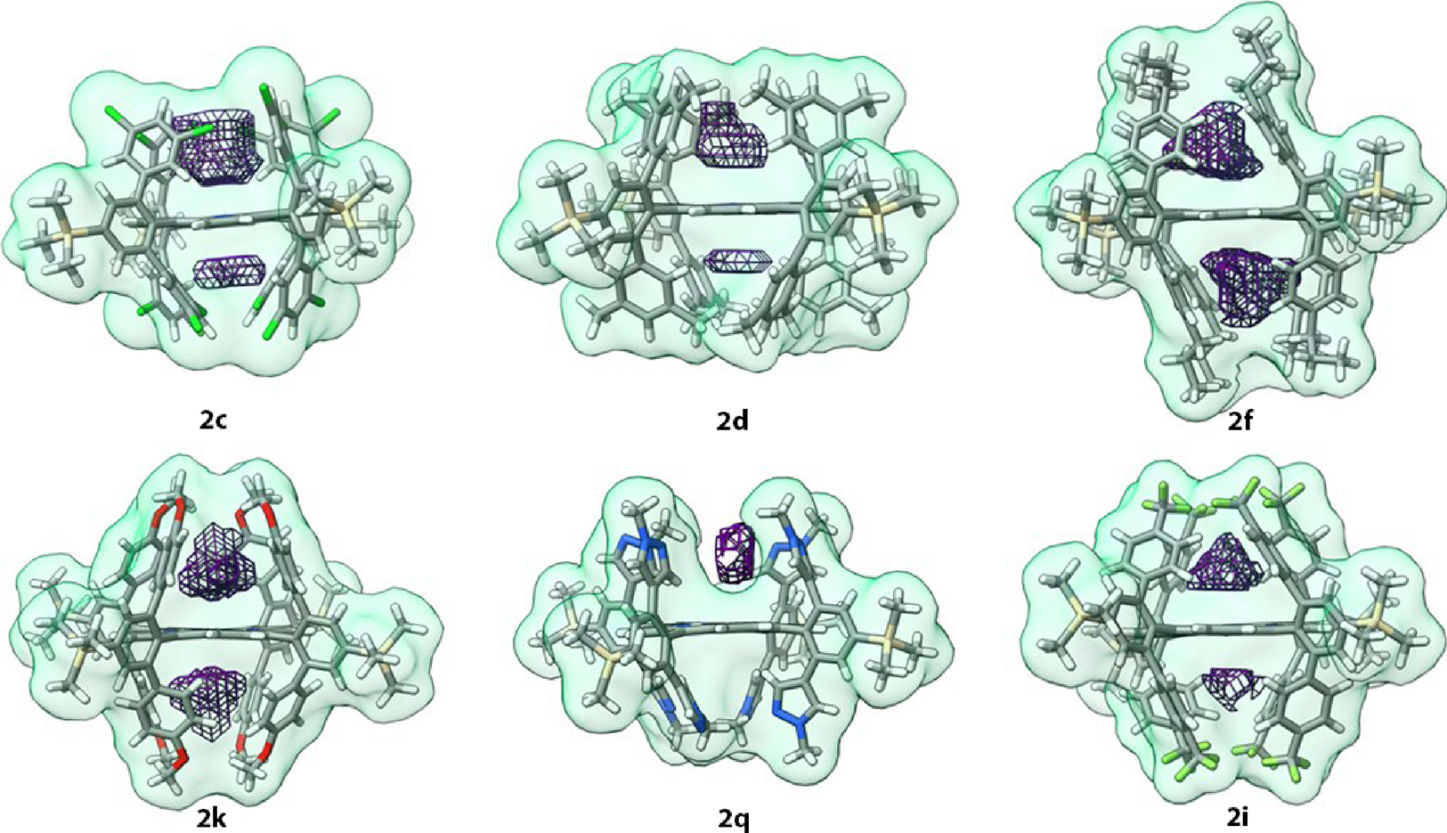
Pockets of **2c**, **2d**, **2f**, **2k**, **2q**, and **2i** as calculated with POVME2 using atomic coordinates from single-crystal X-ray diffraction data. The molecules are shown as sticks with a green surface at the van der Waals distance. The pockets are depicted as purple mesh. Atomic color code: C gray, H white, O red, N blue, Si tan, Cl green, and F light green. Molecular graphics and analyses performed with UCSF ChimeraX.^28^

To quantify these variations in pocket volume, we employed POVME2, a tool developed to measure the volumes of protein pockets (Figure 3 and Table S8).^27^ We note that the absolute values of the volumes obtained from different pocket volume estimation algorithms can vary significantly but that relative values tend to accurately reflect trends in volumes.^27^ Consistent with the analysis above, the volume estimates for **2c**, **2d**, and **2q** reflect the differences in the volumes of the two pockets, whereas **2b**, **2e**, **2f**, **2h**, **2i**, **2k**, **2l**, and **2o** each feature pockets with similar or identical volumes (the latter arising in the case of crystallographic equivalence). The volume estimates also highlight the variation in pocket shape from one molecule to the next. For example, the volumes of the pockets for **2a** and **2l** are approximately equal despite the fact that **2a** features phenyl substituents and **2l** features the taller naphthyl substituents. The increase in pocket height for **2l** is offset by a narrowing of the pocket width.

The torsionally defined pockets present in these crystal structures are undoubtedly influenced by crystal packing forces in many instances, but we reiterate that they highlight the variability in pocket size/shape that is accessible with this scaffold. This diversity is showcased in Figure 3.

### Sulfonation

The TMS groups present in the porphyrin starting material serve a number of functions. In addition to providing additional ^1^H, ^13^C, and ^29^Si NMR spectroscopic handles, they impart increased organic solubility to **1** facilitating the coupling reaction. The enhanced organic solubility extends to the products, which can be helpful for either solution-phase processing of the products or investigation of their solution-phase properties/reactivity. In addition to advantages related to organic solubility, the TMS groups also provide a means of performing regioselective sulfonation.^29^ Sulfonation of these porphyrins can confer upon them greater solubility in polar organic solvents or, in some cases, aqueous solubility. In our previous work, we reported that 5,10,15,20-tetrakis(2,6-diphenyl-4-(trimethylsilyl)phenyl)porphyrinatohydroxoiron(III) could be converted to the corresponding tetrasulfonate salt in 40% yield by treatment with trimethylsilyl chlorosulfonate in refluxing CCl_4_ for 1 h followed by aqueous alkaline workup.^21^ Although we had obtained evidence that this low yield arose from the desilylation of the starting material, the 40% yield was sufficient to obtain the amount of material needed to test the CO-sequestering properties of this metalloporphyrin.

**Figure 4:**
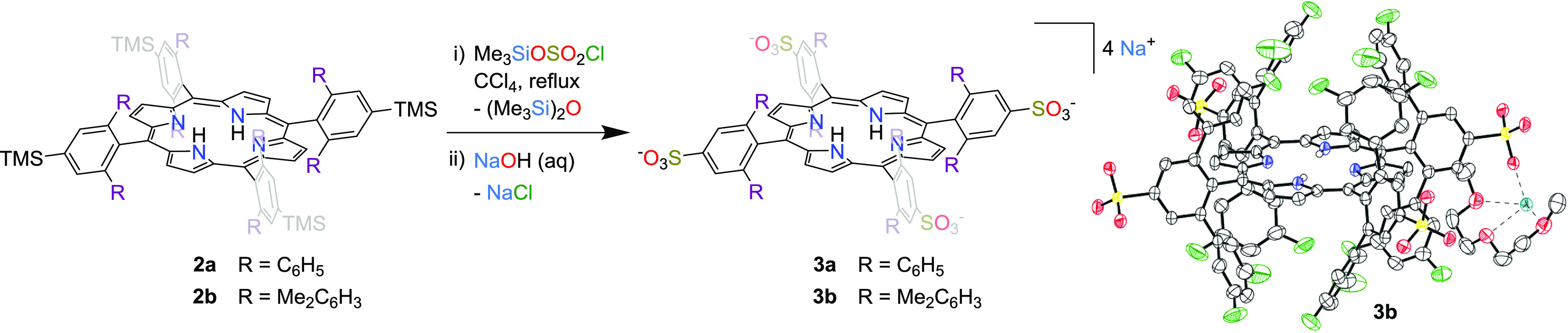
Sulfonation of bulky bis-pocket porphyrins. At right, thermal ellipsoid plot (50% probability level) of **3b** with nonpolar H atoms and three of the four Na^+^–diglyme complexes omitted for clarity. Color code: O red, N blue, F green, Na teal, S, yellow, C gray, and H white spheres of arbitrary radius.

To decrease desilylation, we performed the sulfonation of the free-base **2a** with fewer equivalents of sulfonating agent (1.2 equiv). We were able to obtain the tetrasulfonated product **3a** in 60% yield by performing the reaction at 75 °C for 1 h. We were similarly able to obtain **3b**, the tetrasulfonated derivative of **2b**, in 84% yield (Figure 4). This mild reaction provides a convenient means of drastically altering the solubility of the porphyrin: **3a** and **3b** can be dissolved in water and methanol, whereas the starting molecules **2a** and **2b** are completely insoluble in these solvents. Saturated aqueous solutions of **3a** and **3b** have concentrations of 9.5 and 5.8 mM, respectively. The UV–vis spectra of **3a** and **3b** remain the same as their nonsulfonated counterparts.

Although all of the porphyrin compounds described in this paper generally crystallize well, we encountered a number of difficulties in growing diffraction-quality crystals of the porphyrin sulfonate salts. Crystals grown under a variety of different conditions would exhibit intractable twinning or cracking. We ultimately succeeded in growing diffraction-quality crystals of **3b** by including diglyme in the crystallization (Figure 4). The sodium cation, residing on a general position, is chelated by a molecule of diglyme and otherwise interacts with the sulfonate groups of two symmetry-related porphyrins. The porphyrin itself has 2/*m* site symmetry (the asymmetric unit contains one-quarter of the polyanion), sitting on an inversion center generated by the intersection of a mirror plane and a perpendicular twofold rotation axis. The porphyrin remains essentially planar (RMSD: 0.029 Å), but the pocket-bounding aryl groups have collapsed to reduce the pocket volume to 7.75 Å^3^ (from 23.25 Å^3^ in **2b**), highlighting the flexibility of the pockets.

### Metalation

**Figure 5:**
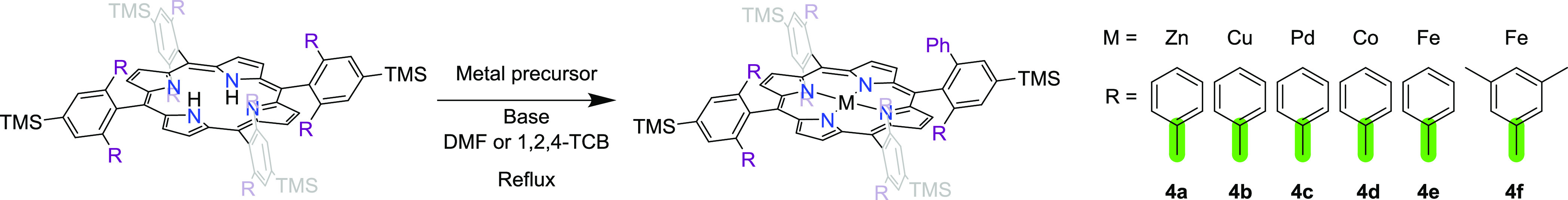
Metal insertion into bulky bis-pocket porphyrins.

Although free-base porphyrins can have a variety of valuable properties and reactivities, these molecules are perhaps most widely investigated as ligands for transition metals. The porphyrin scaffold provides a strong thermodynamic preference for metal binding, but the steric bulk of the bis-pocket architecture can provide a significant kinetic barrier to metalation. Although challenging porphyrin metalations are typically performed by heating the free-base ligand with a metal halide and a base in DMF, the original bis-pocket porphyrin report described a process whereby the ligand was heated with Fe(CO)_5_ and I_2_ followed by aqueous aerobic workup.^14^ In our previous work, we confirmed that this approach affords the Fe(III) complex of **2a**. Here we explored whether a more streamlined metalation strategy could be developed (Figure 5).

**Figure 6:**
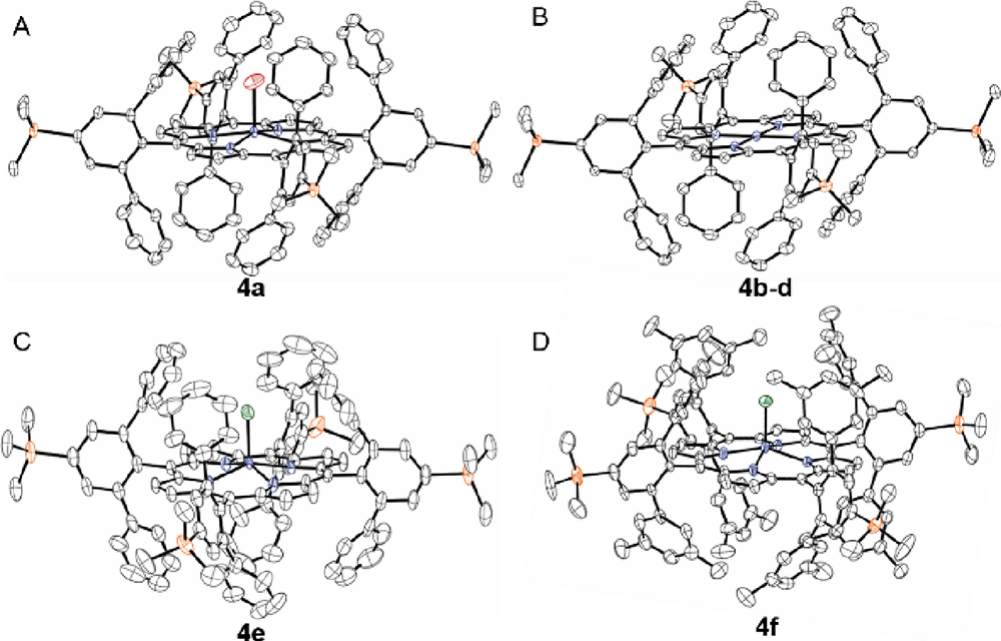
Thermal ellipsoid plots (50% probability level) of (A) the Zn-aqua complex **4a**, (B) the Cu complex **4b** (note that the Pd complex **4c** and the Co complex **4d** are isostructural), (C) the Fe-chloro complex **4e**, and (D) the Fe-chloro complex **4f**. H atoms, disorder, and solvent molecules are omitted for clarity. Color code: C gray, Si orange, O red, N blue, and Cl green metal purple.

The Zn(II) and Cu(II) complexes of **2a** could indeed be readily accessed using standard reaction conditions: refluxing the free-base and excess pyridine in DMF with excess Zn(OAc)_2_·2H_2_O and CuCl_2_·2H_2_O, respectively. The ^1^H NMR spectrum of the diamagnetic Zn(II) product **4a** shows the loss of the upfield N-H resonances, as compared to the spectrum of the free ligand, and subtle shifts in the aromatic signals. Additionally, the spectrum features a new singlet of 2H integration at −1.29 ppm. We hypothesized that this signal arises from the coordination of the Zn center to adventitious water as an aqua ligand. Square-planar coordination is disfavored for Zn(II), providing a strong driving force to coordinate even trace amounts of water. Although coordination to a Lewis acidic metal center would be expected to deshield the protons of the aqua ligand, the geometric position of these protons above the plane of the macrocycle would result in significant shielding from the diamagnetic porphyrin ring current, which is consistent with the upfield location of the resonance (−1.29 ppm). The presence of the aqua ligand was ultimately confirmed crystallographically (Figure 6) with a Zn–O bond length of 2.194(5) Å. The crystal structure also revealed the Zn center to lie 0.3196(6) Å above the plane of the porphyrin. The porphyrin itself is highly planar with an RMSD of 0.023 Å.

The paramagnetic nature of the Cu(II) complex **4b** precluded NMR spectroscopic characterization, but single-crystal X-ray diffraction from the red plates of the product confirmed the insertion of the metal (Figure 6). The Cu assumes a square-planar geometry with no axial ligand coordination. The primary coordination sphere is rigorously planar, as required by the crystallographic site symmetry of the complex. Beyond the primary coordination sphere of the metal, the porphyrin ligand retains a planar configuration with an RMSD from planarity of 0.018 Å.

Attempts to form the Pd(II) complex in the same way using Pd(OAc)_2_ were unsuccessful. Refluxing an excess of Pd(OAc)_2_ with ligand **2a** in 1,2,4-TCB, however, resulted in insertion into the macrocycle, as indicated by mass spectrometric analysis. We hypothesize that the higher refluxing temperature of 1,2,4-TCB as compared to DMF provides the greater activation energy needed to metalate the bulky porphyrin. The mass spectrometric analysis of the reaction mixture revealed, however, that side products featuring loss of TMS groups formed during the reaction, in addition to the desired product. We confirmed that the temperature of the reaction alone is not sufficient to induce desilylation of the starting porphyrin and therefore suspect that the Pd itself is effecting this transformation. By using only 1 equiv of Pd(OAc)_2_, the desilylation was decreased and the Pd(II) complex could be isolated in 25% yield. The complex is diamagnetic and features all of the expected resonances. Unlike the Zn(II) complex, there are no additional features to the spectrum, consistent with the square-planar geometry expected for a Pd(II) complex. Single-crystal X-ray diffraction revealed the Pd(II) complex to be isostructural with the Cu(II) complex.

Refluxing metal halide, lutidine, and **2a** in 1,2,4-TCB also permitted insertion of Co(II). The reaction proceeded smoothly, and the resulting paramagnetic Co(II) complex, **4d**, was isolated in 91% yield. X-ray crystallography confirmed the formation of a square-planar complex that is isostructural with the Pd(II) and Cu(II) complexes.

Finally, we investigated whether Fe could be inserted into **2a** directly using a metal halide as opposed to the circuitous route involving Fe(CO)_5_ described above. As we observed with Pd, refluxing the porphyrin ligand with an excess of FeCl_2_ and lutidine in DMF afforded no product. In contrast, refluxing 1,2,4-TCB cleanly produced 5,10,15,20-tetrakis(2,6-diphenyl-4-(trimethylsilyl)phenyl)porphyrinatochloroiron(III), **4e**. The ^1^H NMR spectrum of this paramagnetic complex clearly shows the characteristic β-pyrrole signal at 80.42 ppm. Single-crystal X-ray diffraction confirms the formation of the desired complex (Figure 6). The Fe center is displaced 0.492(3) Å from the plane of the porphyrin, which adopts a flat configuration (RMSD = 0.069 Å). In contrast to the Fe(CO)_5_/I_2_/alkaline hydrolysis procedure, which affords an Fe(III) hydroxide complex, the presently described reaction affords an Fe(III) chloride complex with an Fe–Cl bond length of 2.203(5) Å. The magnetic moment of **4e** was measured using the Evans method, which returned a value of 5.65 μ_B_. This value not only compares favorably with the value of 5.49 μ_B_, which was previously obtained for the hydroxoiron(III) complex of this same porphyrin,^21^ but also agrees better with the spin-only magnetic moment prediction for a high-spin d^5^ center (μ_s.o._ = 5.92 μ_B_) than that of any of the other accessible electronic configurations. We then demonstrated that this metalation protocol is also capable of producing the Fe(III) chloride derivative of **2d**, which bears the even more sterically encumbered 2,6-bis(3,5-dimethylphenyl)-4-trimethylsilylphenyl *meso* substituents. With this ligand, the Fe(CO)_5_/I_2_/alkaline hydrolysis method was unsuccessful, whereas refluxing **2d** with excess FeCl_2_ and 2,6-lutidine in 1,2,4-TCB affords the product **4f** in 71% yield. Again, crystallographic analysis (Figure 6) reveals that the Fe center is displaced from the plane of the porphyrin (0.4993(15) Å) and that the axial ligand is a chloride (Fe–Cl = 2.213(2) Å). Given that two equivalents of Cl^–^ are expected to be eliminated from the reaction as 2,6-lutidinium chloride, the Cl atom at the axial position is believed to come from cannibalized excess FeCl_2_. As with **4e**, the magnetic moment of **4f** (μ_eff_ = 6.59 μ_B_) again agrees best with a high-spin Fe(III) complex.

## Conclusions

We report that Pd-catalyzed Suzuki–Miyaura cross-coupling can be readily performed with an easily synthesized free-base porphyrin to access a range of novel porphyrins. This reaction proved versatile in that the steric and electronic properties of the resulting porphyrins could be readily tuned. Substituents featuring a variety of synthetic handles could be installed, rendering the bis-pocket porphyrin products amenable to further modification. The TMS groups of the precursor **1** and the products **2** impart organic solubility, which can be readily converted to aqueous solubility upon sulfonation with trimethylsilyl chlorosulfonate. Finally, metalation could be readily achieved using standard protocols for some metals or refluxing 1,2,4-TCB when necessary. Our tunable porphyrin platform offers a widely applicable scaffold for other researchers seeking to study or exploit the properties of these molecules.

## Experimental Section

### General Considerations

All reactions were performed under N_2_ unless otherwise specified. Glassware was oven-dried prior to use. All solvents and reagents are commercially available and used as received unless otherwise stated. Compound **1** was prepared as previously described.^21^ Suzuki–Miyaura reactions were performed in Chemglass 20 mL reaction vials fitted with pressure relief caps and heated on a hot plate fitted with a Chemglass 4-place pie wedge for 20 mL scintillation vials. For the purification of **3a** and **3b**, an Isolera Prime Biotage fitted with a Sfär C18 column was employed. A solution of 1% triethylammonium bicarbonate in water was generated by dissolving 40 mL of triethylamine in 4 L of ultra-pure (UP) water (>18 MΩ cm) followed by the addition of 150 g of dry ice. Analytical HPLC was performed on a Shimadzu Prominence-I LC-2030 Plus fitted with a Phenomenex Luna silica 5 μm 100 Å column (250 × 10 mm). Organic solutions were concentrated under reduced pressure on a Buchi Rotavapor R-100. CDCl_3_ and DMSO-*d*_6_ were purchased from Cambridge Isotope Laboratories and used as received. ^1^H, ^13^C{^1^H}, and ^19^F{^1^H} NMR spectra were recorded on a Bruker Avance III HD 500 NMR spectrometer equipped with a multinuclear Smart Probe. Signals in the ^1^H and ^13^C NMR spectra are reported in ppm as chemical shifts from tetramethylsilane; ^19^F NMR signals are reported in ppm as chemical shifts from CFCl_3_. NMR signals were referenced using the CHCl_3_ (^1^H, 7.26 ppm), DMSO-*d*_5_ (^1^H, 2.50 ppm), or CDCl_3_ (^13^C, 77.0 ppm) solvent signals. The following abbreviations were used to explain the multiplicities: s = singlet, d = doublet, t = triplet, q = quartet, m = multiplet, sext = sextet. Solution phase magnetic moments were measured using a modified Evans method.^30^ UV–visible absorption spectra were measured on a VWR UV-6300PC dual-beam spectrophotometer. MALDI mass spectra were acquired using timsControl v 1.1.19 on a timsTOF fleX mass spectrometer (Bruker Scientific, Billerica, MA) over the mass range 1000–2500 Da. In positive reflectron mode, laser power was set to 20%, and laser application was set to MS Dried Droplet. In negative reflectron mode, laser power was set to 30%, and laser application was set to MS Dried Droplet. Compounds were dissolved in DCM or MeOH, and 1 μL was mixed with 1 μL of the matrix (50:50 α-cyano-4-hydroxycinnamic acid/2,5-dihydroxybenzoic acid in a solution of 70:30 MeCN/H_2_O with 0.1% trifluoroacetic acid). Samples were spotted on a stainless steel MSP 96 spot target plate and allowed to air dry. For each compound, 1000 laser shots at 2000 Hz were delivered in a random walk across the spot. Data were subsequently analyzed in DataAnalysis v 5.3 (Bruker Scientific, Billerica, MA). The aqueous solubility of **3a** and **3b** was assessed by adding sufficient solid to a minimal volume of water such that a portion of the solid remained undissolved at room temperature, centrifuging the mixture, removing an aliquot from the supernatant, and determining the concentration of the compound in the aliquot using UV–vis spectroscopy.

### General Method for Suzuki–Miyaura Coupling

Compound **1** (100 mg, 0.0656 mmol), 1 mol % per carbon-bromine bond of palladium catalyst (0.0052 mmol), 3 equiv of boronic acid (1.574 mmol), and 4 equiv of cesium carbonate (2.09 mmol) were dissolved in a mixture of toluene (5 mL) and DI water (0.2 mL) in a 20 mL reaction vial fitted with a pressure relief cap. The mixture was sparged with N_2_ for 5 min, sealed, brought to 100 °C on a hot plate, and stirred for 20 h. The crude reaction mixture was dry loaded onto silica and purified by normal phase flash chromatography. The eluted product was concentrated under reduced pressure, redissolved in a minimal amount of chloroform, and recrystallized overnight via layering with MeCN. The resulting purple crystals were isolated via vacuum filtration.

### Synthesis of **2a** 5,10,15,20-Tetrakis(2,6-diphenyl-4-(trimethylsilyl)phenyl)porphyrin

Synthesized using the general method for Suzuki–Miyaura couplings. Column chromatography run as a ramp to 50% CHCl_3_ in hexanes. **2a** was isolated as purple crystals (88 mg, 89%). Characterization was consistent with previously reported data.^21^^1^H NMR (500 MHz, CDCl_3_) δ 8.38 (s, 8H), 7.77 (s, 8H), 6.55 (d, *J* = 7.9 Hz, 16H), 6.40 (t, *J* = 7.2 Hz, 8H), 6.22 (t, *J* = 7.5 Hz, 16H), 0.50 (s, 36H), −3.46 (s, 2H). ^13^C{^1^H} NMR (126 MHz, CDCl_3_) δ 144.8, 142.4, 140.8, 139.3, 133.6, 129.4, 126.7, 125.3, 116.1, −0.6; HRMS (MALDI) *m*/*z*: [M + H]^+^ calcd for C_104_H_95_N_4_Si_4_^+^ 1512.6662; found 1512.6669; UV/vis (CHCl_3_) λ_abs_ (log ε): 420 (sh), 439 (5.67), 533 (4.32), 570 (4.01), 611 (3.42), 669 (3.48).

### Synthesis of **2b** 5,10,15,20-Tetrakis(2,6-di(3,5-difluorophenyl)-4-(trimethylsilyl)phenyl)porphyrin

Synthesized using the general method for Suzuki–Miyaura couplings. Column chromatography run as a ramp to 40% CHCl_3_ in hexanes. **2b** was isolated as purple crystals (59 mg, 50%). X-ray quality crystals were grown by layering MeCN over a solution of the product in CHCl_3_. ^1^H NMR (500 MHz, CDCl_3_) δ 8.42 (s, 8H), 7.80 (s, 8H), 6.06 (d, *J* = 4.4 Hz, 16H), 5.87–5.79 (m, 8H), 0.54 (s, 36H), −3.16 (s, 2H). ^13^C{^1^H} NMR (126 MHz, CDCl_3_) δ 162.6, 162.5, 160.6, 160.5, 144.6, 143.2, 142.3, 138.7, 134.0, 115.7, 112.3, 112.1, 101.8, 101.6, 101.4, −0.7. ^19^F{^1^H} NMR (470 MHz, CDCl_3_) δ −111.49. HRMS (MALDI) *m*/*z*: [M + H]^+^ calcd for C_104_H_79_F_16_N_4_Si^+^ 1800.5155; found 1800.5142; UV/vis (CHCl_3_) λ_abs_ (log ε): 417 (sh), 435 (5.66), 430 (4.32), 565 (3.98), 608 (3.79), 667 (3.47).

### Synthesis of **2c** 5,10,15,20-Tetrakis(2,6-di(3,5-dichlorophenyl)-4-(trimethylsilyl)phenyl)porphyrin

Synthesized using the general method for Suzuki–Miyaura couplings but with Pd(PPh_3_)_4_ as the catalyst. Column chromatography run as a ramp to 10% toluene in pentane. **2c** was isolated as purple crystals (74 mg, 70%). X-ray quality crystals were grown by layering MeCN over a solution of the product in CHCl_3_. ^1^H NMR (500 MHz, CDCl_3_) δ 8.47 (s, 8H), 7.76 (s, 8H), 6.45 (d, *J* = 1.8 Hz, 16H), 6.39 (t, *J* = 1.8 Hz, 8H), 0.54 (s, 36H), −2.93 (s, 2H). ^13^C{^1^H} NMR (126 MHz, CDCl_3_) δ 144.3, 142.7, 142.4, 138.2, 134.9, 133.5, 127.5, 126.3, 115.6, −0.7. HRMS (MALDI) *m*/*z*: [M + H]^+^ calcd for C_104_H_79_Cl_16_N_4_Si_4_^+^ 2064.0309; found 2064.0280; UV/vis (CHCl_3_) λ_abs_ (log ε): 419 (sh), 438 (5.58), 490 (4.06), 530 (4.26), 567 (3.91), 608 (3.72), 668 (3.41).

### Synthesis of **2d** 5,10,15,20-Tetrakis(2,6-di(3,5-dimethylphenyl)-4-(trimethylsilyl)phenyl)porphyrin

Synthesized using the general method for Suzuki–Miyaura couplings. Column chromatography was performed as a ramp to 30% CHCl_3_ in hexanes. **2d** was isolated as purple crystals (108 mg, 95%). X-ray quality crystals were grown by layering MeCN over the product in 1,2,4-TCB to give purple plates. ^1^H NMR (500 MHz, CDCl_3_) δ 8.51 (s, 8H), 7.71 (s, 8H), 6.20 (s, 16H), 5.92 (s, 8H), 1.15 (s, 48H), 0.49 (s, 36H), −3.04 (s, 2H). ^13^C{^1^H} NMR (126 MHz, CDCl_3_) δ 145.1, 142.5, 140.3, 138.7, 135.7, 134.6, 127.5, 127.3, 116.7, 20.7, −0.6. HRMS (MALDI) *m*/*z*: [M + H]^+^ calcd for C_120_H_127_N_4_Si_4_^+^ 1736.9166; found 1736.9185; UV/vis (CHCl_3_) λ_abs_ (log ε): 418 (sh), 437 (5.66), 532 (4.28), 567 (3.99), 609 (3.74),669 (3.44).

### Synthesis of **2e** 5,10,15,20-Tetrakis(2,6-di(4-methylphenyl)-4-(trimethylsilyl)phenyl)porphyrin

Synthesized using the general method for Suzuki–Miyaura couplings. Column chromatography was performed as a ramp to 40% CHCl_3_ in hexanes. **2e** was isolated as purple crystals (76 mg, 71%). X-ray quality crystals were grown by layering MeCN over the product in toluene to give purple plates. ^1^H NMR (500 MHz, CDCl_3_) δ 8.43 (s, 8H), 7.72 (s, 8H), 6.51 (d, *J* = 8.1 Hz, 16H), 6.05 (d, *J* = 8.1 Hz, 16H), 1.71 (s, 24H), 0.50 (s, 36H), −3.31 (s, 2H). ^13^C{^1^H} NMR (126 MHz, CDCl_3_) δ 144.7, 140.5, 139.8, 139.3, 134.3, 133.7, 129.3, 127.4, 116.3, 20.8, −0.6. HRMS (MALDI) *m*/*z*: [M + H]^+^ calcd for C_112_H_111_N_4_Si_4_^+^ 1624.7914; found 1624.7885; UV/vis (CHCl_3_) λ_abs_ (log ε): 420 (sh), 439 (5.64), 533 (4.28), 569 (3.95), 610 (3.75), 671 (3.39).

### Synthesis of **2f** 5,10,15,20-Tetrakis(2,6-di(4-*N*-propylphenyl)-4-(trimethylsilyl)phenyl)porphyrin

Synthesized using the general method for Suzuki–Miyaura couplings. Column chromatography was performed as a ramp to 20% diethyl ether in hexanes. **2f** was not recrystallized and was isolated as a purple solid (85 mg, 70%). X-ray quality crystals were grown by layering a one-to-one mixture of EtOH and MeCN over the product in CHCl_3_ to give purple needles. ^1^H NMR (500 MHz, CDCl_3_) δ 8.38 (s, 8H), 7.74 (s, 8H), 6.46 (d, *J* = 7.5 Hz, 16H), 6.03 (d, *J* = 8.2 Hz, 16H), 1.94–1.82 (m, 16H), 1.11 (sext, *J* = 7.4 Hz, 16H), 0.53–0.45 (m, 60H), −3.28 (s, 2H). ^13^C{^1^H} NMR (126 MHz, CDCl_3_) δ 140.0, 139.1, 133.8, 129.3, 126.7, 37.7, 24.4, 13.8, −0.6. HRMS (MALDI) *m*/*z*: [M + H]^+^ calcd for C_128_H_143_N_4_Si_4_^+^ 1849.0417; found 1849.0427; UV/vis (CHCl_3_) λ_abs_ (log ε): 420 (sh), 440 (5.63), 534 (4.27), 570 (3.95), 611 (3.74), 671 (3.37).

### Synthesis of **2g** 5,10,15,20-Tetrakis(2,6-di(4-*tert*-butylphenyl)-4-(trimethylsilyl)phenyl)porphyrin

Synthesized using the general method for Suzuki–Miyaura couplings but with the reaction time extended to 48 h. Column chromatography was performed as a ramp to 5% CHCl_3_ in hexanes. **2g** was not recrystallized and was isolated as a purple solid (57 mg, 44%). ^1^H NMR (500 MHz, CDCl_3_) δ 8.38 (s, 8H), 7.82 (s, 8H), 6.42–6.30 (m, 32H), 0.77 (s, 72H), 0.52 (s, 36H), −2.97 (s, 2H). ^13^C{^1^H} NMR (126 MHz, CDCl_3_) δ 147.7, 145.1, 140.5, 140.0, 138.7, 135.3, 129.5, 124.0, 116.1, 33.9, 31.3, −0.6. HRMS (MALDI) *m*/*z*: [M + H]^+^ calcd for C_136_H_159_N_4_Si_4_^+^ 1961.1669; found 1961.1690; UV/vis (CHCl_3_) λ_abs_ (log ε): 422 (sh), 443 (5.68), 537 (4.30), 573 (4.07), 612 (3.79), 672 (3.43).

### Synthesis of **2h** 5,10,15,20-Tetrakis(2,6-di(4-fluorophenyl)-4-(trimethylsilyl)phenyl)porphyrin

Synthesized using the general method for Suzuki–Miyaura couplings. Column chromatography was performed as a ramp to 30% CHCl_3_ in hexanes. **2h** was isolated as purple crystals (67 mg, 62%). X-ray quality crystals were grown by layering MeCN over the product in toluene at −20 °C to give purple plates. ^1^H NMR (500 MHz, CDCl_3_) δ 8.38 (s, 8H), 7.75 (s, 8H), 6.52 (dd, *J* = 8.7, 5.4 Hz, 16H), 5.95 (t, *J* = 8.7 Hz, 16H), 0.52 (s, 36H), −3.35 (s, 2H). ^13^C{^1^H} NMR (126 MHz, CDCl_3_) δ 161.6, 159.7, 143.9, 141.4, 139.2, 138.2, 138.2, 133.8, 130.9, 130.8, 116.3, 113.7, 113.5, −0.7. ^19^F{^1^H} NMR (470 MHz, CDCl_3_) δ −116.48. HRMS (MALDI) *m*/*z*: [M + H]^+^ calcd for C_104_H_87_F_8_N_4_Si_4_^+^ 1656.5909; found 1656.5879; UV/vis (CHCl_3_) λ_abs_ (log ε): 419 (sh), 439 (5.66), 533 (4.30), 569, (3.99), 611 (3.76), 670 (3.45).

### Synthesis of **2i** 5,10,15,20-Tetrakis(2,6-di(4-trifluoromethylphenyl)-4-(trimethylsilyl)phenyl)porphyrin

Synthesized using the general method for Suzuki–Miyaura couplings. Column chromatography was performed as a ramp to 10% CHCl_3_ in hexanes. **2i** was not recrystallized and was isolated as a purple solid (37 mg, 27%). X-ray quality purple needles were grown by layering MeCN over a solution of the product in CHCl_3_. ^1^H NMR (500 MHz, CDCl_3_) δ 8.40 (s, 8H), 7.80 (s, 8H), 6.59 (d, *J* = 8.1 Hz, 16H), 6.47 (d, *J* = 8.2 Hz, 16H), 0.52 (s, 36H), −3.33 (s, 2H). ^19^F{^1^H} NMR (470 MHz, CDCl_3_) δ −63.27. ^13^C{^1^H} NMR (126 MHz, CDCl_3_) δ 145.5, 143.8, 142.2, 138.5, 134.5, 129.5, 128.2, 128.0, 126.7, 126.5, 124.5, 123.6, 122.3, 120.2, 115.9, −0.7. HRMS (MALDI) *m*/*z*: [M + H]^+^ calcd for C_112_H_87_F_24_N_4_Si_4_^+^ 2056.5652; found 2056.5643; UV/vis (CHCl_3_) λ_abs_ (log ε): 418 (sh), 437 (5.61), 495 (3.65), 531 (4.26), 567 (3.96), 608 (3.79), 668 (3.48), 700 (2.98).

### Synthesis of **2j** 5,10,15,20-Tetrakis(2,6-di(4-nitrophenyl)-4-(trimethylsilyl)phenyl)porphyrin

Synthesized using the general method for Suzuki–Miyaura couplings but with Pd(PPh_3_)_4_ as the catalyst, 4 equiv of boronic acid, and 48 h reaction time. Column chromatography was performed as a slow ramp from 100% hexanes to 100% CHCl_3_. **2j** was not recrystallized and isolated as a purple solid (5.7 mg, 5%). ^1^H NMR (500 MHz, CDCl_3_) δ 8.38 (s, 8H), 7.84 (s, 8H), 7.11 (d, *J* = 8.9 Hz, 16H), 6.58 (d, *J* = 8.9 Hz, 16H), 0.54 (s, 36H), −3.33 (s, 2H). ^13^C{^1^H} NMR (126 MHz, CDCl_3_) δ 148.2, 145.9, 143.3, 137.9, 135.0, 130.1, 122.1, 115.9, −0.8. HRMS (MALDI) *m*/*z*: [M + H]^+^ calcd for C_104_H_87_N_12_O_16_Si_4_^+^ 1872.5468; found 1872.5437; UV/vis (CHCl_3_) λ_abs_ (log ε): 423 (sh), 445 (5.42), 536 (4.14), 573 (3.85), 612 (3.60), 671 (3.21).

### Synthesis of **2k** 5,10,15,20-Tetrakis(2,6-di(4-methoxyphenyl)-4-(trimethylsilyl)phenyl)porphyrin

Synthesized using the general method for Suzuki–Miyaura couplings but with diglyme as the solvent. After reaction completion, the crude mixture was diluted with hexanes (50 mL) and loaded onto a silica column. The loaded column was washed with hexanes, then 100 mL of 1:1 CHCl_3_/hexanes, and then 100 mL of 100% CHCl_3_. The product was then eluted by ramping to 15% MeOH in CHCl_3_. **2k** was not recrystallized and was isolated as a purple solid (92 mg, 80%). X-ray quality crystals were grown by layering MeCN over the product in CHCl_3_ to give purple needles. ^1^H NMR (500 MHz, CDCl_3_) δ 8.40 (s, 8H), 7.72 (s, 8H), 6.46 (d, *J* = 8.8 Hz, 16H), 5.78 (d, *J* = 8.9 Hz, 16H), 3.17 (s, 24H), 0.50 (s, 36H), −3.27 (s, 2H). ^13^C{^1^H} NMR (126 MHz, CDCl_3_) δ 157.2, 144.5, 140.5, 139.6, 135.0, 133.5, 130.4, 116.5, 112.2, 54.6, −0.6. HRMS (MALDI) *m*/*z*: [M + H]^+^ calcd for C_112_H_111_N_4_O_8_Si_4_^+^ 1752.7507; found 1752.7490; UV/vis (CHCl_3_) λ_abs_ (log ε): 422 (sh), 442 (5.57), 497 (3.76), 535 (4.23), 572 (3.97), 612 (3.74), 671 (3.45).

### Synthesis of **2l** 5,10,15,20-Tetrakis(2,6-di(2-napthyl)-4-(trimethylsilyl)phenyl)porphyrin

Synthesized using the general method for Suzuki–Miyaura couplings. Column chromatography was performed as a ramp to 50% CHCl_3_ in hexanes. **2l** was isolated as purple crystals (85.3 mg, 68%). X-ray quality crystals were grown by layering MeCN over a solution of the product in CHCl_3_. ^1^H NMR (500 MHz, CDCl_3_) δ 8.47 (s, 8H), 7.79 (s, 8H), 7.32 (s, 8H), 7.13–7.06 (m, 16H), 6.94 (t, *J* = 7.3 Hz, 8H), 6.81 (d, *J* = 8.3 Hz, 8H), 5.94 (d, *J* = 8.3 Hz, 8H), 5.26 (d, *J* = 8.6 Hz, 8H), 0.51 (s, 36H), −3.56 (s, 2H). ^13^C{^1^H} NMR (126 MHz, CDCl_3_) δ 144.8, 140.9, 140.1, 139.6, 134.1, 132.4, 131.0, 127.8, 127.5, 127.2, 126.9, 125.3, 125.1, 116.0, −0.6. HRMS (MALDI) *m*/*z*: [M + H]^+^ calcd for C_136_H_111_N_4_Si_4_^+^ 1912.7913; found 1912.7938; UV/vis (CHCl_3_) λ_abs_ (log ε): 423 (sh), 444 (5.58), 536 (4.24), 571 (3.93), 611 (3.72), 671 (3.38).

### Synthesis of **2m** 5,10,15,20-Tetrakis(2,6-dicyclopropyl-4-(trimethylsilyl)phenyl)porphyrin

Synthesized using the general method for Suzuki–Miyaura couplings. Column chromatography was performed as a ramp to 50% CHCl_3_ in hexanes. **2m** was not recrystallized and was isolated as a purple solid (26 mg, 32%). X-ray quality crystals were grown by layering MeCN over the product in CHCl_3_ to give purple needles. ^1^H NMR (500 MHz, CDCl_3_) δ 8.66 (s, 8H), 7.22 (s, 8H), 1.15–1.09 (m, 8H), 0.67–0.61 (m, 16H), 0.46 (s, 36H), 0.05 to −0.03 (m, 16H), −2.29 (s, 2H). ^13^C{^1^H} NMR (126 MHz, CDCl_3_) δ 143.4, 143.3, 140.5, 125.2, 117.3, 15.8, 8.7, −0.6. HRMS (MALDI) *m*/*z*: [M + H]^+^ calcd for C_80_H_95_N_4_Si_4_^+^ 1223.6628; found 1223.6599; UV/vis (CHCl_3_) λ_abs_ (log ε): 404 (sh), 422 (5.55), 516 (4.16), 550 (3.62), 591 (3.63), 646 (3.26).

### Synthesis of **2n** 5,10,15,20-Tetrakis(2,6-di(4-vinylphenyl)-4-(trimethylsilyl)phenyl)porphyrin

Synthesized using the general method for Suzuki–Miyaura couplings. Column chromatography was performed as a ramp to 30% CHCl_3_ in hexanes. **2n** was not recrystallized and was isolated as a purple solid (37 mg, 33%). ^1^H NMR (500 MHz, CDCl_3_) δ 8.42 (s, 8H), 7.76 (s, 8H), 6.49 (d, *J* = 8.2 Hz, 16H), 6.27 (d, *J* = 8.3 Hz, 16H), 6.03 (dd, *J* = 17.6, 10.9 Hz, 8H), 5.14 (d, *J* = 17.5 Hz, 8H), 4.77 (d, *J* = 11.3 Hz, 8H), 0.51 (s, 36H), −3.27 (s, 2H). ^13^C{^1^H} NMR (126 MHz, CDCl_3_) δ 144.7, 142.1, 140.9, 139.2, 136.3, 134.4, 133.8, 129.6, 124.8, 116.2, 113.1, −0.7. HRMS (MALDI) *m*/*z*: [M + H]^+^ calcd for C_120_H_111_N_4_Si_4_^+^ 1720.7914; found 1720.7893; UV/vis (CHCl_3_) λ_abs_ (log ε): 422 (sh), 443 (5.38), 536 (4.05), 571 (3.76), 612 (3.55), 672 (3.25).

### Synthesis of **2o** 5,10,15,20-Tetrakis(2,6-di(4-trimethylsilylphenyl)-4-(trimethylsilyl)phenyl)porphyrin

Synthesized using the general method for Suzuki–Miyaura couplings but with 4 equiv of boronic acid and an increased reaction time of 48 h. Column chromatography was performed as a ramp to 5% toluene in hexanes. **2o** was not recrystallized and was isolated as a purple solid (64 mg, 47%). X-ray quality crystals were grown by layering a one-to-one mixture of EtOH and MeCN over the product in CHCl_3_ to give purple needles. ^1^H NMR (500 MHz, CDCl_3_) δ 8.38 (s, 8H), 7.79 (s, 8H), 6.52 (d, *J* = 7.6 Hz, 16H), 6.42 (d, *J* = 7.4 Hz, 16H), 0.50 (s, 36H), −0.26 (s, 72H), −3.05 (s, 2H). ^13^C{^1^H} NMR (126 MHz, CDCl_3_) δ 145.4, 143.5, 140.6, 138.5, 136.5, 135.7, 132.3, 129.3, 115.7, −0.6, −0.9. HRMS (MALDI) *m*/*z*: [M + H]^+^ calcd for C_128_H_159_N_4_Si_12_^+^ 2088.9824; found 2088.9763; UV/vis (CHCl_3_) λ_abs_ (log ε): 424 (sh), 444 (5.63), 538 (4.31), 574 (4.07), 612 (3.85), 671 (3.52).

### Synthesis of **2p** 5,10,15,20-Tetrakis(2,6-di(4-ethoxycarbonylphenyl)-4-(trimethylsilyl)phenyl)porphyrin

Synthesized using the general method for Suzuki–Miyaura couplings but with diglyme as the solvent and 4 equiv of boronic acid. After reaction completion, the crude mixture was diluted with hexanes (50 mL) and loaded onto a silica column. The loaded column was washed with hexanes, then 100 mL of 1:1 CHCl_3_/hexanes, and then 100 mL of 100% CHCl_3_. The product was then eluted by ramping to 15% MeOH in CHCl_3_. **2p** was not recrystallized and was isolated as a purple solid (55 mg, 40%). X-ray quality crystals were grown by layering MeCN over the product in toluene at −20 °C to give purple plates. ^1^H NMR (500 MHz, CDCl_3_) δ 8.30 (s, 8H), 7.80 (s, 8H), 6.95 (d, *J* = 8.0 Hz, 16H), 6.47 (d, *J* = 7.9 Hz, 16H), 3.92 (q, *J* = 7.0 Hz, 16H), 0.96 (t, *J* = 7.0 Hz, 24H), 0.53 (s, 36H), −3.31 (s, 2H). ^13^C{^1^H} NMR (126 MHz, CDCl_3_) δ 165.7, 146.8, 144.5, 141.6, 138.6, 134.8, 129.6, 128.1, 127.8, 115.6, 60.5, 14.1, −0.7. HRMS (MALDI) *m*/*z*: [M + H]^+^ calcd for C_128_H_127_N_4_O_16_Si_4_^+^ 2088.8353; found 2088.8318; UV/vis (CHCl_3_) λ_abs_ (log ε): 424 (sh), 444 (5.64), 537 (4.31), 574 (4.09), 613 (3.83), 672 (3.61).

### Synthesis of **2q** 5,10,15,20-Tetrakis(2,6-di(*N*-methylpyrazolyl)-4-(trimethylsilyl)phenyl)porphyrin

Synthesized using the general method for Suzuki–Miyaura couplings but with diglyme as the solvent and 4 equiv of boronic acid. After reaction completion, the crude mixture was diluted with hexanes (50 mL) and loaded onto a silica column. The loaded column was washed with hexanes, then 100 mL of 1:1 CHCl_3_/hexanes, and then 100 mL of 100% CHCl_3_. The product was then eluted by ramping to 15% MeOH in CHCl_3_. **2q** was not recrystallized and was isolated as a purple solid (81 mg, 80%). X-ray quality crystals were grown by layering diethyl ether over the product in CHCl_3_ to give purple plates. ^1^H NMR (500 MHz, CDCl_3_) δ 8.51 (s, 8H), 7.79 (s, 8H), 6.29 (s, 8H), 5.73 (s, 8H), 2.88 (s, 24H), 0.51 (s, 36H), −2.64 (s, 2H). ^13^C{^1^H} NMR (126 MHz, CDCl_3_) δ 141.4, 138.0, 137.6, 135.6, 131.7, 128.6, 123.0, 118.0, 38.2, −0.7. HRMS (MALDI) *m*/*z*: [M + H]^+^ calcd for C_88_H_95_N_20_Si_4_^+^ 1543.7120; found 1543.7139; UV/vis (CHCl_3_) λ_abs_ (log ε): 412 (sh), 431 (5.57), 524 (4.27), 559 (3.71), 598 (3.78), 656 (3.4).

### Larger-Scale Synthesis of **2h** 5,10,15,20-Tetrakis(2,6-di(4-fluorophenyl)-4-(trimethylsilyl)phenyl)porphyrin

This procedure demonstrates that the coupling can be performed on a scale such that 1 mmol of arylboronic acid is coupled to the porphyrin framework. Compound **1** (200 mg, 0.1311 mmol), 1 mol % per carbon-bromine bond of (dppf)PdCl_2_ (0.0105 mmol), 3 equiv of 4-fluorophenylboronic acid (3.1455 mmol), and 4 equiv of cesium carbonate (4.1940 mmol) were dissolved in a mixture of toluene (10 mL) and DI water (0.4 mL) in a 20 mL reaction vial fitted with a pressure relief cap. The mixture was sparged with N_2_ for 5 min, sealed, brought to 100 °C, and stirred for 20 h. The crude reaction mixture was dry loaded onto silica and purified by normal phase flash chromatography. Column chromatography was performed as a ramp to 30% CHCl_3_ in hexanes. The eluted product was concentrated under reduced pressure, redissolved in a minimal amount of CHCl_3_, and recrystallized overnight via layering with MeCN. The resulting purple crystals were isolated via vacuum filtration. **2h** was isolated as purple crystals (154 mg, 71%).

### Synthesis of **3a** Sodium 5,10,15,20-Tetrakis(2,6-diphenyl-4-(sulfonato)phenyl)porphyrin

Compound **2a** (50 mg, 0.0331 mmol) and 4.8 equiv of trimethylsilyl chlorosulfonate (24 μL, 0.1589 mmol) were dissolved in carbon tetrachloride (5 mL) in a 20 mL reaction vial fitted with a pressure release cap. The reaction was sealed and incubated at 75 °C for 2 h. The reaction was removed from heat, quenched with 5 mL of 1 M NaOH_(aq)_, and stirred vigorously for 30 min. The crude mixture was concentrated under reduced pressure and purified by reverse phase flash column chromatography using a ramp to 95% MeCN in water with 1% triethylammonium bicarbonate. The eluted product was diluted with 20 mL of brine and dialyzed overnight against DI water through a 3.5 kDa MWCO membrane. The solution was concentrated under reduced pressure, and **3a** was isolated as a purple solid (32 mg, 60%). ^1^H NMR (500 MHz, DMSO-*d*_6_) δ 8.41 (s, 8H), 7.87 (s, 8H), 6.50 (d, *J* = 7.7 Hz, 16H), 6.42 (t, *J* = 7.2 Hz, 8H), 6.28 (t, *J* = 7.5 Hz, 16H), −3.69 (s, 2H). ^13^C{^1^H} NMR (126 MHz, DMSO-*d*_6_) δ 148.2, 144.4, 141.1, 137.7, 128.5, 126.8, 125.7, 125.6, 115.5; HRMS (MALDI) *m*/*z*: [M – 4Na + 3H]^−^ calcd for C_92_H_61_N_4_O_12_S_4_^–^ 1541.3174; found 1541.3159; UV/vis (H_2_O) λ_abs_ (log ε): 416 (sh), 435 (5.29), 529 (3.94), 565 (3.48), 606 (3.40), 665 (2.96).

### Synthesis of **3b** Sodium 5,10,15,20-Tetrakis(2,6-di(3,5-difluorophenyl)-4-(sulfonato)phenyl)porphyrin

Compound **2b** (50 mg, 0.0278 mmol) and 12 equiv of trimethylsilyl chlorosulfonate (51 μL, 0.334 mmol) were dissolved in carbon tetrachloride (5 mL) in a 20 mL reaction vial fitted with a pressure release cap. The reaction was sealed and incubated at 75 °C for 1 h. The reaction was taken off heat, quenched with 5 mL of 1 M NaOH_(aq)_, and stirred vigorously for 30 min. The crude mixture was concentrated under reduced pressure and purified by reverse phase flash column chromatography using a ramp to 95% MeCN in water with 1% triethylammonium bicarbonate. The eluted product was diluted with 20 mL of brine and dialyzed overnight against DI water through a 3.5 kDa MWCO membrane. The solution was concentrated under reduced pressure, and **3b** was isolated as a purple solid (45 mg, 84%). X-ray quality crystals were grown by vapor diffusion of diethyl ether into a solution of the product 1:1 MeOH/diglyme to give purple plates. HRMS (MALDI) *m*/*z*: [M – 4Na + 3H]^−^ calcd for C_92_H_45_F_16_N_4_O_12_S_4_^–^ 1829.1667; found 1829.1639; ^1^H NMR (500 MHz, DMSO-*d*_6_) δ 8.58 (s, 8H), 7.96 (s, 8H), 6.29–6.01 (m, 24H), −3.44 (s, 2H). ^19^F{^1^H} NMR (470 MHz, DMSO-*d*_6_) δ −110.91. ^13^C{^1^H} NMR (126 MHz, DMSO-*d*_6_) δ 161.9, 161.8, 159.9, 159.8, 148.9, 143.9, 142.5, 137.3, 126.3, 114.8, 111.7, 111.5, 101.9, 101.7, 101.5; UV/vis (H_2_O) λ_abs_ (log ε): 414 (sh), 432 (5.29), 527 (3.94), 568 (3.48), 607 (3.40), 665 (3.36).

### Synthesis of **4a** 5,10,15,20-Tetrakis(2,6-diphenyl-4-(trimethylsilyl)phenyl)porphyrinatoaquazinc(II)

Compound **2a** (50 mg, 0.033 mmol), zinc(II) acetate dihydrate (700 mg, 3.20 mmol), pyridine (0.1 mL), DMF (5 mL), and a stir bar were added to a 15 mL round-bottom flask outfitted with a reflux condenser under a stream of nitrogen. The reaction was heated to reflux in an oil bath and allowed to stir overnight (16 h). UV–vis spectroscopy was used to confirm product formation. The reaction was diluted with water, and the resulting precipitate was collected by vacuum filtration. The solid was purified by column chromatography (silica gel, CHCl_3_/hexanes 1:1). The product fractions were concentrated under reduced pressure to give the product as a blue-purple solid (40 mg, 76%). X-ray quality crystals were grown by layering MeCN over the product dissolved in CHCl_3_. ^1^H NMR (500 MHz, CDCl_3_) δ 8.48 (s, 8H), 7.76 (s, 8H), 6.62 (d, *J* = 7.1 Hz, 16H), 6.37 (t, *J* = 7.1 Hz, 8H), 6.20 (t, *J* = 7.2 Hz, 16H), 0.51 (s, 36H), −1.29 (s, 2H). ^13^C{^1^H} NMR (126 MHz, CDCl_3_) δ 149.8, 144.6, 142.9, 140.3, 133.4, 131.5, 129.5, 126.4, 125.1, 116.7, −0.6; HRMS (MALDI) *m*/*z*: [M + H–OH]^+^ calcd for C_104_H_93_N_4_Si_4_^+^ 1574.5796; found 1574.5760; UV/vis (CHCl_3_) λ_abs_ (log ε): 422 (sh), 444 (5.58), 572 (4.22), 613 (3.50).

### Synthesis of **4b** 5,10,15,20-Tetrakis(2,6-diphenyl-4-(trimethylsilyl)phenyl)porphyrinatocopper(II)

Compound **2a** (50 mg, 0.033 mmol), copper(II) chloride dihydrate (563 mg, 3.30 mmol), pyridine (0.1 mL), DMF (5 mL), and a stir bar were added to a 15 mL round-bottom flask outfitted with a reflux condenser under a stream of nitrogen. The reaction was heated to reflux in an oil bath and allowed to stir overnight (16 h). UV–vis spectroscopy was used to confirm product formation. The reaction was diluted with water, and the resulting precipitate was collected by vacuum filtration. The solid was purified by column chromatography (silica gel, CHCl_3_/hexanes 1:1). The product fractions were concentrated under reduced pressure to give the product as a red solid (51 mg, 98%). X-ray quality crystals were grown by layering MeCN over the product dissolved in CHCl_3_. HRMS (MALDI) *m*/*z*: [M + H]^+^ calcd for C_104_H_93_CuN_4_Si_4_^+^ 1573.5801; found 1573.5765; μ_eff_ (Evans, CDCl_3_): 1.94 μ_B_; UV/vis (CHCl_3_) λ_abs_ (log ε): 414 (sh), 436 (5.49), 557 (4.25). HPLC (silica, hexane/DCM = ramp to 100% DCM, flow rate = 3.0 mL/min, λ = 400 nm) *t*_R_ = 11.7 min (96%).

### Synthesis of **4c** 5,10,15,20-Tetrakis(2,6-diphenyl-4-(trimethylsilyl)phenyl)porphyrinatopalladium(II)

Compound **2a** (52 mg, 0.034 mmol), palladium(II) acetate (5.7 mg, 0.033 mmol), 2,6-lutidine (0.1 mL), 1,2,4-TCB (5 mL), and a stir bar were added to a 15 mL round-bottom flask outfitted with a reflux condenser under a stream of nitrogen. The reaction was heated to reflux in an oil bath and allowed to stir overnight (16 h). UV–vis spectroscopy was used to confirm product formation. The crude reaction mixture was diluted with hexanes (50 mL), wet loaded onto a silica column, and purified by column chromatography (silica gel, CHCl_3_/hexanes, ramp chloroform to 1:1). The product fractions were concentrated under reduced pressure to give the product as a red-purple solid (13 mg, 25%). X-ray quality crystals were grown by layering MeCN over the product dissolved in CHCl_3_. ^1^H NMR (500 MHz, CDCl_3_) δ 8.33 (s, 8H), 7.76 (s, 8H), 6.50 (d, *J* = 7.8 Hz, 16H), 6.40 (t, *J* = 7.3 Hz, 8H), 6.21 (t, *J* = 7.6 Hz, 16H), 0.49 (s, 36H). ^13^C{^1^H} NMR (126 MHz, CDCl_3_) δ 144.5, 142.3, 141.4, 133.6, 130.6, 129.3, 126.6, 125.2, 117.9, −0.6; HRMS (MALDI) *m*/*z*: [M + H]^+^ calcd for C_104_H_93_N_4_PdSi_4_^+^ 1616.5544; found 1616.5521; UV/vis (CHCl_3_) λ_abs_ (log ε): 437 (5.2), 532 (sh), 541 (4.11), 573 (2.94).

### Synthesis of **4d** 5,10,15,20-Tetrakis(2,6-diphenyl-4-(trimethylsilyl)phenyl)porphyrinatocobalt(II)

Compound **2a** (100 mg, 0.0662 mmol), 10 equiv of 2,6-lutidine (0.662 mmol), and 100 equiv of cobalt(II) chloride hexahydrate (6.62 mmol) were dissolved in 1,2,4-TCB (5 mL) in a 20 mL reaction vial fitted with a pressure relief cap. The reaction mixture was heated at 213 °C without the cap for 30 min to remove water. The reaction was then sealed and allowed to stir at 213 °C for 2 h on a hot plate fitted with a Chemglass 4-place pie wedge for 20 mL scintillation vials. The crude reaction mixture was diluted with hexanes (50 mL), wet loaded onto a silica column, and purified by normal phase flash chromatography. The product was eluted in a 1:1 solvent mixture of hexanes/CHCl_3_ and concentrated under reduced pressure to yield the isolated product as a red solid (96 mg, 91%). X-ray quality crystals were grown by layering MeCN over the product in CHCl_3_ to give red plates. HRMS (MALDI) *m*/*z*: [M + H]^+^ calcd for C_104_H_93_CoN_4_Si_4_^+^ 1569.5838; found 1569.5807; μ_eff_ (Evans, CDCl_3_): 1.99 μ_B_; UV/vis (CHCl_3_) λ_abs_ (log ε): 432 (5.29), 546 (4.15). HPLC (silica, hexane/DCM = ramp to 100% DCM, flow rate = 3.0 mL/min, λ = 400 nm) *t*_R_ = 11.1 min (97%).

### Synthesis of **4e** 5,10,15,20-Tetrakis(2,6-diphenyl-4-(trimethylsilyl)phenyl)porphyrinatochloroiron(III)

Compound **2a** (73 mg, 0.0483 mmol), 10 equiv of 2,6-lutidine (0.483 mmol), and 100 equiv of iron(II) chloride (4.83 mmol) were dissolved in 1,2,4-TCB (5 mL) under an aerobic atmosphere in a 20 mL reaction vial fitted with a pressure relief cap. The reaction mixture was sealed and heated to 213 °C for 1 h on a hot plate fitted with a Chemglass 4-place pie wedge for 20 mL scintillation vials. The crude reaction mixture was diluted with hexanes, loaded onto a silica column, and purified by normal phase flash chromatography. The product was eluted in a 1:1 solvent mixture of hexanes/CHCl_3_ and concentrated under reduced pressure to yield the isolated product as deep purple crystals (63 mg, 82%). X-ray quality crystals were grown by layering MeCN over the product dissolved in CHCl_3_. ^1^H NMR (500 MHz, CDCl_3_; paramagnetic) δ 80.42 (β-pyrrole); μ_eff_ (Evans, CDCl_3_): 5.65 μ_B_; UV/vis (CHCl_3_) λ_abs_ (log ε): 361 (4.56), 372 (sh), 444 (5.17), 552 (3.86), 579 (3.72), 593 (sh), 678 (sh), 707 (3.61); HRMS (MALDI) *m*/*z*: [M + H]^+^ calcd for C_104_H_93_ClFeN_4_Si_4_^+^ 1601.5543; found 1601.5447; [M – Cl]^+^ calcd for C_104_H_92_FeN_4_Si_4_^+^ 1565.5776; found 1565.5761. HPLC (silica, hexane/DCM = ramp to 100% DCM, flow rate = 3.0 mL/min, λ = 440 nm) *t*_R_ = 8.7 min (99%).

### Synthesis of **4f** 5,10,15,20-Tetrakis(2,6-di(3,5-dimethylphenyl)-4-(trimethylsilyl)phenyl)porphyrinatochloroiron(III)

Compound **2d** (49 mg, 0.0281 mmol), 10 equiv of 2,6-lutidine (0.281 mmol), and 100 equiv of iron(II) chloride (1.40 mmol) were dissolved in 1,2,4-TCB (5 mL) under an aerobic atmosphere in a 20 mL reaction vial fitted with a pressure relief cap. The reaction mixture was sealed and heated to 213 °C for 6 h on a hot plate fitted with a Chemglass 4-place pie wedge for 20 mL scintillation vials. The crude reaction mixture was diluted with hexanes, wet loaded onto a silica column, and purified by normal phase flash chromatography. The product was eluted in a 1:1 solvent mixture of hexanes/CHCl_3_ and concentrated under reduced pressure to yield the isolated product as deep purple crystals (36 mg, 71%). X-ray quality crystals were grown by layering MeCN over the product dissolved in toluene. HRMS (MALDI) *m*/*z*: [M + H]^+^ calcd for C_120_H_125_ClFeN_4_Si_4_^+^ 1825.8047; found 1825.8016; [M – Cl]^+^ calcd for C_120_H_124_FeN_4_Si_4_^+^ 1789.8280; found 1789.8306; UV/vis (CHCl_3_) λ_abs_ (log ε): 379 (4.45), 442 (5.03), 520 (4.13), 588 (3.62), 707 (3.61). ^1^H NMR (500 MHz, CDCl_3_; paramagnetic) δ 81.06 (β-pyrrole); μ_eff_ (Evans, CDCl_3_): 6.59 μ_B_. HPLC (silica, hexane/DCM = ramp to 100% DCM, flow rate = 3.0 mL/min, λ = 440 nm) *t*_R_ = 9.2 min (98%).

## Supporting Information Available

Experimental details and spectra (PDF)
