# Supplementary material for: Synthesis and Functionalization of Challenging meso-Substituted Aryl Bis-pocket Porphyrins Accessed via Suzuki–Miyaura Cross-Coupling
Source: J Org Chem. 2022 Aug 17;87(17):11783–95. doi: 10.1021/acs.joc.2c01538 (PMC9447288; doi:10.1021/acs.joc.2c01538)
Supplement: Supplementary file 1 — jo2c01538_si_001.pdf [file jo2c01538_si_001.pdf]

Supporting Information for

Synthesis and Functionalization of Challenging *meso*-Substituted Aryl Bis-pocket Porphyrins Accessed via Suzuki-Miyaura Cross-Coupling

*Daniel G. Droege, A. Leila Parker, Griffin M. Milligan, Robert Jenkins, and Timothy C. Johnstone**

Department of Chemistry and Biochemistry, University of California, Santa Cruz, California 95064, United States.

* Correspondence to: johnstone@ucsc.edu

CONTENTS

	Page
Experimental methods	S3
Tables S1–S7: Crystallographic details	S5-S11
Tables S8: POVME2 pocket volumes	S12
NMR spectra	S13-S36
Figures S1-S4: HPLC chromatograms of **4b**, **4d**, **4e**, **4f**	S37-S38
Figures S5–S24: Thermal ellipsoid plots	S39-S58
References	S59

Experimental Methods

X-ray crystallography. Crystals of **2b**, **2c**, **2d**, **2e, 2f**, **2h**, **2i**, **2k**, **2l**, **2m**, **2o**, **2p**, **2q**, **3b**, **4a**, **4b**, **4c**, **4d**, **4e**, and **4f** were grown as described in the Experimental Section of the main text. Single crystals suitable for X-ray diffraction were selected under a microscope, loaded onto a nylon fiber loop using Paratone-N, and mounted onto a Rigaku XtaLAB Synergy-S single-crystal diffractometer. Each crystal was cooled to 100 K under a stream of nitrogen. Diffraction of Cu Kα radiation from a PhotonJet-S microfocus source was detected using a HyPix-6000HE hybrid photon counting detector. Screening, indexing, data collection, and data processing were performed with CrysAlis^{Pro}.[3] The structures were solved using SHELXT and refined using SHELXL as implemented in OLEX2 following established strategies.[4-7] Unless otherwise specified in the CIF, all non-H atoms were refined anisotropically and H atoms were placed at calculated positions and refined with a riding model and coupled isotropic displacement parameters. As noted in the appropriate CIFs, a number of the structures featured pockets of disordered solvent that could not be satisfactorily modeled. In these instances, the contribution of the electron density in those pockets to the observed structure factors was masked using Olex2. Refinement parameters are collected in Tables S1- S7.

Pocket volume estimation. Pocket volumes were calculated using POVME2.[1] PDB files of each porphyrin were generated from the corresponding X-ray diffraction coordinates. The grid spacing was set to 0.5 Å and a points-inclusion sphere of 10-Å radius was generated at the center of each porphyrin. A contiguous pocket-seed sphere of 4-Å radius was generated at the center of each porphyrin and a contiguous points criterion of 5 was employed (criteria of 3 and 7 were used for

2b and **2m**, respectively). Molecular graphics were generated with UCSF ChimeraX.[2] Pocket volumes are collected in Table S8.

Table S1. Crystallographic Refinement Details

**Compound**	**2b**·2MeCN	**2c**·MeCN·1.5CHCl$_3$	**2d**·MeCN·3C$_6$H$_3$Cl$_3$
Formula	$C_{108}H_{84}F_{16}N_6Si_4$	$C_{107.5}H_{82.5}C1_{20.5}N_5Si_4$	$C_{140}H_{138}Cl_9N_5Si_4$
FW	1882.17	2283.37	2321.96
T (K)	100.0(11)	99.99(10)	100.01(10)
λ (Å)	1.54184	1.54184	1.54184
Crystal System	Triclinic	Monoclinic	Monoclinic
Space group	$P\overline{1}$	*I*2/*a*	$P2_1/n$
a (Å)	12.5909(2)	23.2819(2)	16.53020(10)
b (Å)	13.6056(2)	19.67030(10)	40.0808(3)
c (Å)	17.3122(2)	47.5453(3)	19.68380(10)
α (°)	103.7060(10)	90	90
β (°)	106.6640(10)	92.3390(10)	97.4540(10)
γ (°)	106.8540(2)	90	90
Volume (Å^3)	2548.03(7)	21755.8(3)	12931.17(14)
Z	1	8	4
Size (mm^3)	0.16×0.07×0.04	0.41×0.31×0.03	0.16×0.11×0.06
θ range (°)	2.843-67.078	2.942-67.077	2.518-67.079
Total data	34597	138551	175824
Unique data	9086	19392	23102
Parameters	610	1307	1599
Completeness (%)	99.9	99.9	100.0
R_{int} (%)	2.90	4.61	4.19
R_1 (%, I > 2σ)	3.69	6.39	7.74
R_1 (%, all data)	4.14	6.90	8.05
wR_2 (%, I > 2σ)	9.60	18.20	20.51
wR_2 (%, all data)	9.87	18.72	20.70
S	1.028	1.033	1.158

Table S2. Crystallographic Refinement Details

**Compound**	**2e**·MeCN·C_7H_8	**2f**·2MeCN	**2h**·2MeCN·C_7H_5N
Formula	$C_{123}H_{124}N_6Si_4$	$C_{132}H_{148}N_6Si_4$	$C_{115}H_{97}F_8N_7Si_4$
FW	1798.63	1930.92	1841.35
T (K)	100.6(10)	100.00(11)	101(1)
λ (Å)	1.54184	1.54184	1.54184
Crystal System	Monoclinic	Triclinic	Monoclinic
Space group	$P2_1/c$	$P\overline{1}$	$P2_1/c$
a (Å)	16.34530(10)	13.3916(5)	17.9444(2)
b (Å)	29.9212(2)	15.4829(7)	14.42580(10)
c (Å)	23.1963(2)	16.1203(6)	23.4254(2)
α (°)	90	67.333(4)	90
β (°)	107.5830(10)	72.696(3)	110.6190(10)
γ (°)	90	87.191(3)	90
Volume ($Å^3$)	10814.62(15)	2936.5(2)	5675.51(10)
Z	4	1	2
Size (mm^3)	0.13×0.08×0.07	0.88×0.18×0.05	0.29×0.17×0.05
θ range (°)	2.485-67.079	3.117-67.067	2.631-67.078
Total data	171800	38820	106189
Unique data	19313	10456	10128
Parameters	1281	732	646
Completeness (%)	100	99.9	99.9
R_{int} (%)	3.28	5.87	3.90
R_1 (%, I > 2σ)	4.51	5.63	5.65
R_1 (%, all data)	4.79	6.59	5.86
wR_2 (%, I > 2σ)	11.01	15.16	15.41
wR_2 (%, all data)	11.16	15.97	15.57
S	1.114	1.033	1.047

Table S3. Crystallographic Refinement Details

**Compound**	**2i**·$CHCl_3$	**2k**·2MeCN·$CHCl_3$	**2l**·2MeCN
Formula	$C_{113}H_{87}Cl_3F_{24}N_4Si_4$	$C_{117}H_{117}Cl_3N_6O_8Si_4$	$C_{140}H_{116}N_6Si_4$
FW	2175.57	1953.87	1994.74
T (K)	99.99(10)	100.0(13)	99.98(15)
λ (Å)	1.54184	1.54184	1.54184
Crystal System	Monoclinic	Triclinic	Monoclinic
Space group	$P2_1/n$	$P\bar{1}$	$P2_1/c$
a (Å)	18.7167(2)	11.9189(2)	16.6762(3)
b (Å)	23.8213(2)	15.7967(3)	23.8081(4)
c (Å)	26.6679(3)	16.5347(3)	30.0004(3)
α (°)	90	101.9810(10)	90
β (°)	93.2670(10)	108.897(2)	91.4430(10)
γ (°)	90	107.1330(10)	90
Volume ($Å^3$)	11870.7(2)	2651.88(9)	11907.2(3)
Z	4	1	4
Size (mm^3)	0.22×0.1×0.06	0.23×0.09×0.06	0.23×0.15×0.03
θ range (°)	2.489-67.077	2.998-67.078	2.369-67.078
Total data	167793	35890	95662
Unique data	21182	9457	21185
Parameters	1510	651	1598
Completeness (%)	100.0	99.9	99.6
R_{int} (%)	4.76	3.42	3.62
R_1 (%, I > 2σ)	6.51	6.01	6.76
R_1 (%, all data)	7.53	6.66	8.32
wR_2 (%, I > 2σ)	16.97	16.44	18.24
wR_2 (%, all data)	17.69	16.97	19.37
S	1.029	1.042	1.022

Table S4. Crystallographic Refinement Details

**Compound**	**2m**	**2o**·5MeCN	**2p**
Formula	$C_{80}H_{94}N_4Si_4$	$C_{138}H_{173}N_9Si_{12}$	$C_{128}H_{126}N_4O_{16}Si_4$
FW	1223.95	2294.92	2088.68
T (K)	99.99(14)	100.01(10)	100.01(10)
λ (Å)	1.54184	1.54184	1.54184
Crystal System	Triclinic	Monoclinic	Monoclinic
Space group	$P\overline{1}$	$P2_1/n$	$I2/a$
a (Å)	14.3294(2)	23.9843(2)	22.8311(2)
b (Å)	16.7136(3)	24.2555(2)	24.7546(3)
c (Å)	18.2148(2)	27.5665(2)	23.6019(4)
α (°)	103.3400(10)	90	90
β (°)	90.2160(10)	90.8630(10)	98.1630(10)
γ (°)	100.0870(10)	90	90
Volume ($Å^3$)	4174.61(11)	16035.0(2)	13204.0(3)
Z	2	4	4
Size (mm^3)	0.29×0.08×0.03	0.81×0.6×0.27	0.29×0.25×0.18
θ range (°)	2.496-67.075	2.424-67.080	2.601-67.073
Total data	56459	218940	11799
Unique data	14868	28618	11799
Parameters	897	1681	878
Completeness (%)	99.8	99.9	100.0
R_{int} (%)	4.38	5.03	N/A[a]
R_1 (%, I > 2σ)	8.19	6.82	10.13
R_1 (%, all data)	8.84	7.44	10.63
wR_2 (%, I > 2σ)	20.86	18.58	30.79
wR_2 (%, all data)	21.25	19.11	31.29
S	1.091	1.024	1.049

[a] Non-merohedral twin

Table S5. Crystallographic Refinement Details

**Compound**	**2q**·½Et_2O	**3b**·5$C_6H_{14}O_3$	**4a**·MeCN·H_2O
Formula	$C_{90}H_{99}N_{20}O_{0.50}Si_4$	$C_{122}H_{112}F_{16}N_4Na_4O_{27}S_4$	$C_{106}H_{97}N_5OSi_4Zn$
FW	1581.25	2590.35	1634.61
T (K)	100.0(3)	100.01(11)	100.0(10)
λ (Å)	1.54184	1.54184	1.54184
Crystal System	Triclinic	Monoclinic	Triclinic
Space group	$P\overline{1}$	$C2/m$	$P\overline{1}$
a (Å)	15.5214(3)	13.5375(2)	13.1539(2)
b (Å)	16.8377(3)	32.1685(4)	13.6553(2)
c (Å)	19.6805(3)	14.4984(2)	16.1176(3)
α (°)	104.498(2)	90	73.1390(10)
β (°)	91.652(2)	90.9620(10)	70.611(2)
γ (°)	92.538(2)	90	61.251(2)
Volume ($Å^3$)	4970.49	6312.89(15)	2364.30(8)
Z	2	2	1
Size (mm^3)	0.27×0.05×0.04	0.15×0.08×0.07	0.1×0.06×0.03
θ range (°)	2.321-67.077	2.747-67.067	2.943-67.078
Total data	64662	39300	69421
Unique data	17671	5750	8457
Parameters	1121	537	552
Completeness (%)	99.5	100.0	100
R_{int} (%)	6.26	4.87	3.58
R_1 (%, I > 2σ)	6.63	4.88	5.65
R_1 (%, all data)	7.46	5.22	5.95
wR_2 (%, I > 2σ)	18.38	13.17	14.83
wR_2 (%, all data)	19.22	13.43	15.04
S	1.041	1.050	1.115

Table S6. Crystallographic Refinement Details

**Compound**	**4b**·2MeCN	**4c**·2MeCN	**4d**·2MeCN
Formula	$C_{108}H_{98}CuN_6Si_4$	$C_{108}H_{98}N_6PdSi_4$	$C_{108}H_{98}CoN_6Si_4$
FW	1655.82	1698.68	1651.21
T (K)	100.0(10)	99.9(2)	101(2)
λ (Å)	1.54184	1.54184	1.54184
Crystal System	Triclinic	Triclinic	Triclinic
Space group	$P\overline{1}$	$P\overline{1}$	$P\overline{1}$
a (Å)	13.1905(2)	13.2012(3)	13.1816(2)
b (Å)	13.6409(3)	13.6615(3)	13.6278(2)
c (Å)	16.0599(2)	16.0477(3)	16.0608(2)
α (°)	72.320(2)	72.357(2)	72.3800(10)
β (°)	71.031(2)	70.882(2)	70.9320(10)
γ (°)	61.419(2)	61.519(2)	61.282(2)
Volume ($Å^3$)	2361.21(9)	2365.70(10)	2354.14(7)
Z	1	1	1
Size (mm^3)	0.15×0.07×0.02	0.42×0.16×0.04	0.1×0.08×0.03
θ range (°)	2.957-67.073	2.961-67.080	2.957-67.076
Total data	66105	63611	31966
Unique data	8428	8428	8410
Parameters	545	545	545
Completeness (%)	99.9	99.6	99.9
R_{int} (%)	4.61	5.48	3.01
R_1 (%, I > 2σ)	3.60	3.87	3.30
R_1 (%, all data)	4.14	4.05	3.42
wR_2 (%, I > 2σ)	9.38	10.30	7.76
wR_2 (%, all data)	9.70	10.45	7.82
S	1.035	1.047	1.023

Table S7. Crystallographic Refinement Details

**Compound**	**4e**·MeCN·$CHCl_3$	**4f**·½C_7H_8
Formula	$C_{107}H_{96}Cl_4FeN_5Si_4$	$C_{123.5}H_{128}ClFeN_4Si_4$
FW	1761.89	1871.95
T (K)	100.0(10)	100.0(12)
λ (Å)	1.54184	1.54184
Crystal System	Triclinic	Monoclinic
Space group	$P\overline{1}$	$P2_1/n$
a (Å)	12.8777(3)	13.85030(10)
b (Å)	16.3497(4)	31.0859(3)
c (Å)	23.5222(4)	26.2645(3)
α (°)	106.739(2)	90
β (°)	90.100(2)	90.4670(10)
γ (°)	90.228(2)	90
Volume ($Å^3$)	4742.60(19)	11307.78(19)
Z	2	4
Size (mm^3)	0.21×0.09×0.05	0.07×0.05×0.03
θ range (°)	2.822-67.081	2.843-67.071
Total data	61182	143167
Unique data	16845	20048
Parameters	1158	1520
Completeness (%)	99.4	99.2
R_{int} (%)	4.20	5.84
R_1 (%, I > 2σ)	11.82	7.60
R_1 (%, all data)	12.45	9.08
wR_2 (%, I > 2σ)	26.22	18.59
wR_2 (%, all data)	26.51	19.27
S	1.222	1.126

Table S8. Pocket Volumes

Compound	Pocket Volume A^3 ([a])	Pocket Volume (top, bottom) A^3
2a	22 (11)	
2b	23.25(12)	
2c	34.75	(28.5, 6.25)
2d	12.5	(9, 3.5)
2e	19.25(10)	
2f	34.75(17.3)	
2h	25.0(12.5)	
2i	13.0	(7.625,5.375)
2k	31.75(16)	
2l	20.875(11)	
2m	44.5(22)	
2o	23.875(12.1)	
2p	16.25	(10.75,5.5)
2q	10.25	Only top pocket
3b	7.75 (3.875)	

[a] average volume across both pockets.

NMR Spectra: Scope

2b ^{1}H NMR (500 MHz, $CDCl_3$)

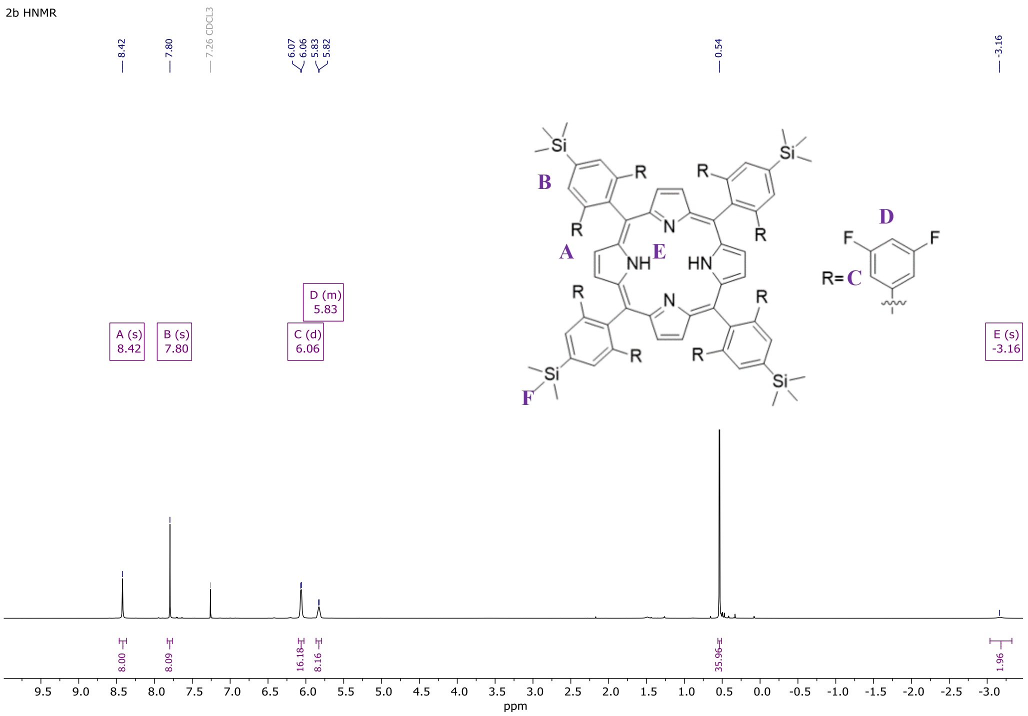

2b ^{19}F{^{1}H} NMR (470 MHz, $CDCl_3$)

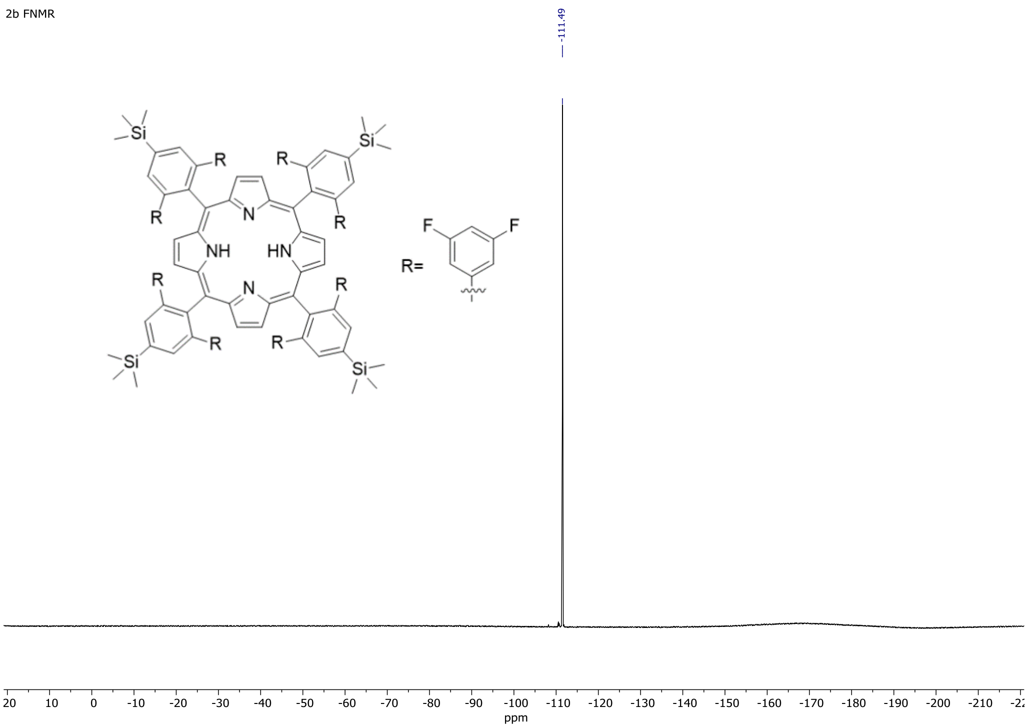

2b $^{13}C\{^{1}H\}$ NMR (126 MHz, $CDCl_3$)

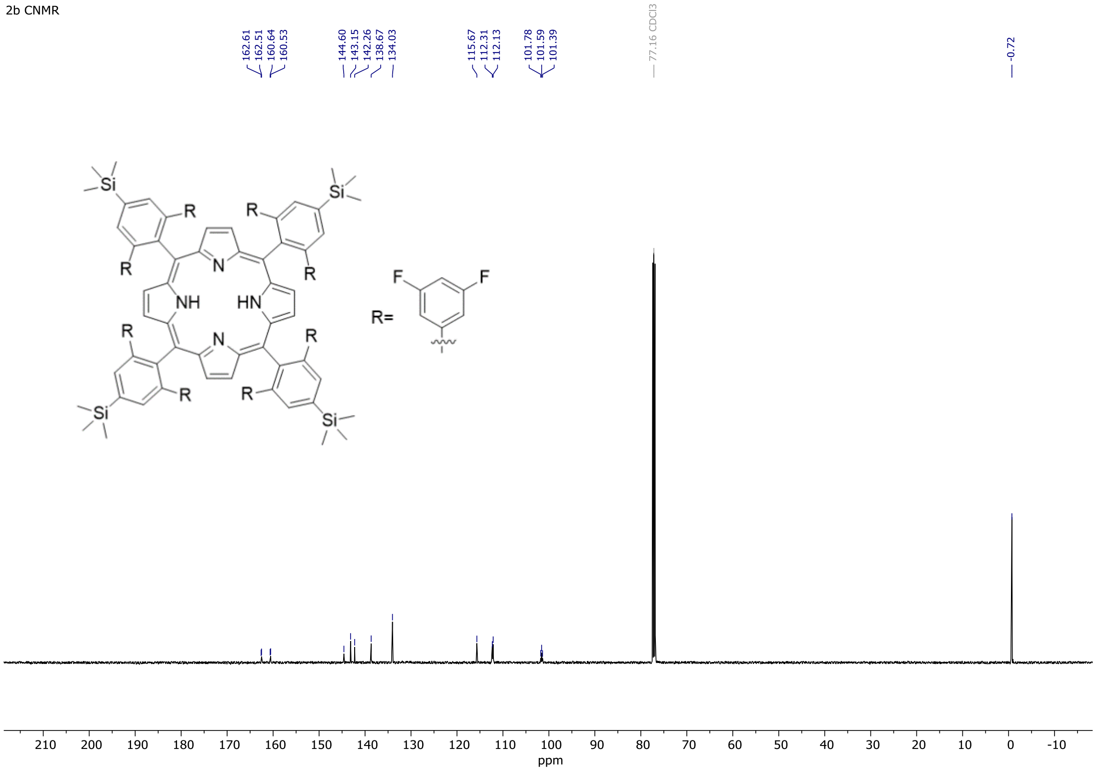

2c ^{1}H NMR (500 MHz, $CDCl_3$)

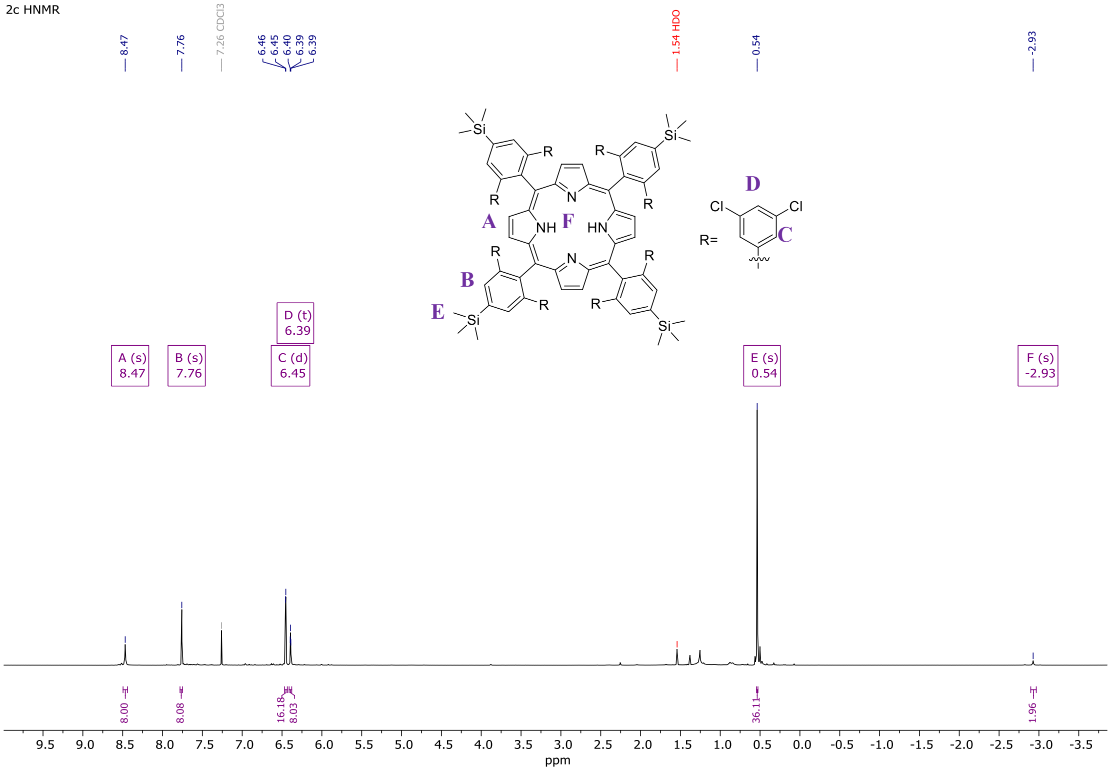

2c $^{13}C\{^1H\}$ NMR (126 MHz, $CDCl_3$)

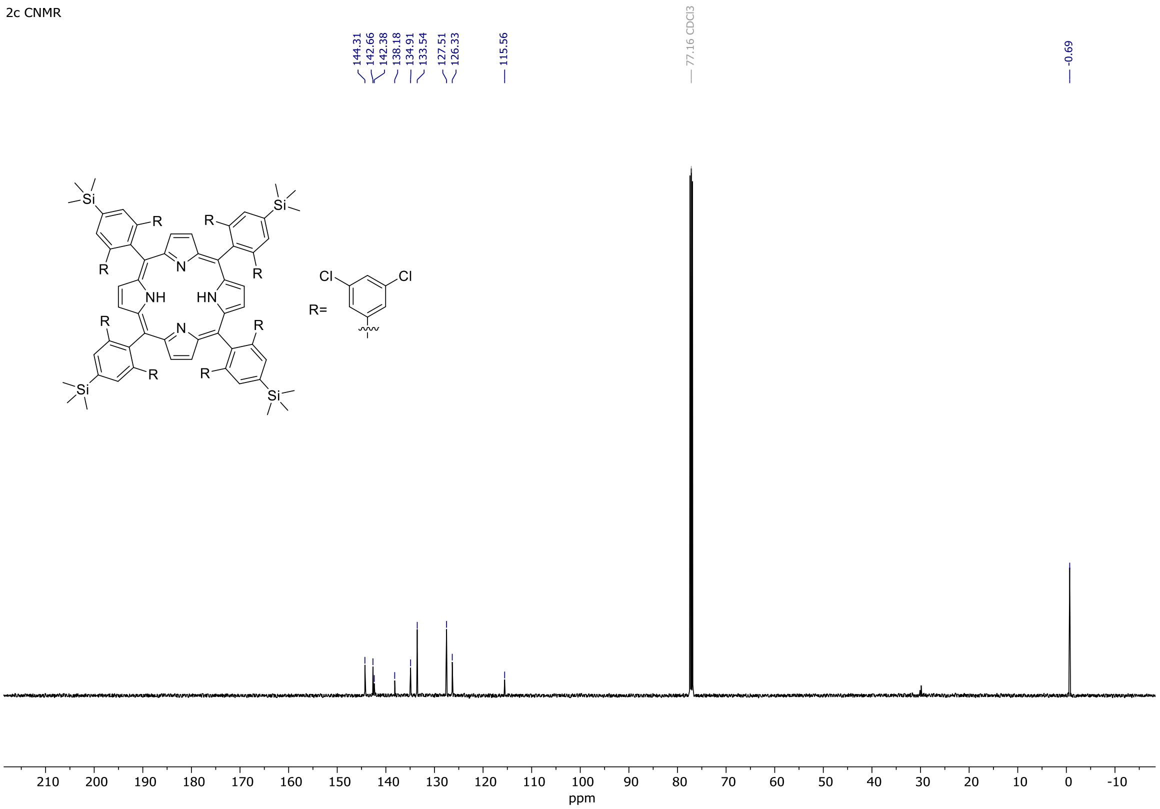

2d ^1H NMR (500 MHz, $CDCl_3$)

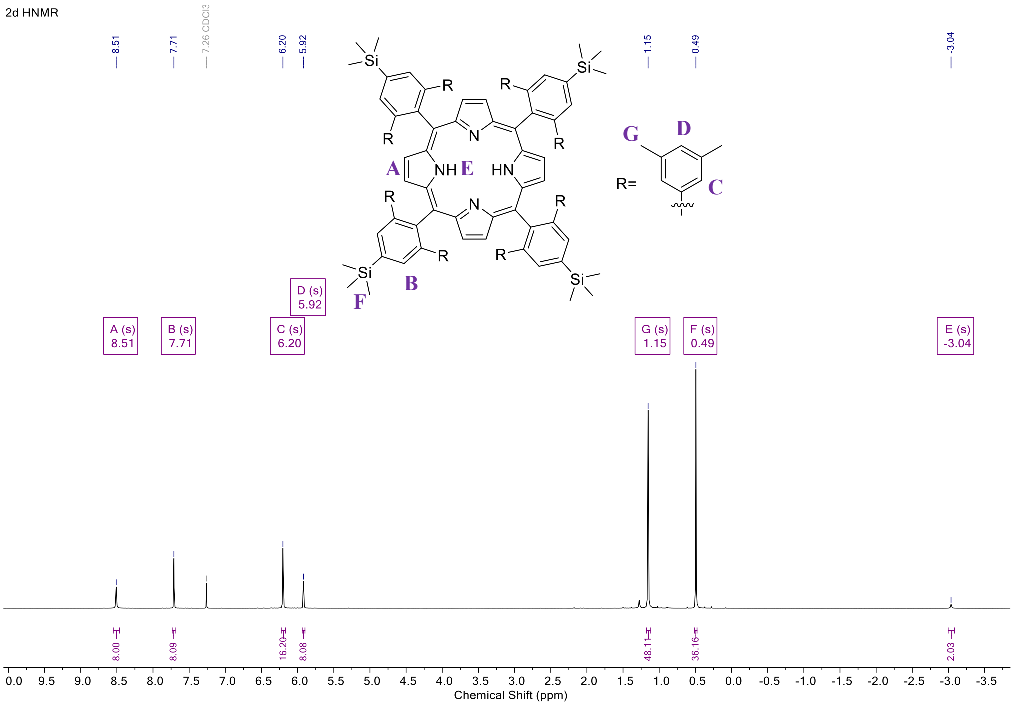

2d $^{13}C\{^1H\}$ NMR (126 MHz, $CDCl_3$)

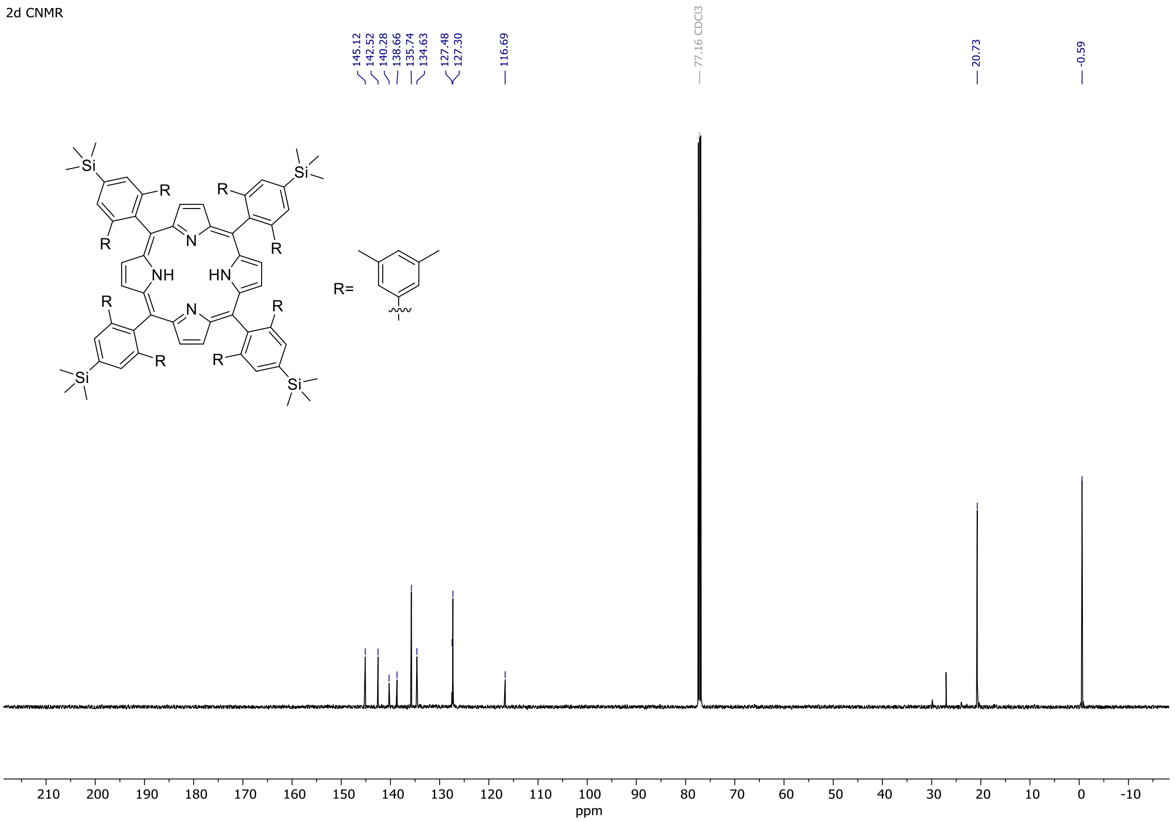

2e ^1H NMR (500 MHz, $CDCl_3$)

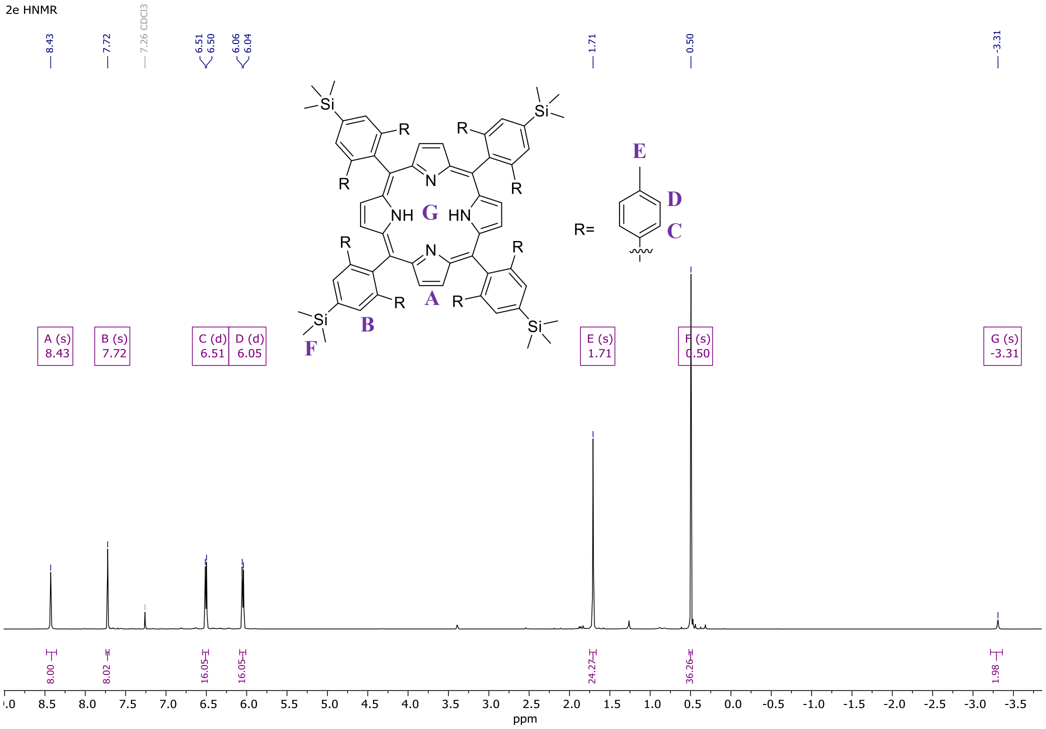

2e $^{13}C\{^{1}H\}$ NMR (126 MHz, $CDCl_3$)

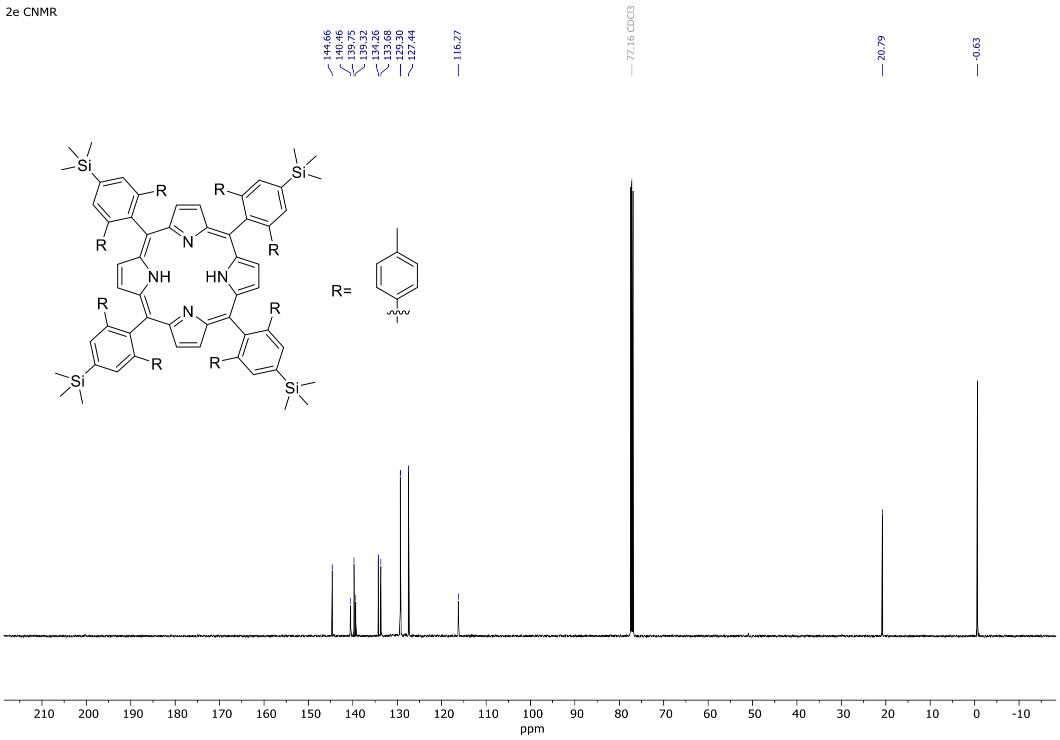

2f ^{1}H NMR (500 MHz, $CDCl_3$)

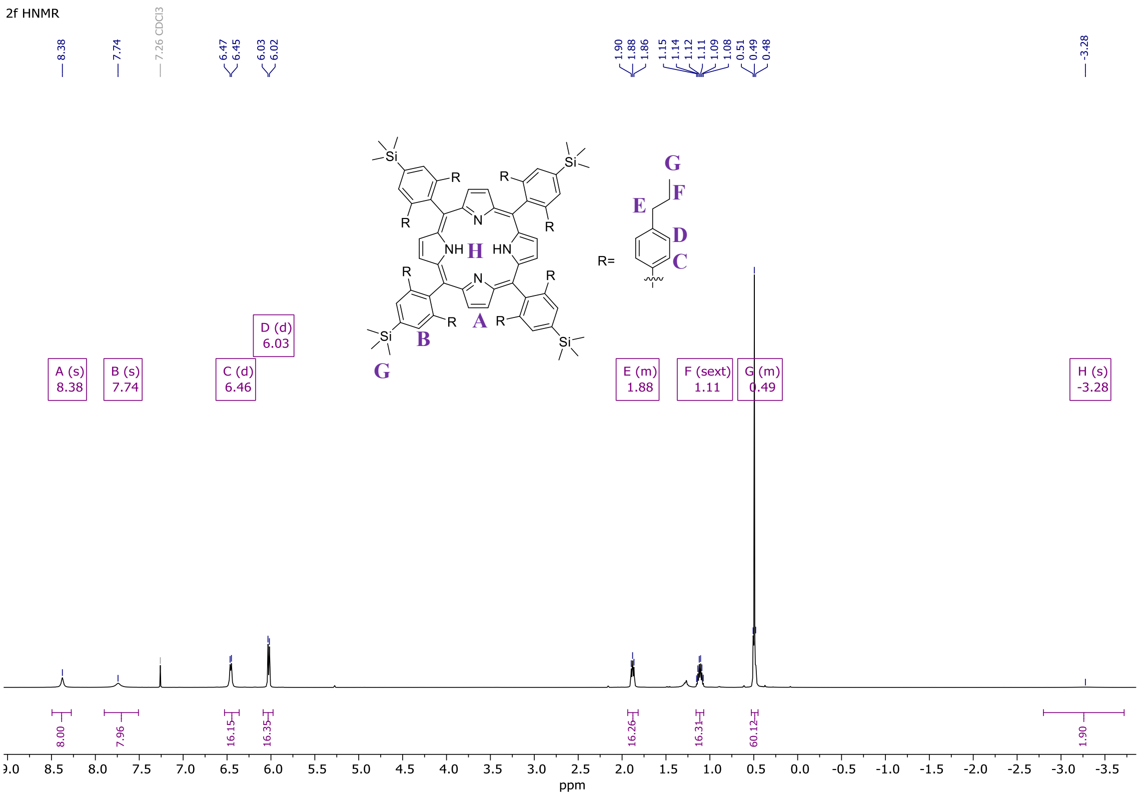

2f $^{13}C\{^{1}H\}$ NMR (126 MHz, $CDCl_3$)

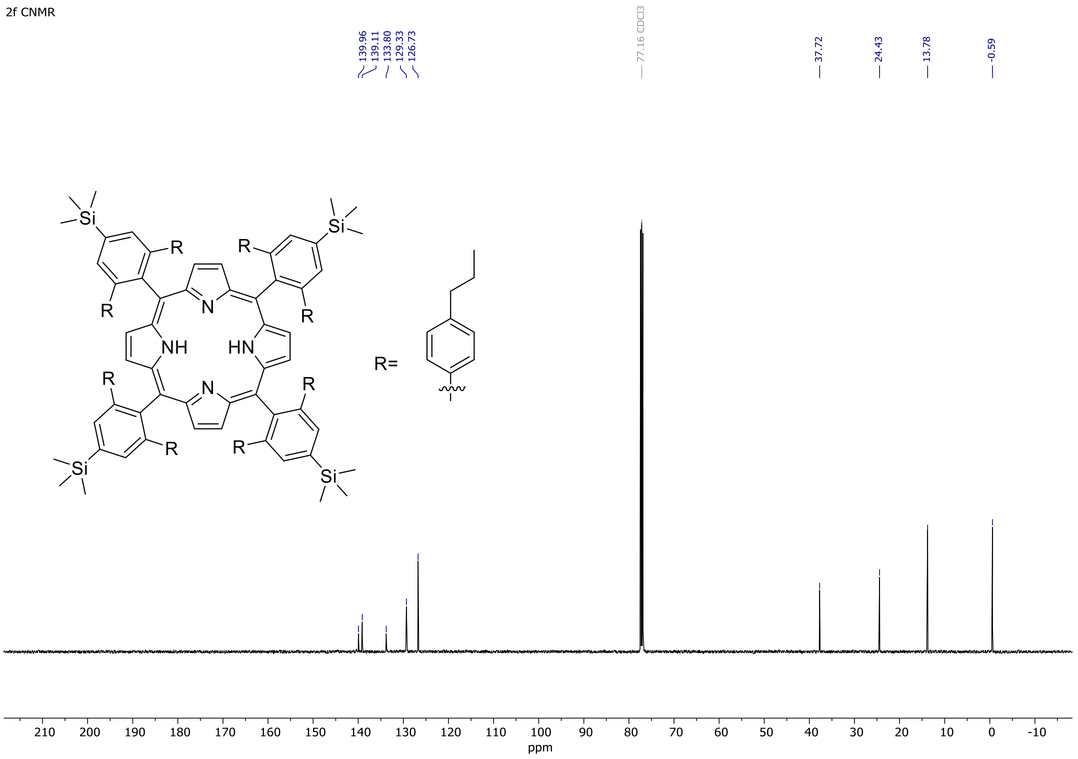

2g ^{1}H NMR (500 MHz, $CDCl_3$)

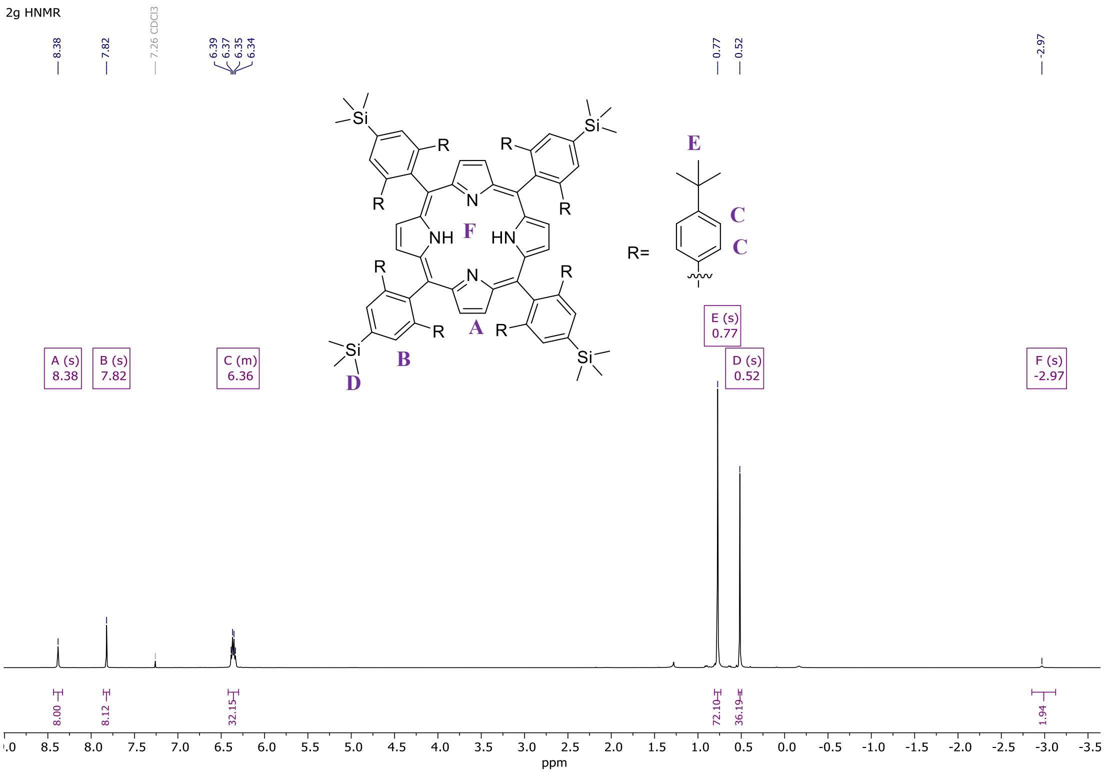

2g $^{13}C\{^1H\}$ NMR (126 MHz, $CDCl_3$)

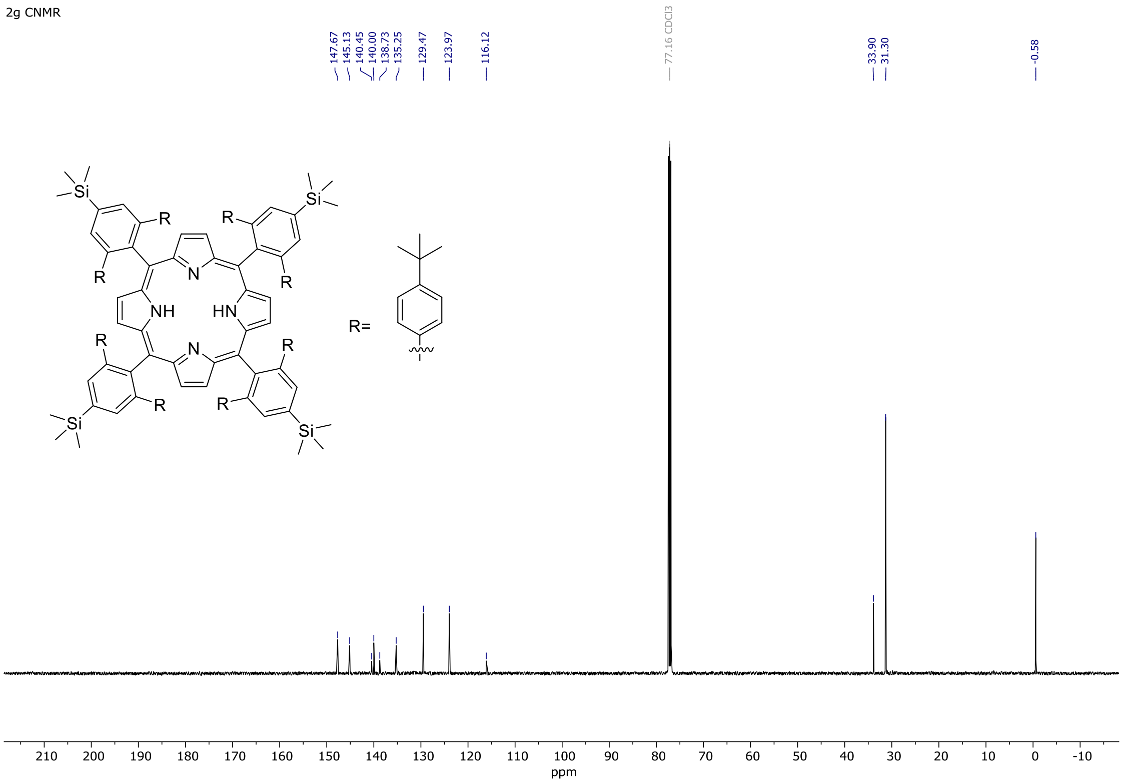

2h ^1H NMR (500 MHz, $CDCl_3$)

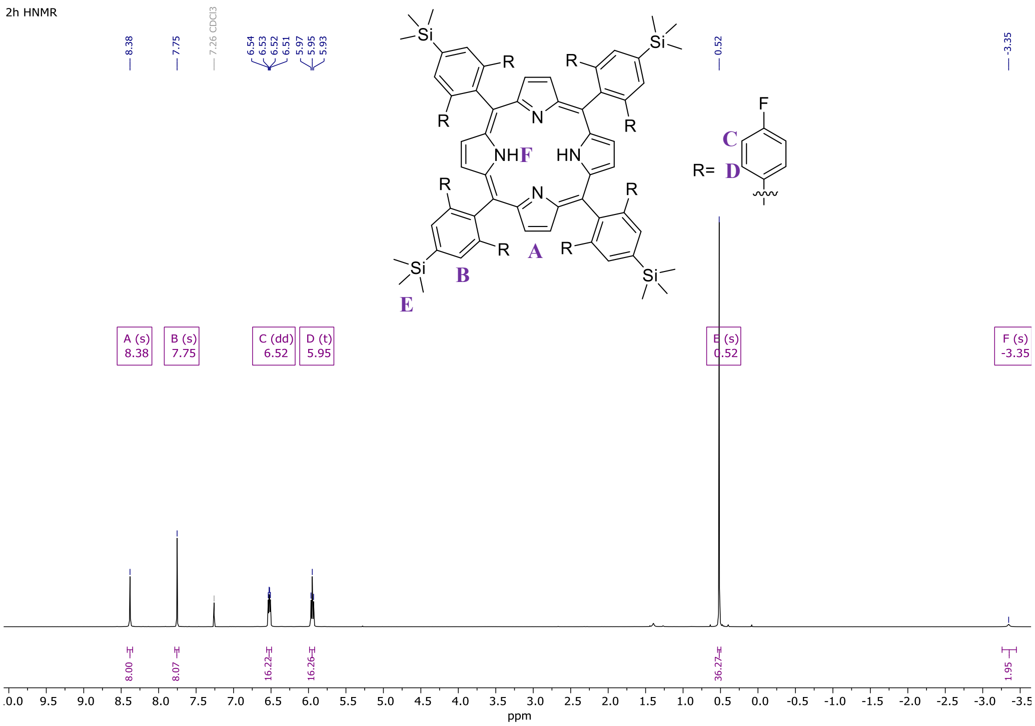

2h $^{13}C\{^1H\}$ NMR (126 MHz, $CDCl_3$)

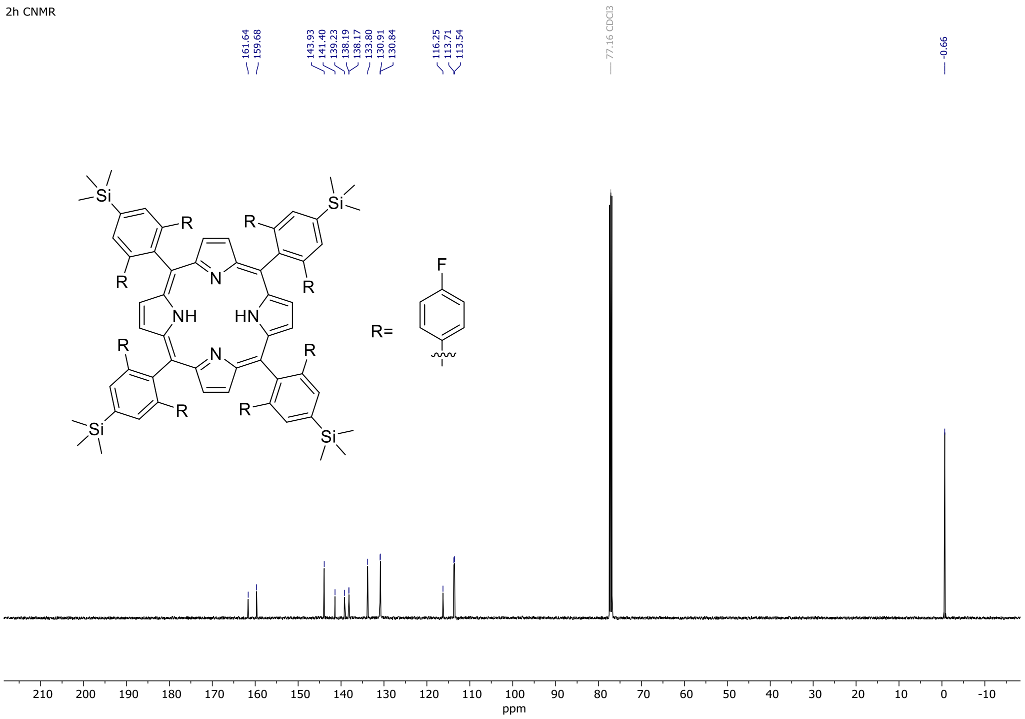

2h $^{19}F\{^1H\}$ NMR (470 MHz, $CDCl_3$)

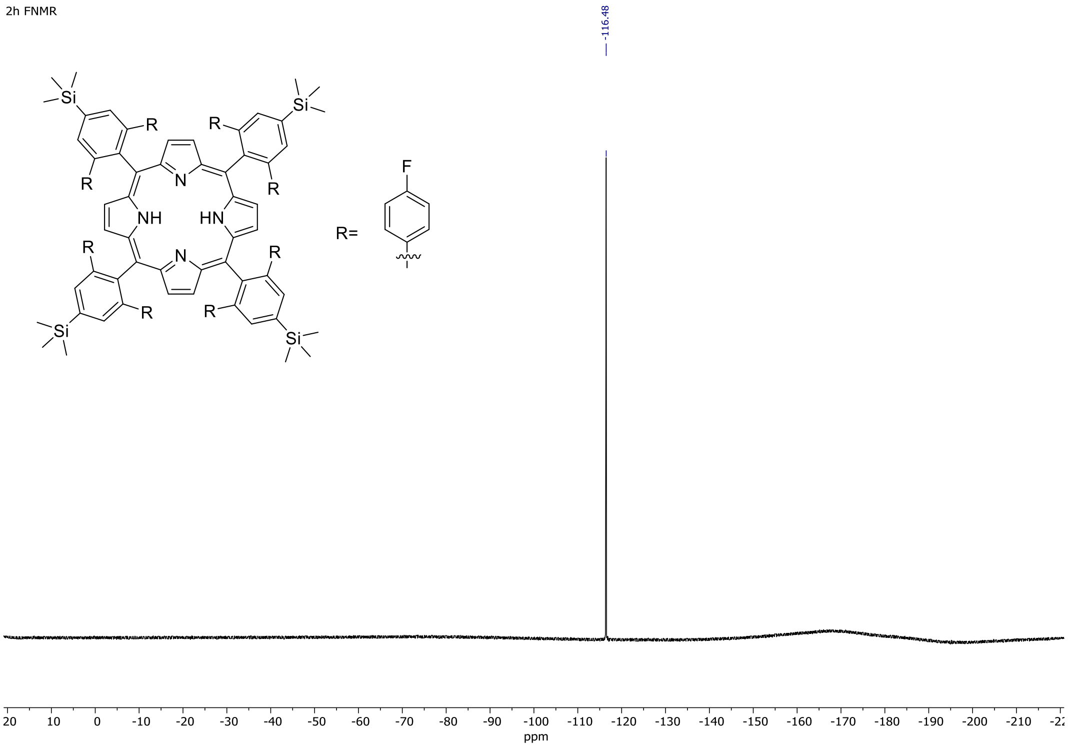

2i ^{1}H NMR (500 MHz, $CDCl_3$)

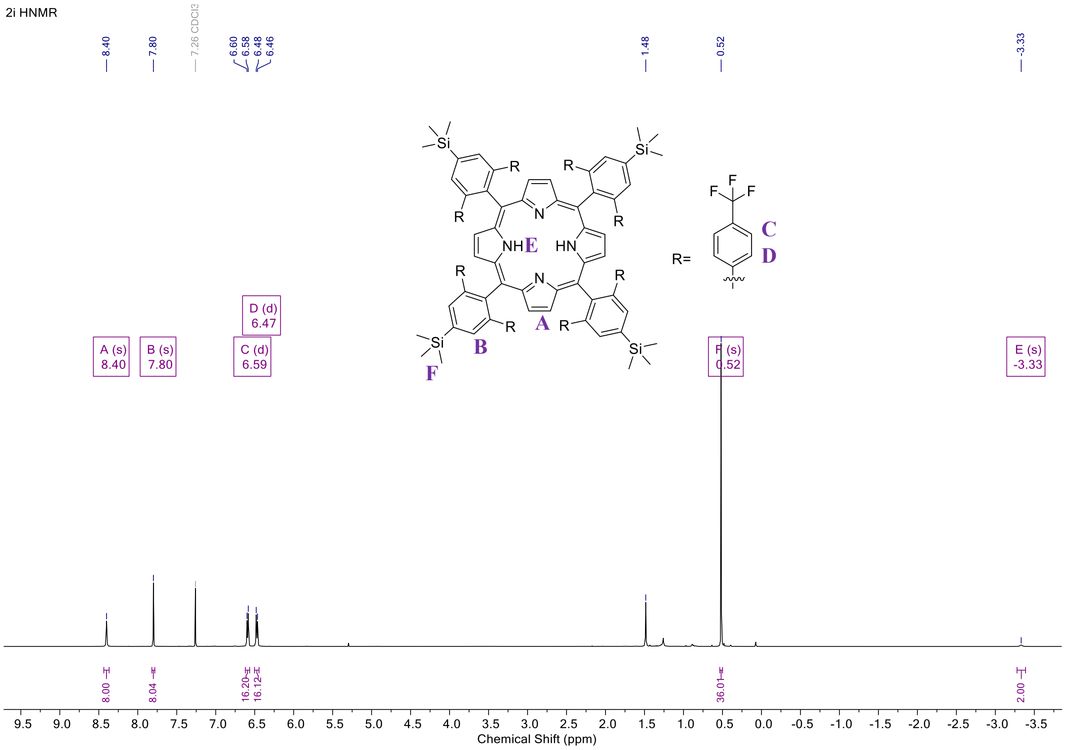

2i ^{19}F{^{1}H} NMR (470 MHz, $CDCl_3$)

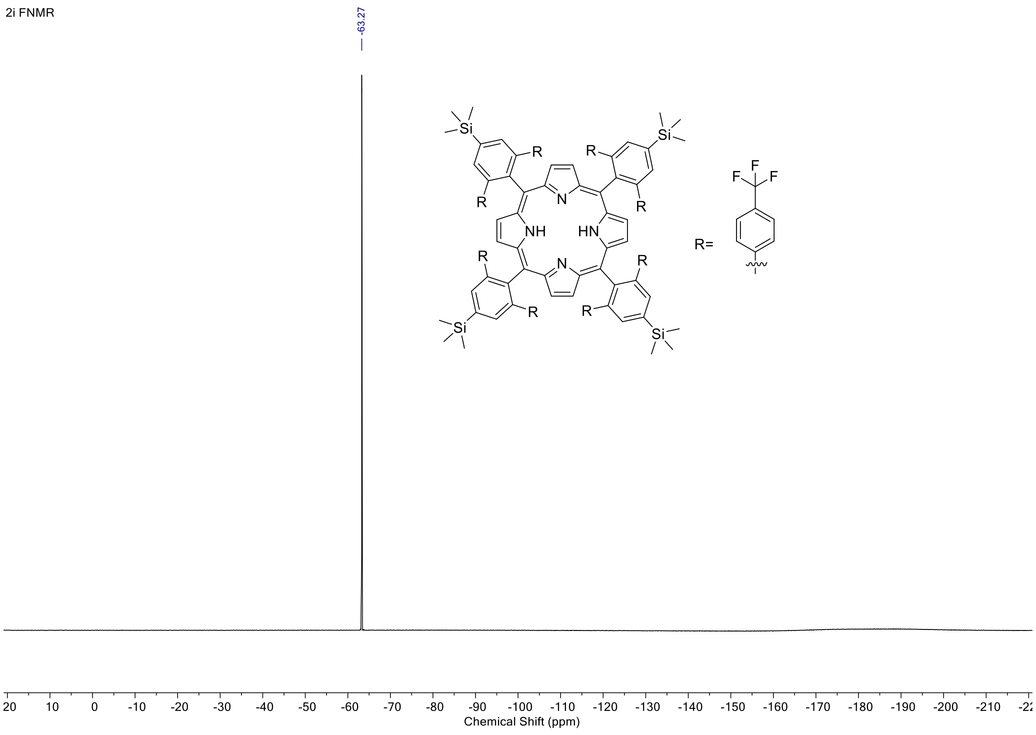

2i $^{13}C\{^1H\}$ NMR (126 MHz, $CDCl_3$)

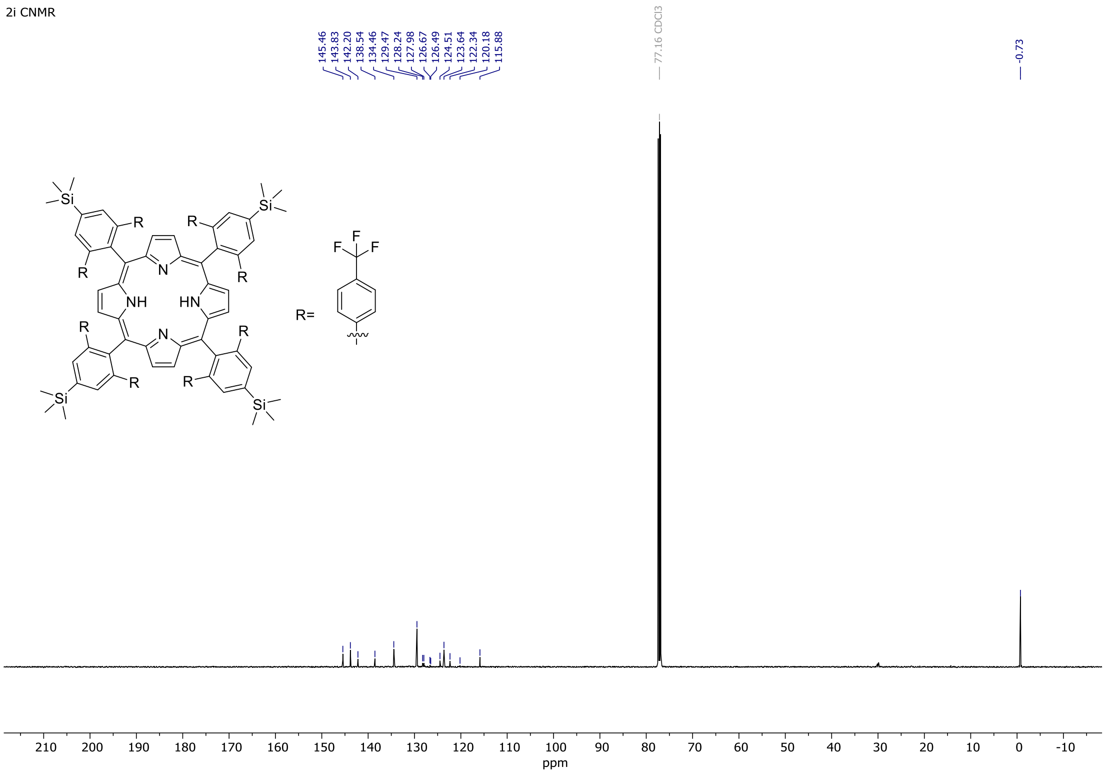

2j ^1H NMR (500 MHz, $CDCl_3$)

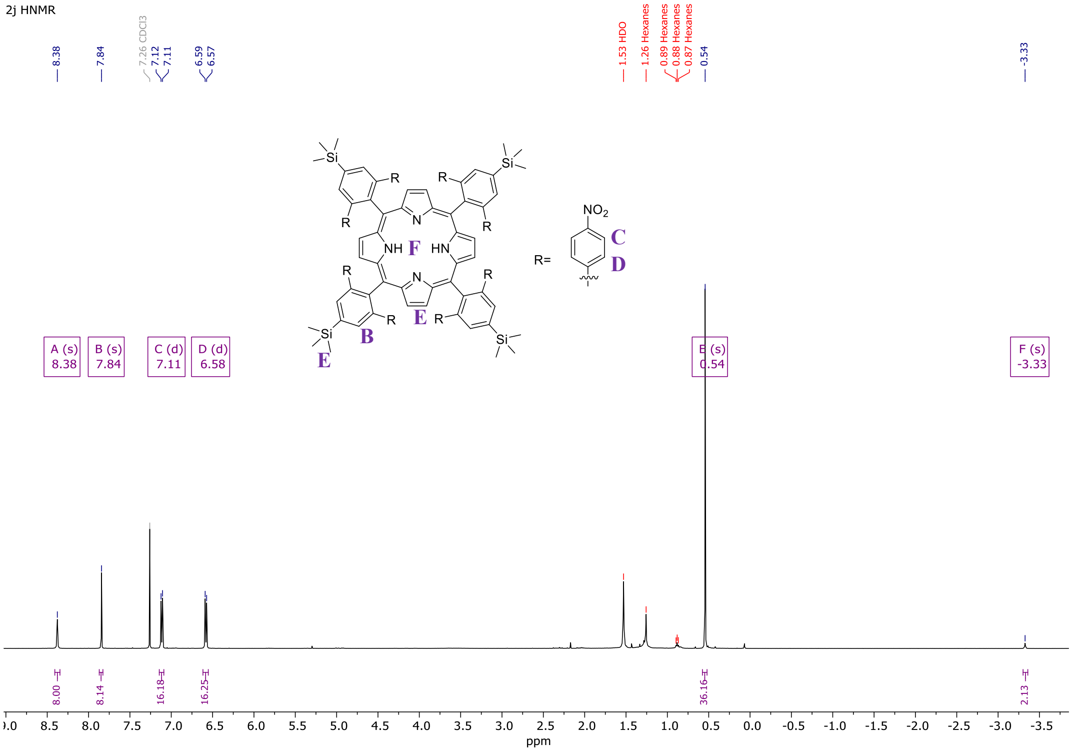

2j $^{13}C\{^{1}H\}$ NMR (126 MHz, $CDCl_3$)

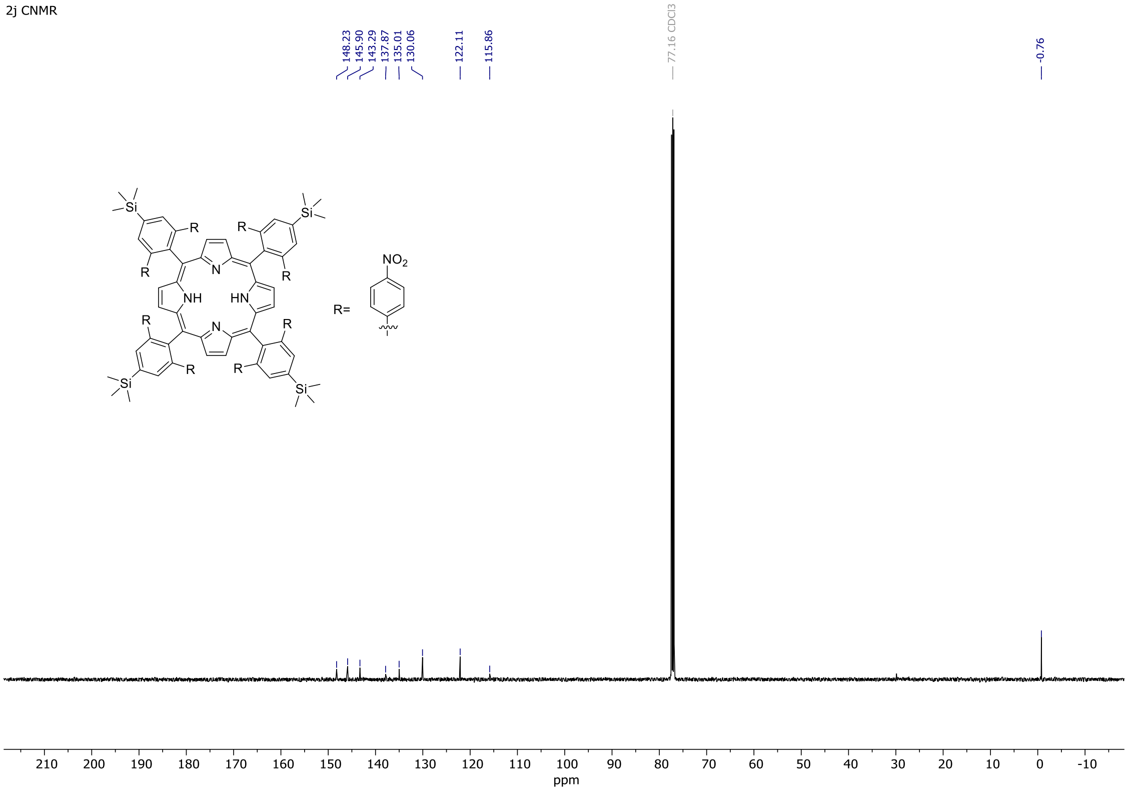

2k ^{1}H NMR (500 MHz, $CDCl_3$)

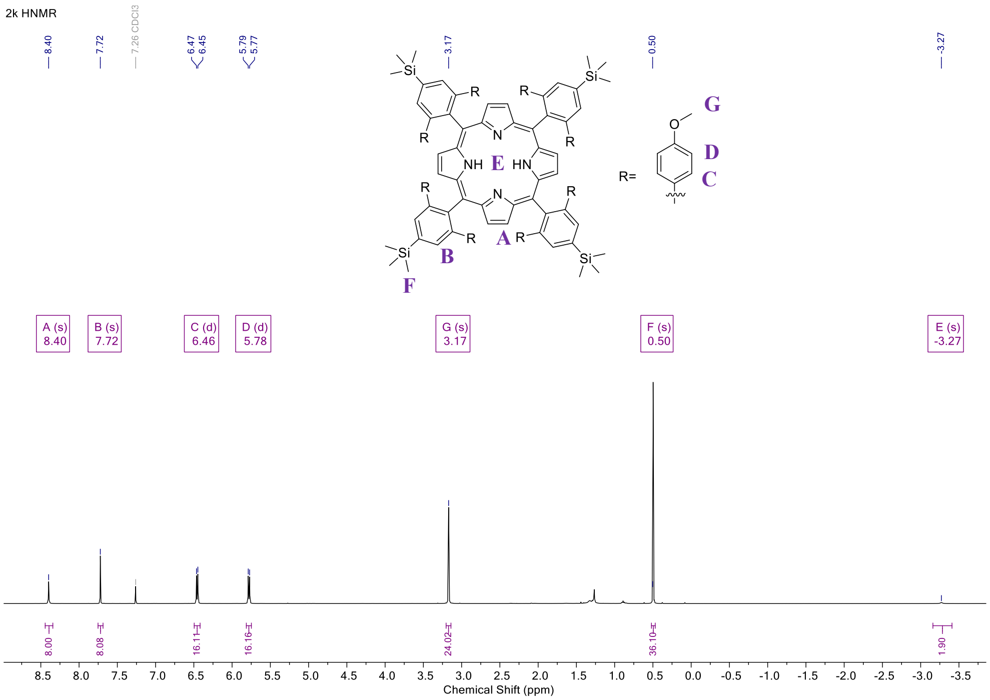

2k $^{13}C\{^{1}H\}$ NMR (126 MHz, $CDCl_3$)

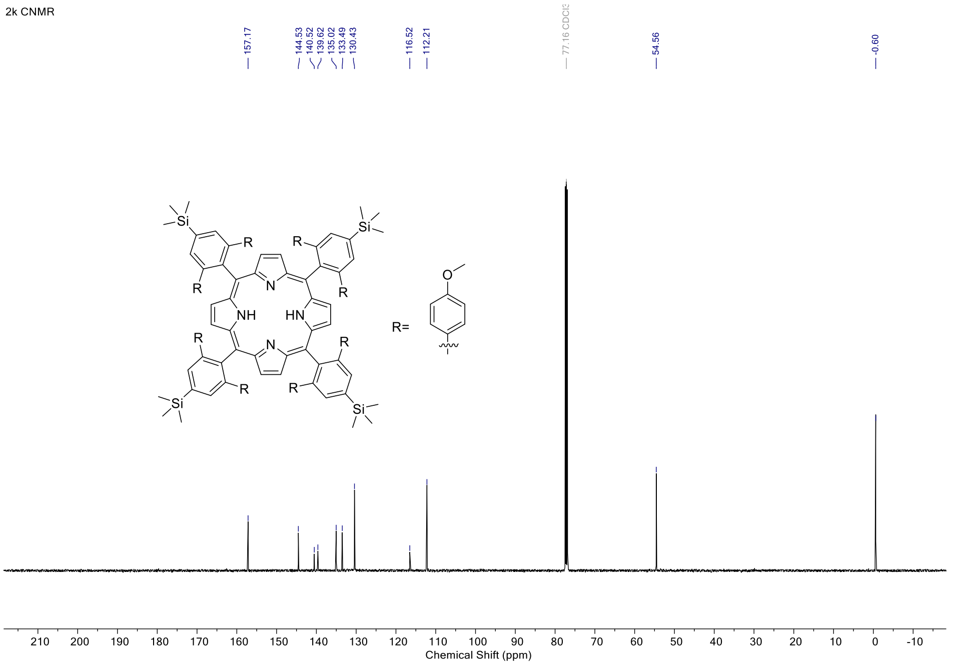

2l ^{1}H NMR (500 MHz, $CDCl_3$) (* tentative assignment)

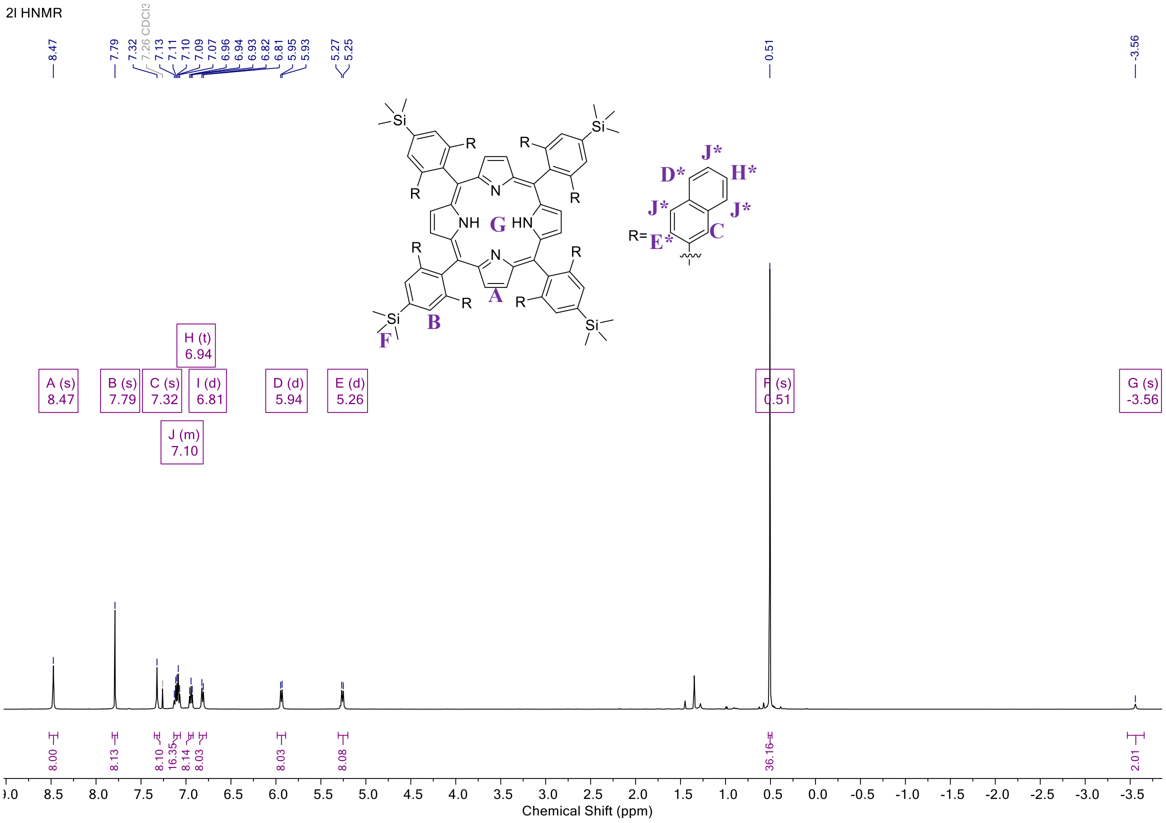

2l $^{13}C\{^{1}H\}$ NMR (126 MHz, $CDCl_3$)

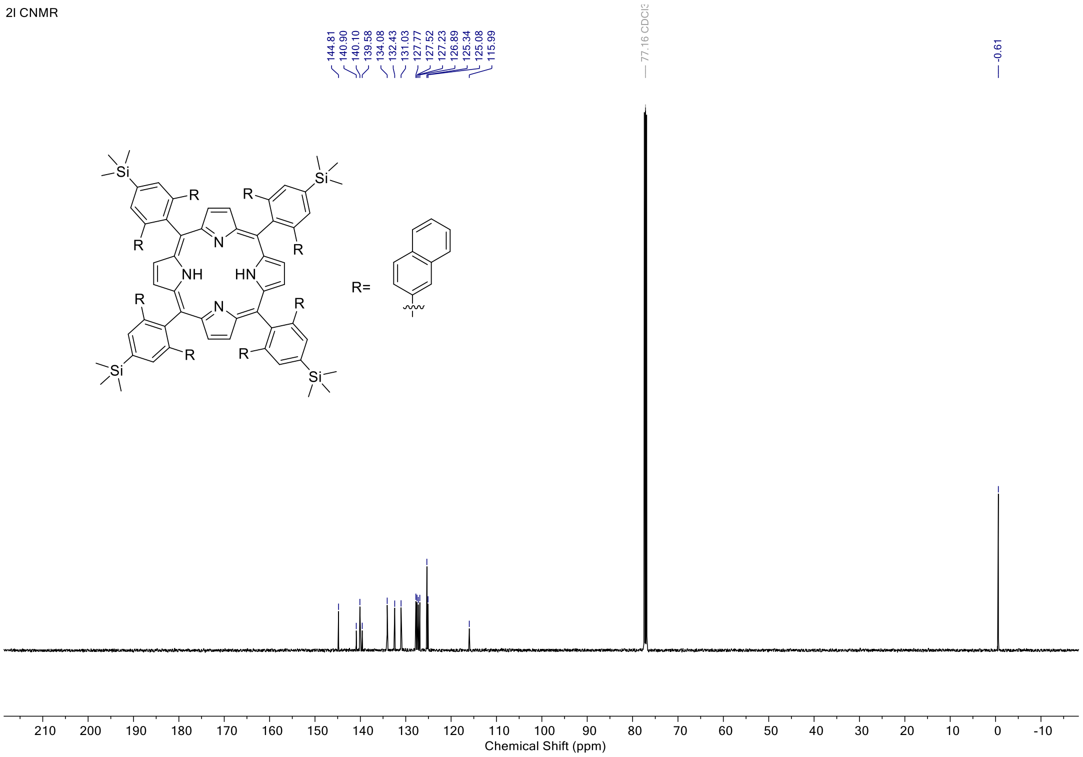

2m ^{1}H NMR (500 MHz, $CDCl_3$)

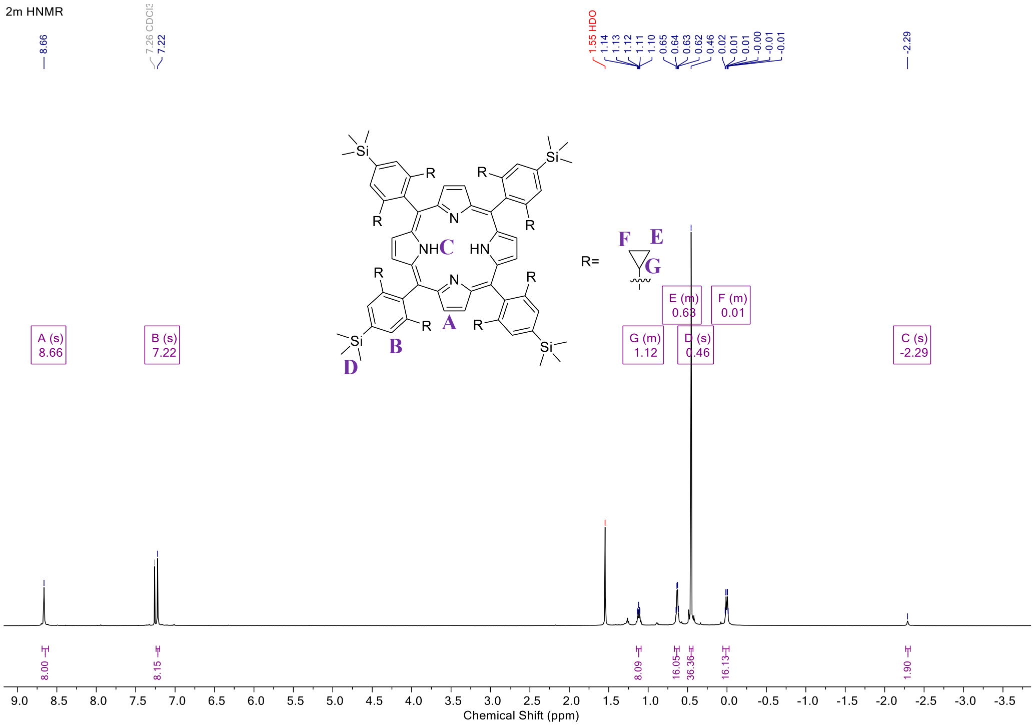

2m $^{13}C\{^{1}H\}$ NMR (126 MHz, $CDCl_3$)

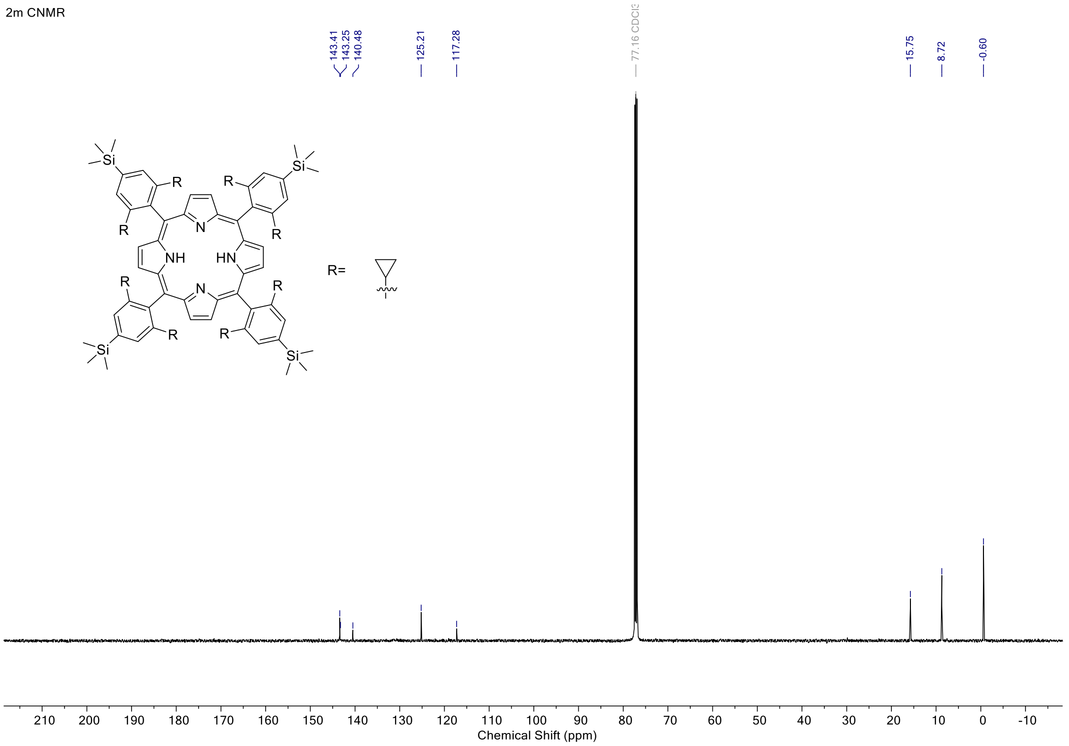

2n ^{1}H NMR (500 MHz, $CDCl_3$)

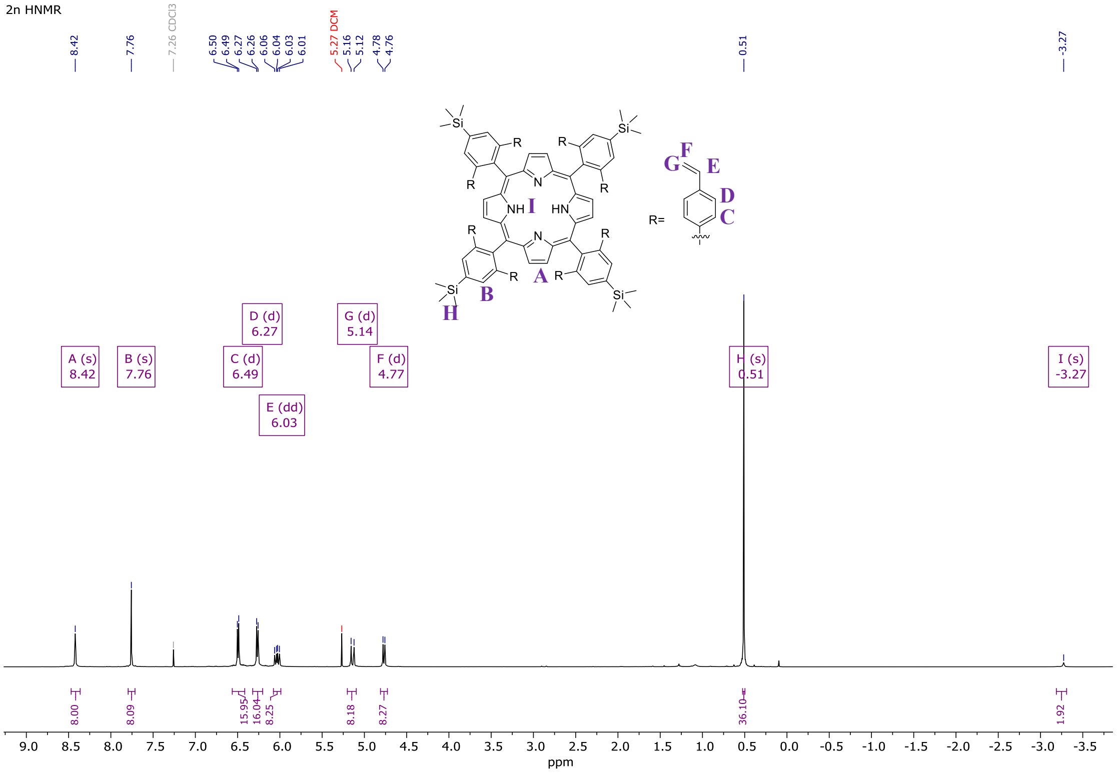

2n $^{13}C\{^1H\}$ NMR (126 MHz, $CDCl_3$)

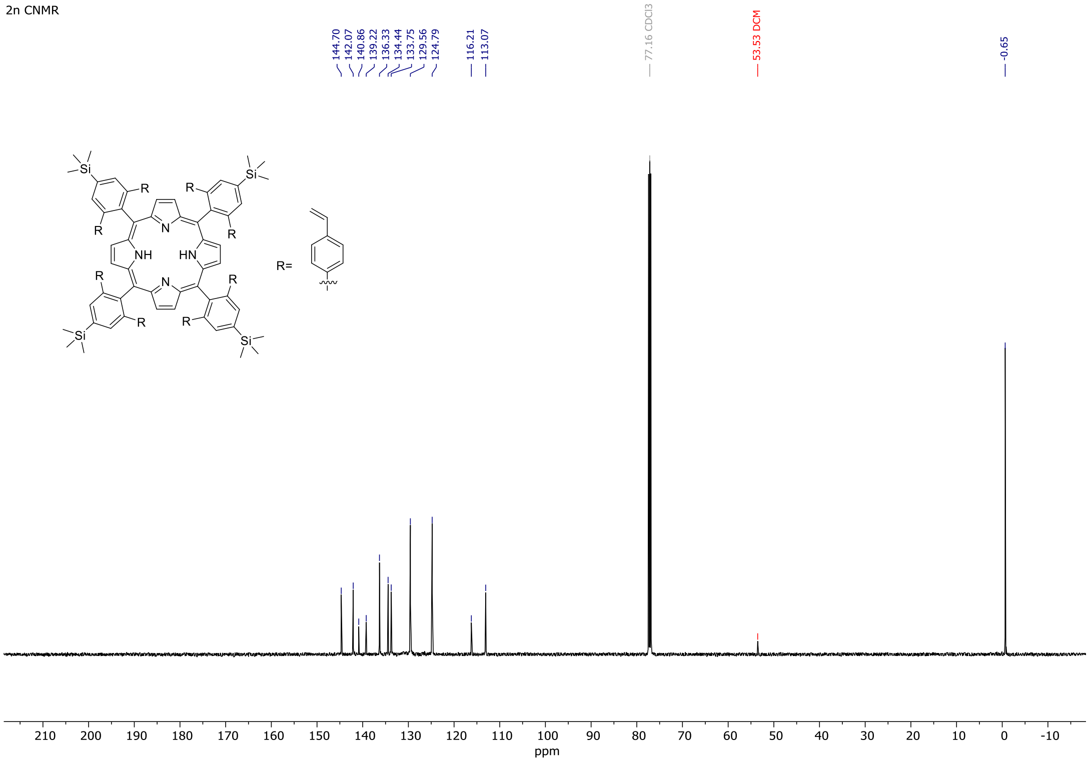

2o ^1H NMR (500 MHz, $CDCl_3$)

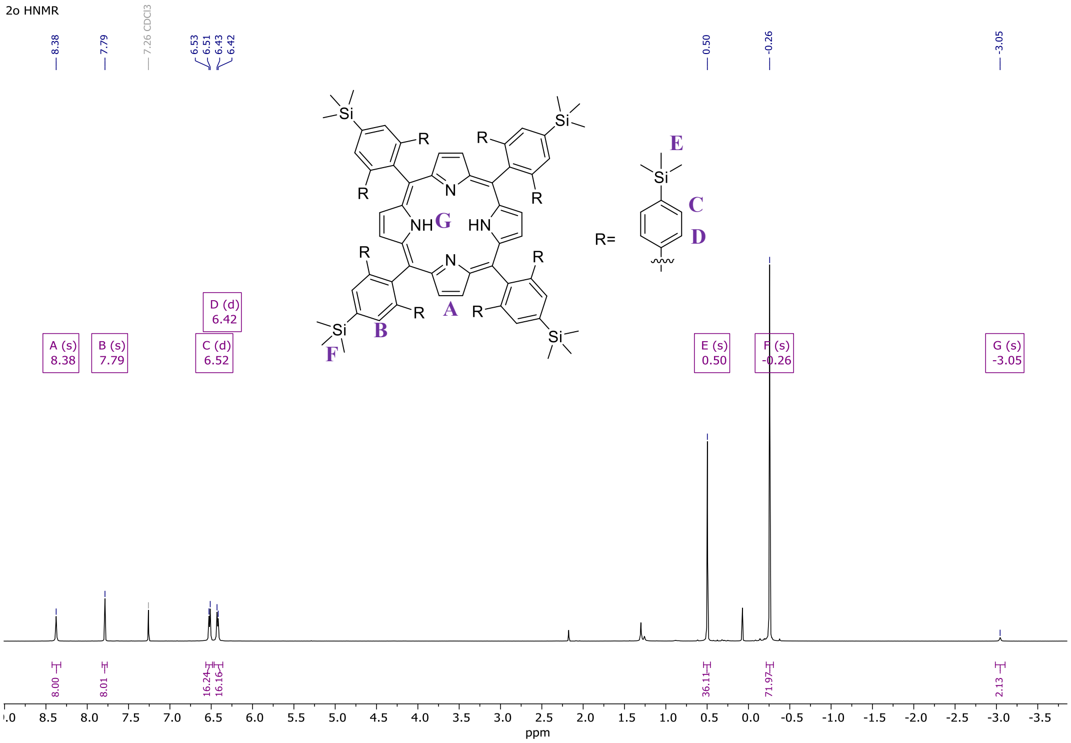

2o $^{13}C\{^{1}H\}$ NMR (126 MHz, $CDCl_3$)

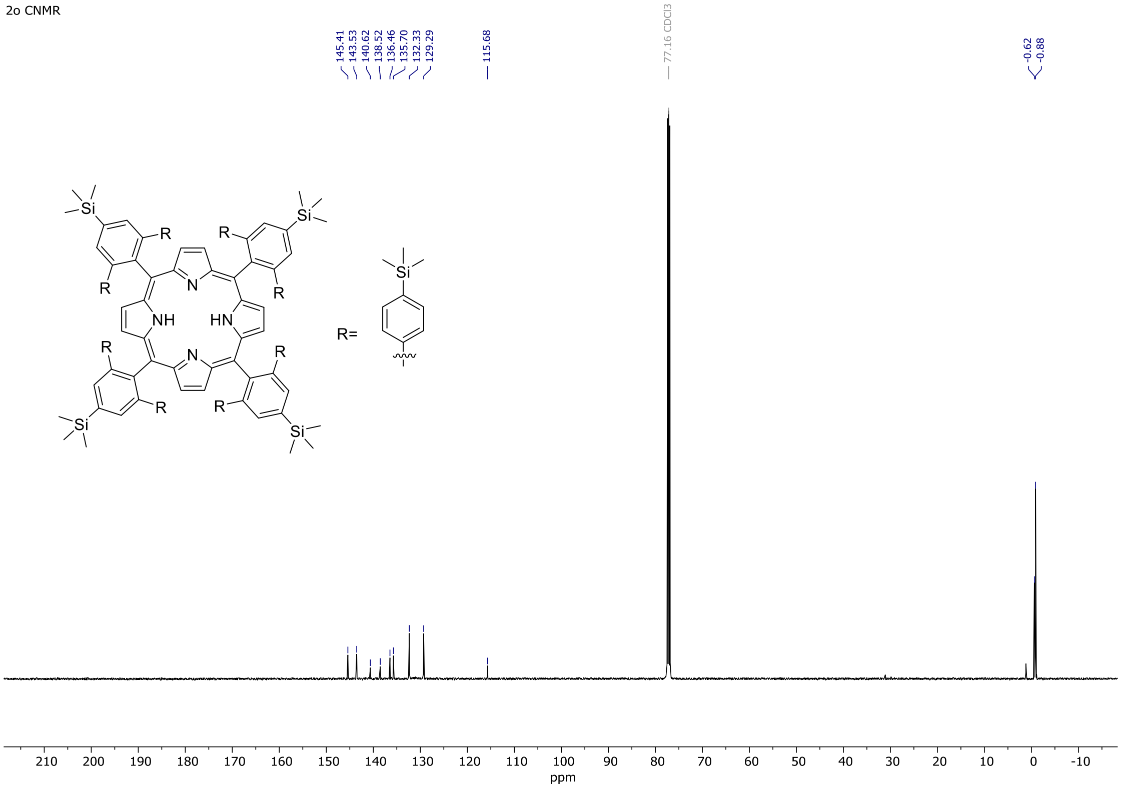

2p ^{1}H NMR (500 MHz, $CDCl_3$)

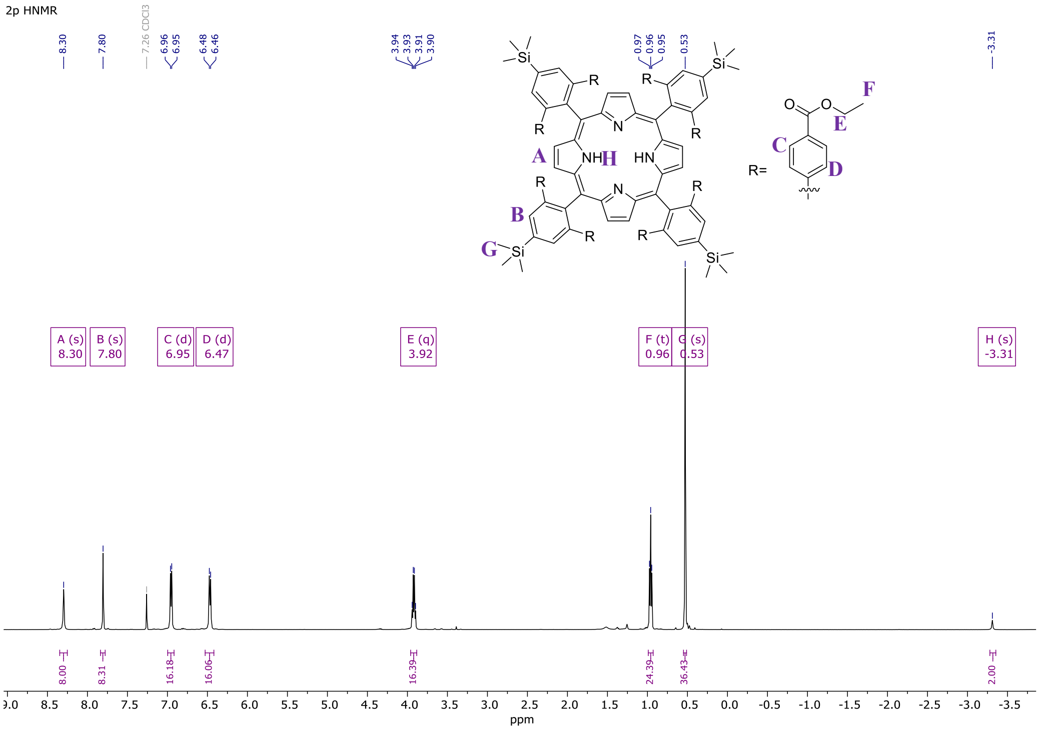

2p $^{13}C\{^{1}H\}$ NMR (126 MHz, $CDCl_3$)

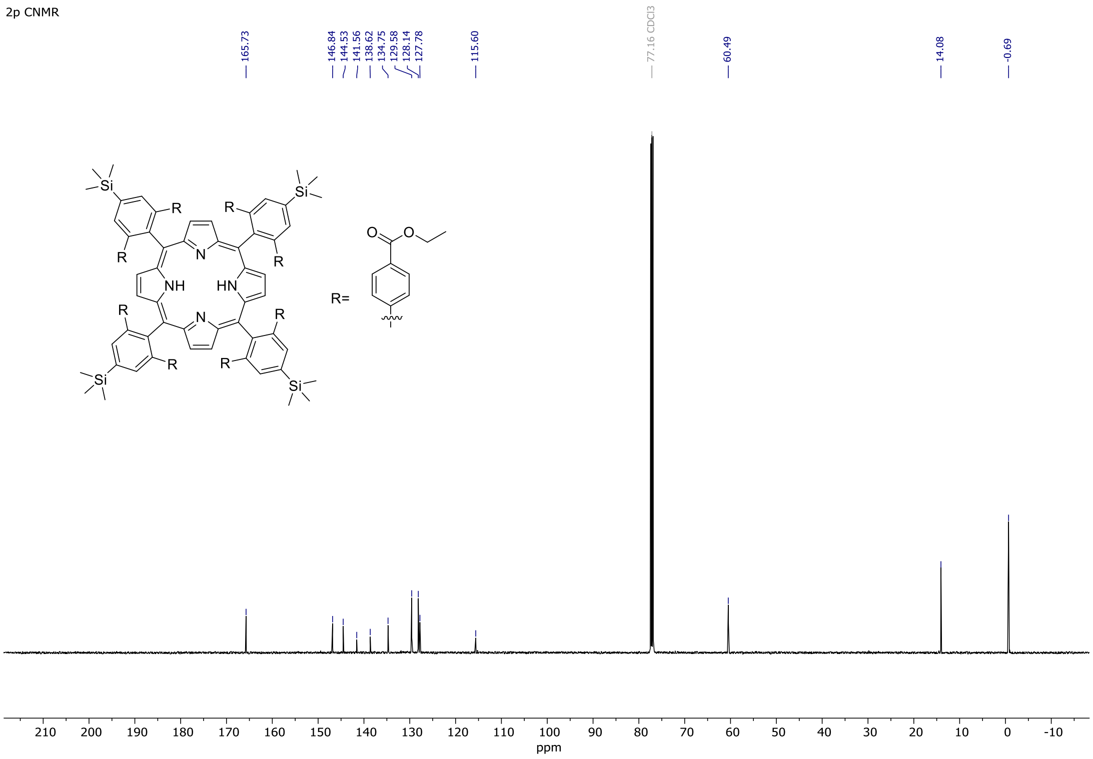

2q ^{1}H NMR (500 MHz, $CDCl_3$)

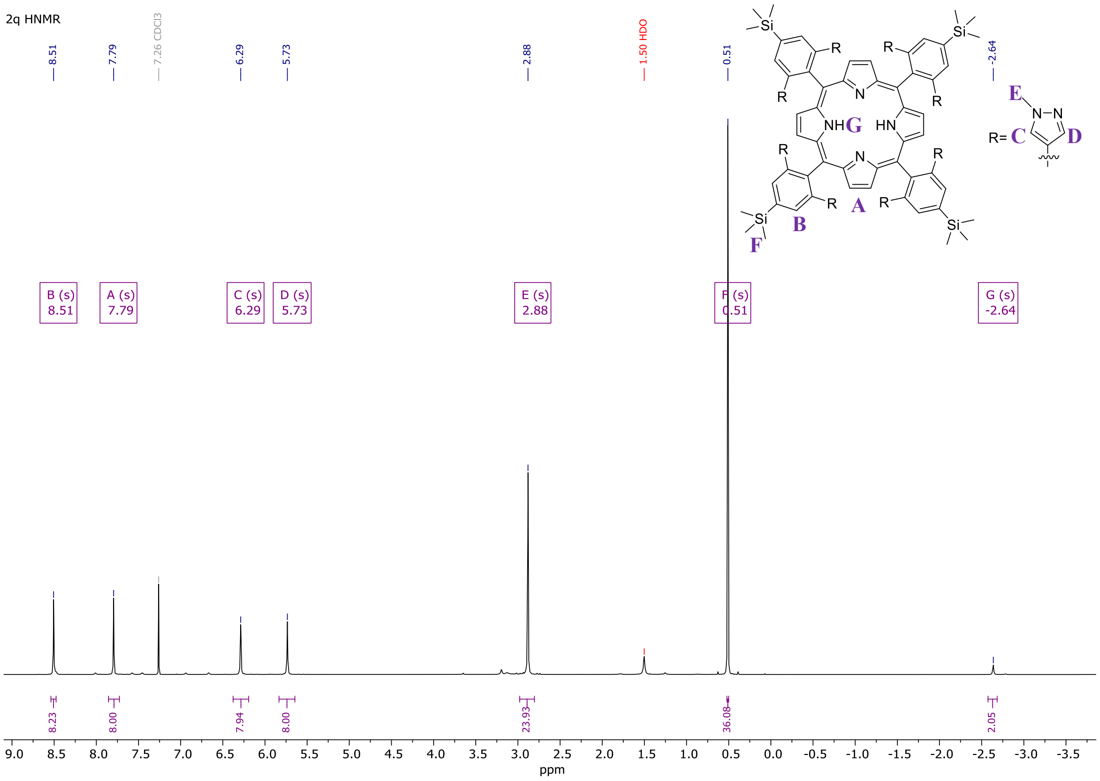

2q $^{13}C\{^1H\}$ NMR (126 MHz, $CDCl_3$)

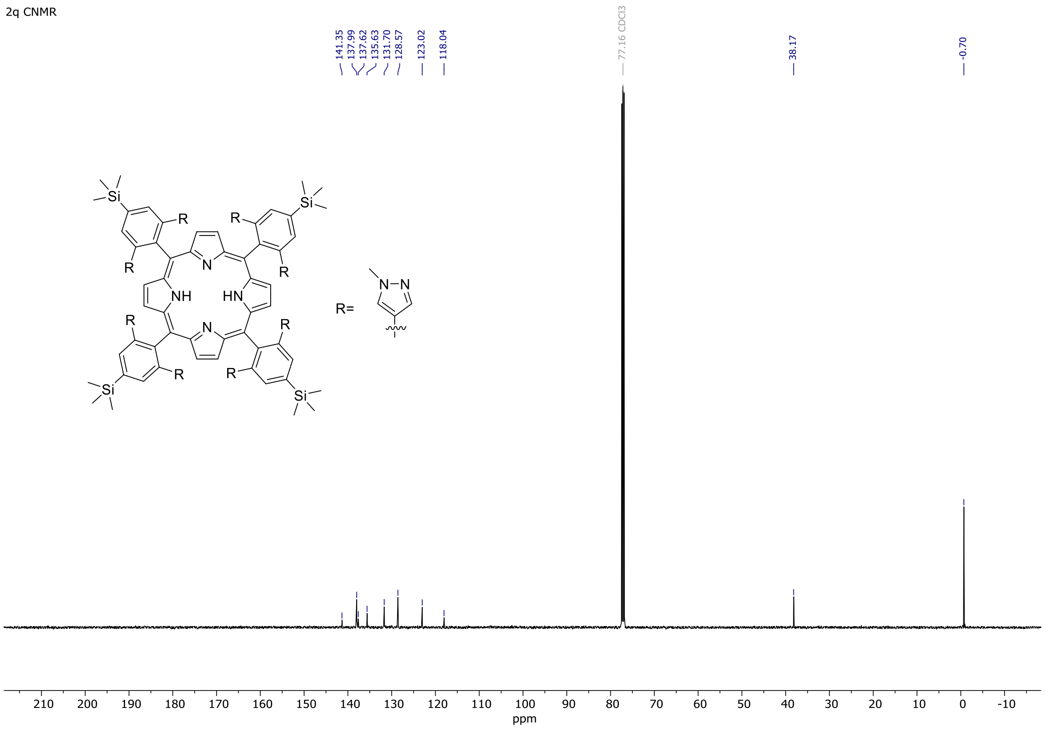

NMRs Sulfonation

3a ^1H NMR (500 MHz, DMSO-d_6)

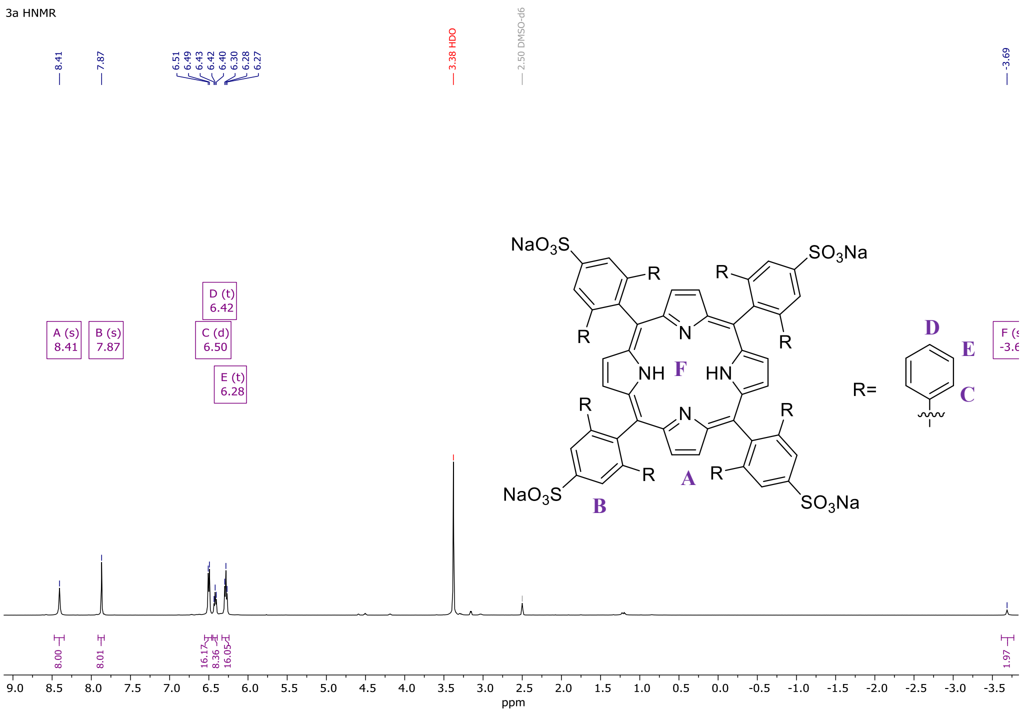

3a $^{13}C\{^1H\}$ NMR (126 MHz, DMSO-d₆)

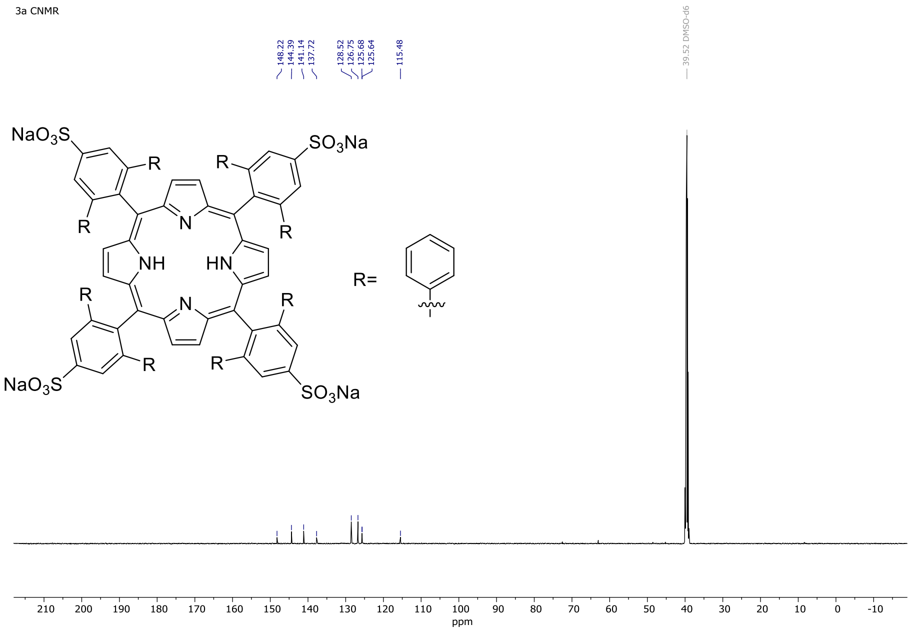

3b ^1H NMR (500 MHz, DMSO-d₆)

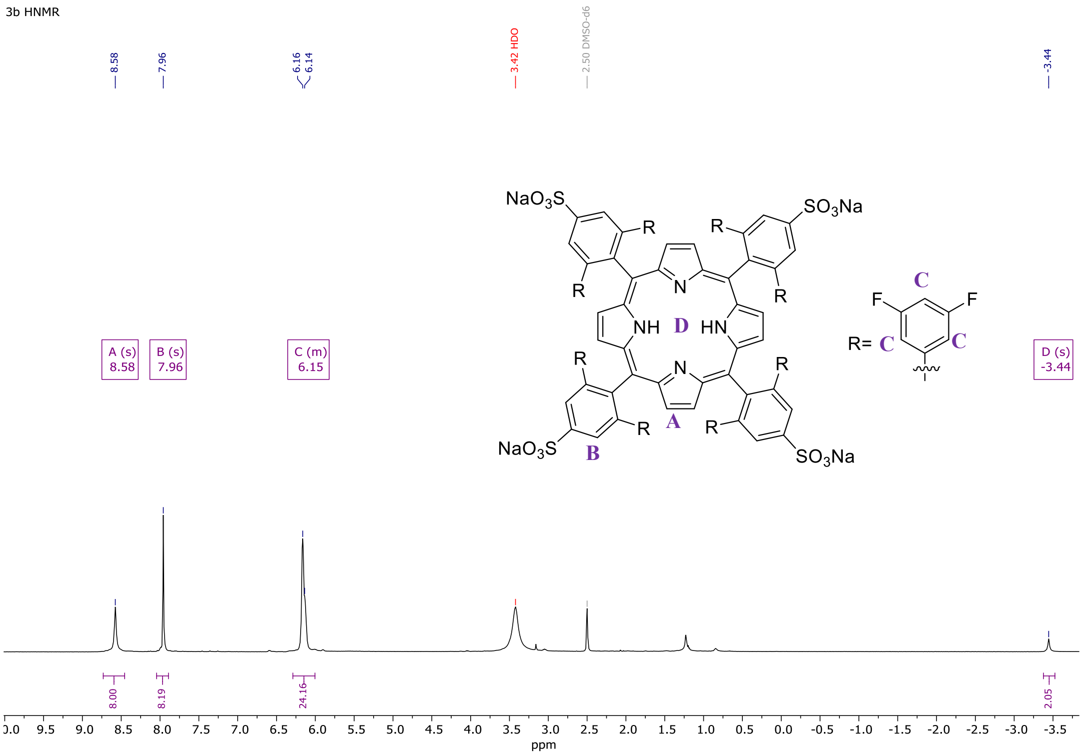

3b $^{19}F\{^1H\}$ NMR (470 MHz, DMSO-d$_6$)

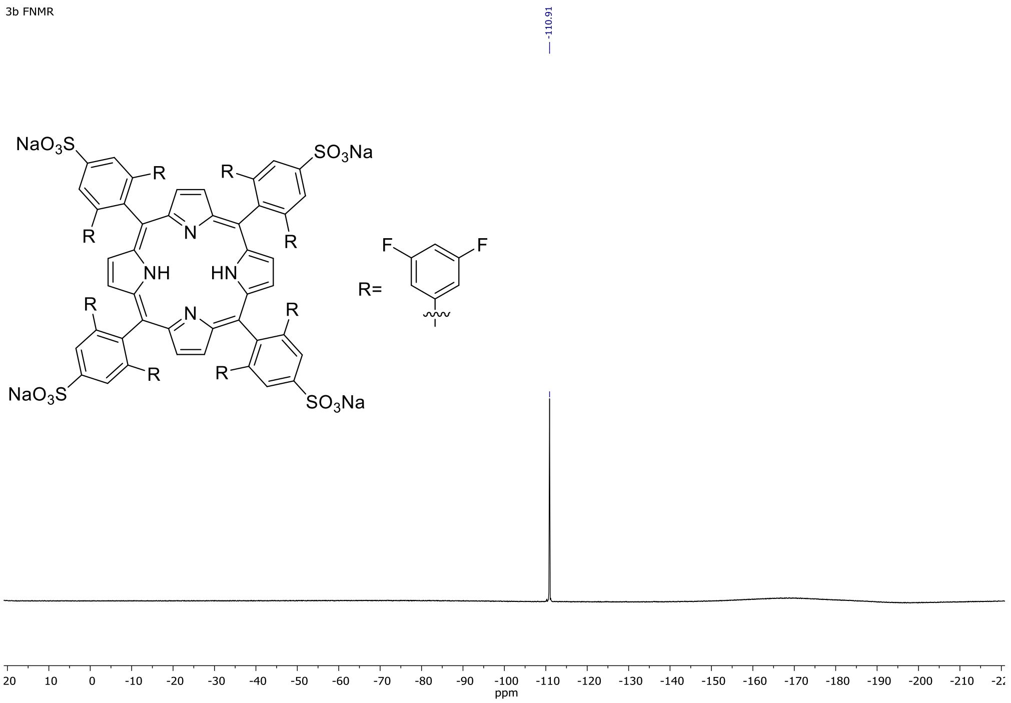

3b $^{13}C\{^1H\}$ NMR (126 MHz, DMSO-d$_6$)

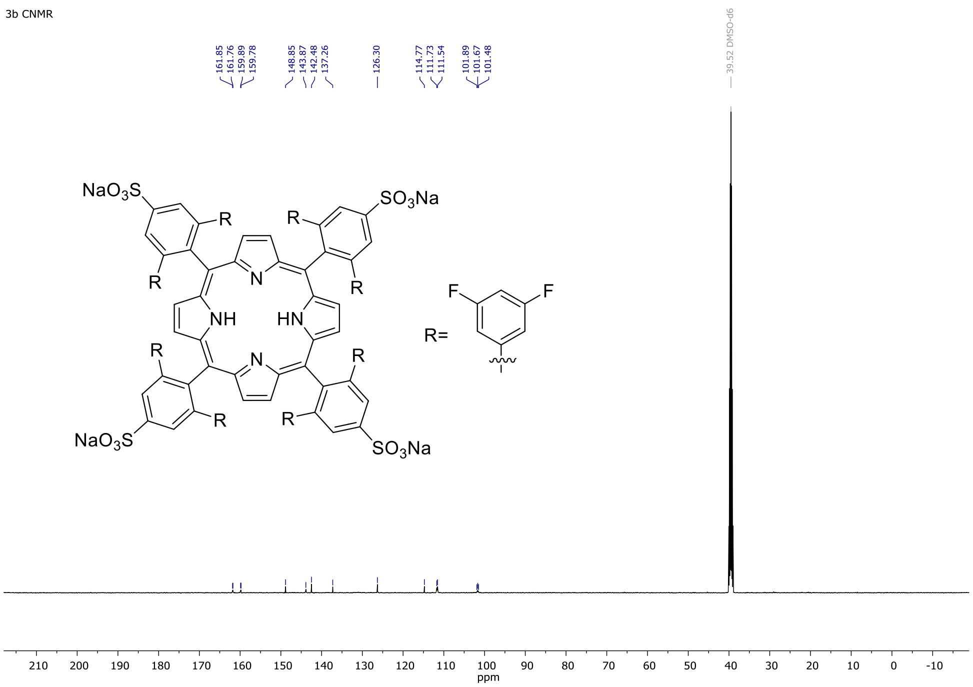

NMRs Metalations

4a ^{1}H NMR (500 MHz, $CDCl_3$)

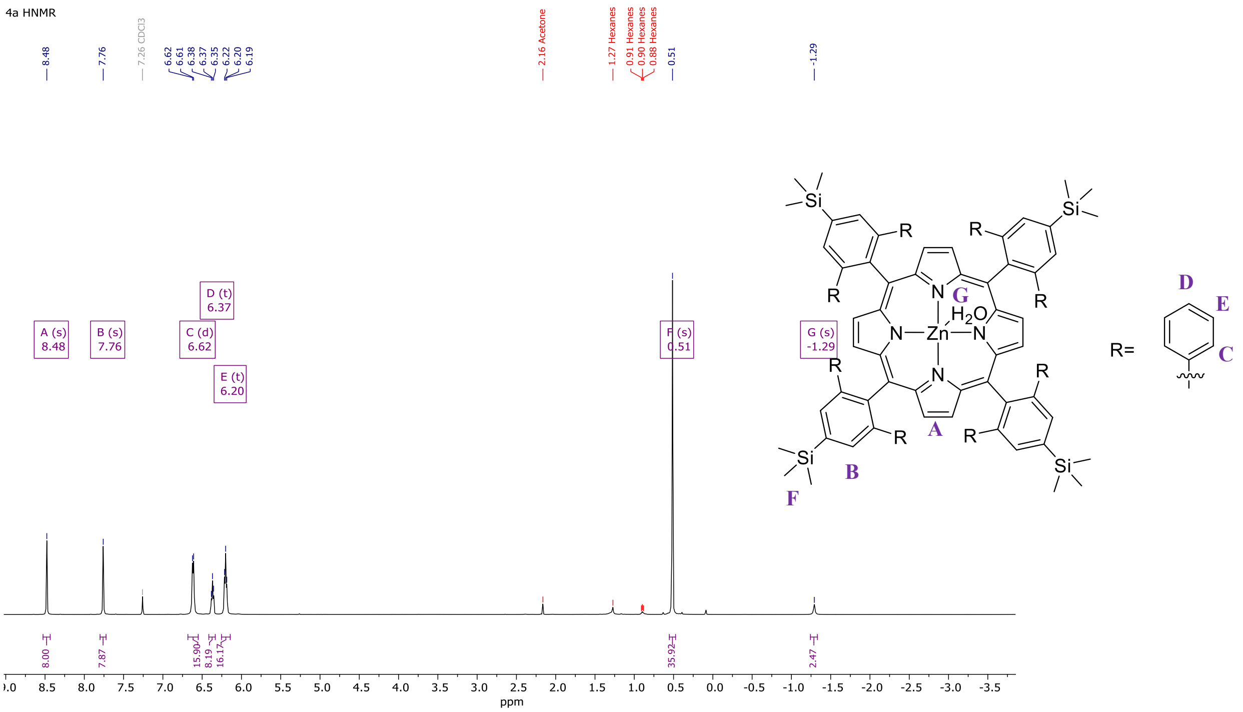

4a $^{13}C\{^{1}H\}$ NMR (126 MHz, $CDCl_3$)

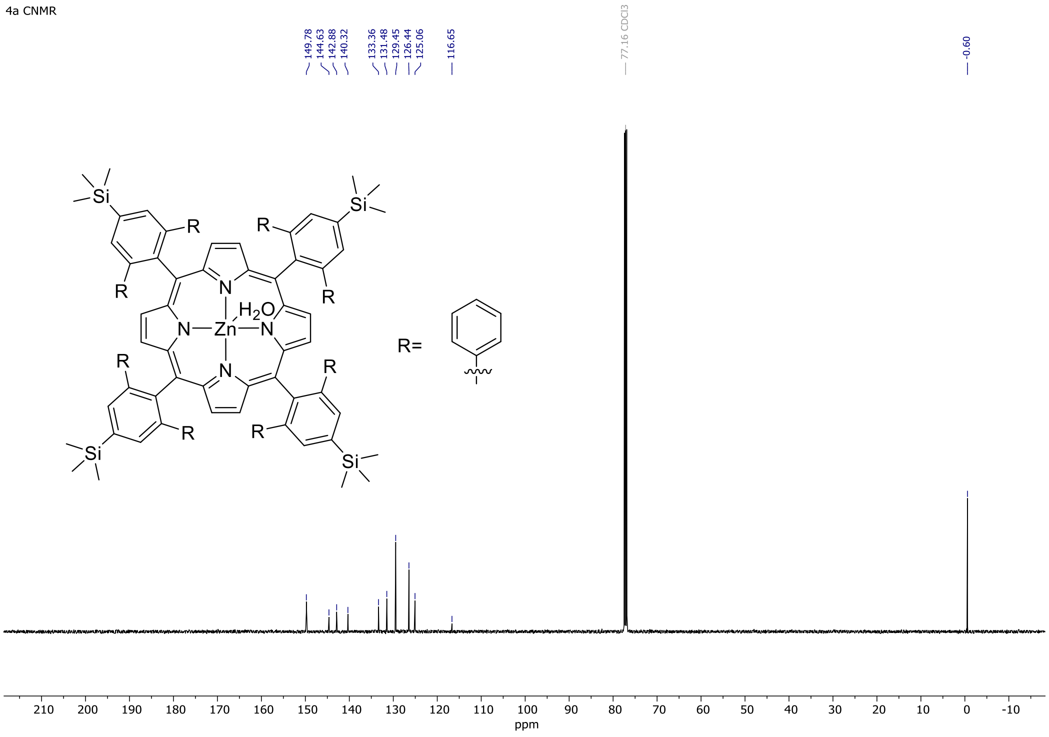

4b ^{1}H NMR (500 MHz, $CDCl_3$) (n.b. paramagnetic)

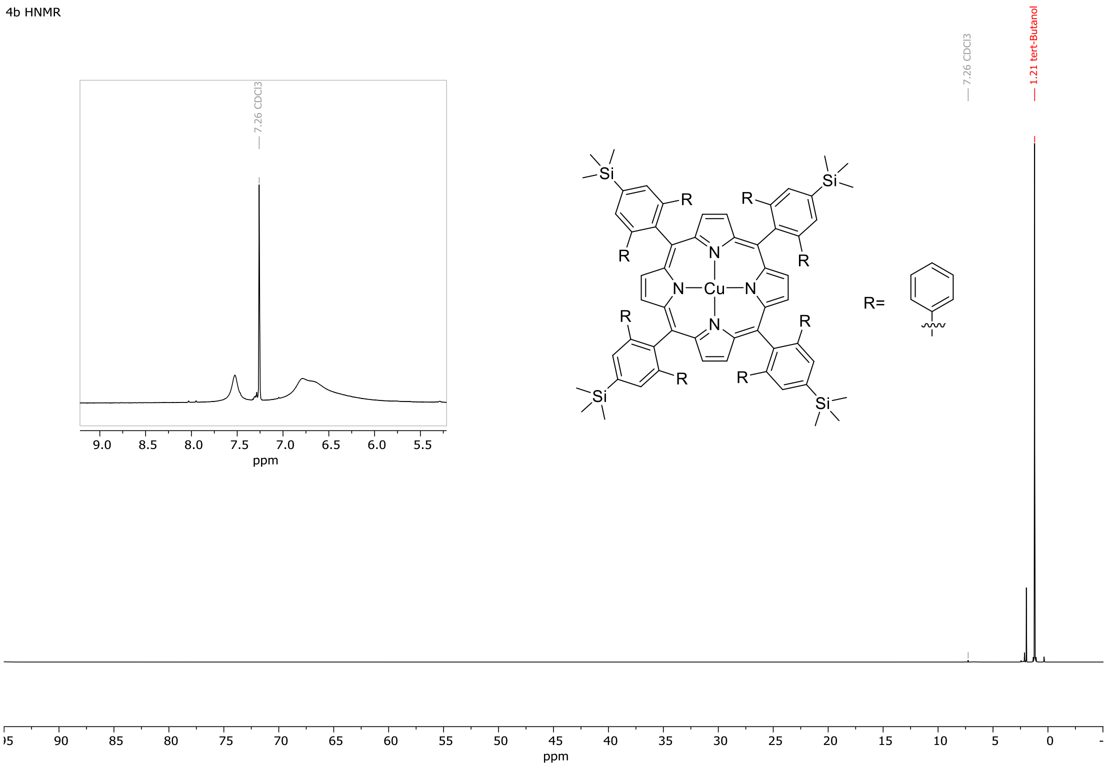

4c ^{1}H NMR (500 MHz, $CDCl_3$)

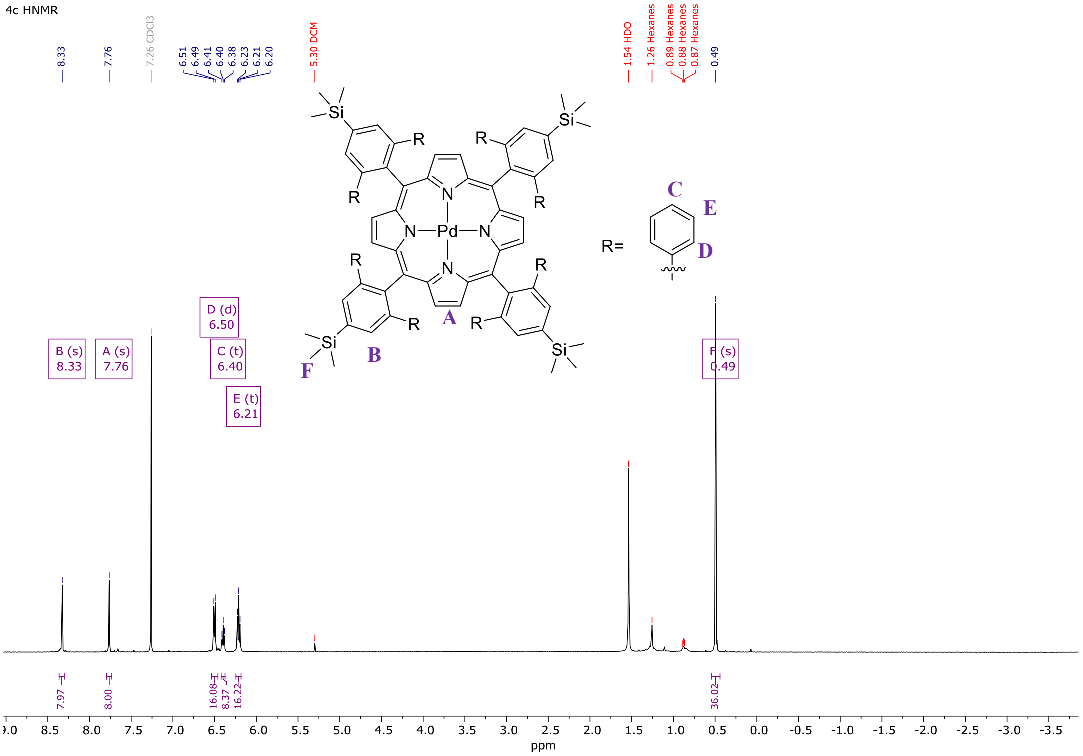

4c $^{13}C\{^1H\}$ NMR (126 MHz, $CDCl_3$)

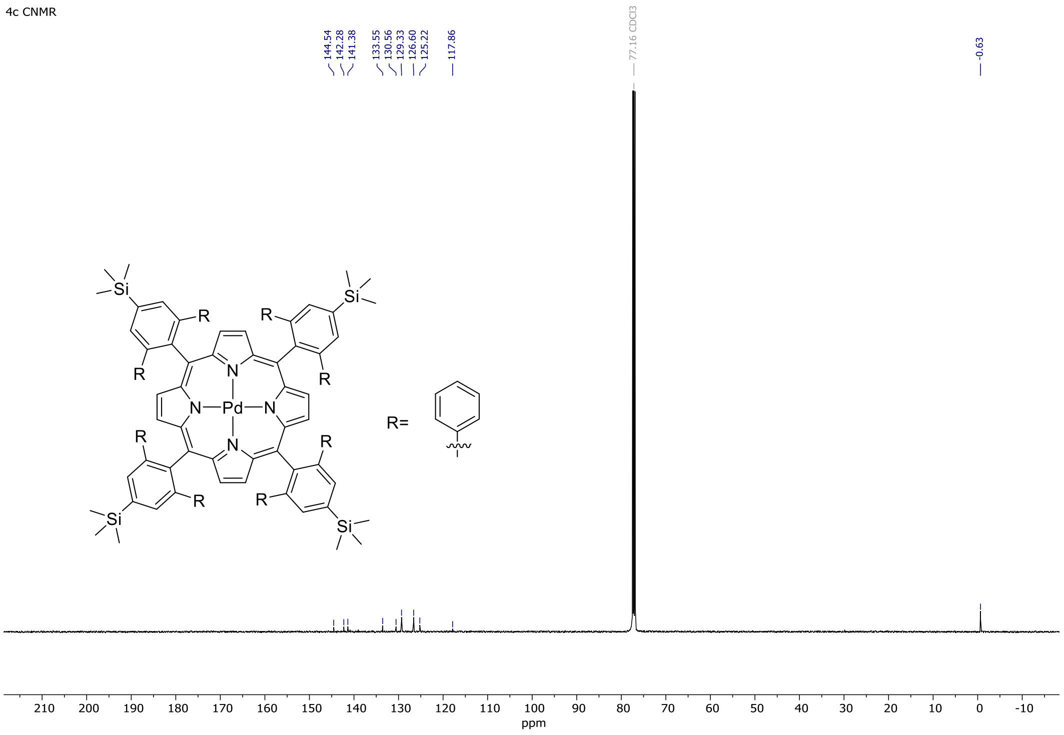

4d ^1H NMR (500 MHz, $CDCl_3$) (n.b. paramagnetic)

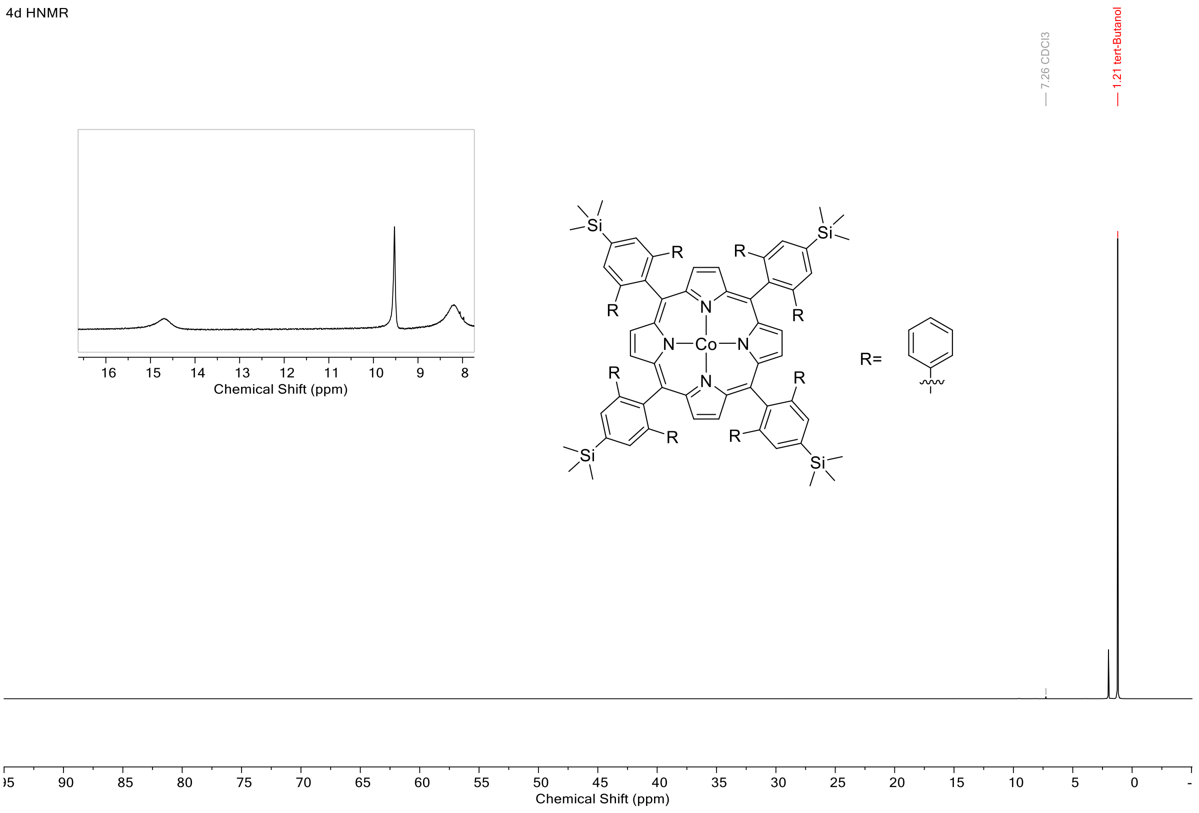

4e ^{1}H NMR (500 MHz, $CDCl_3$) (n.b. paramagnetic)

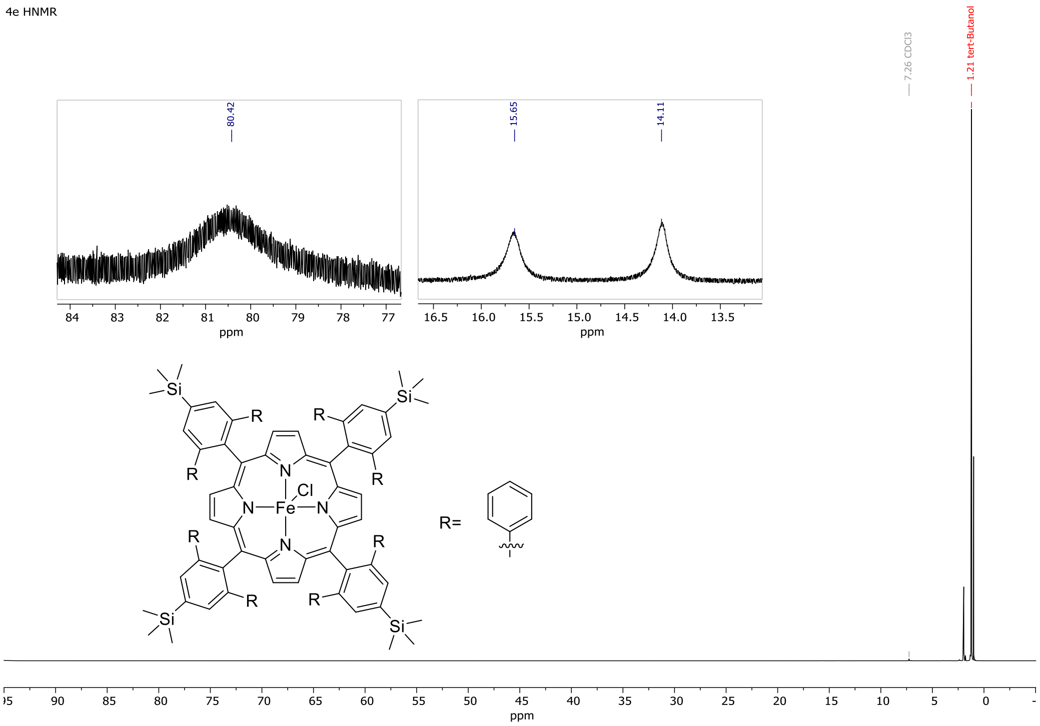

4f ^{1}H NMR (500 MHz, $CDCl_3$) (n.b. paramagnetic)

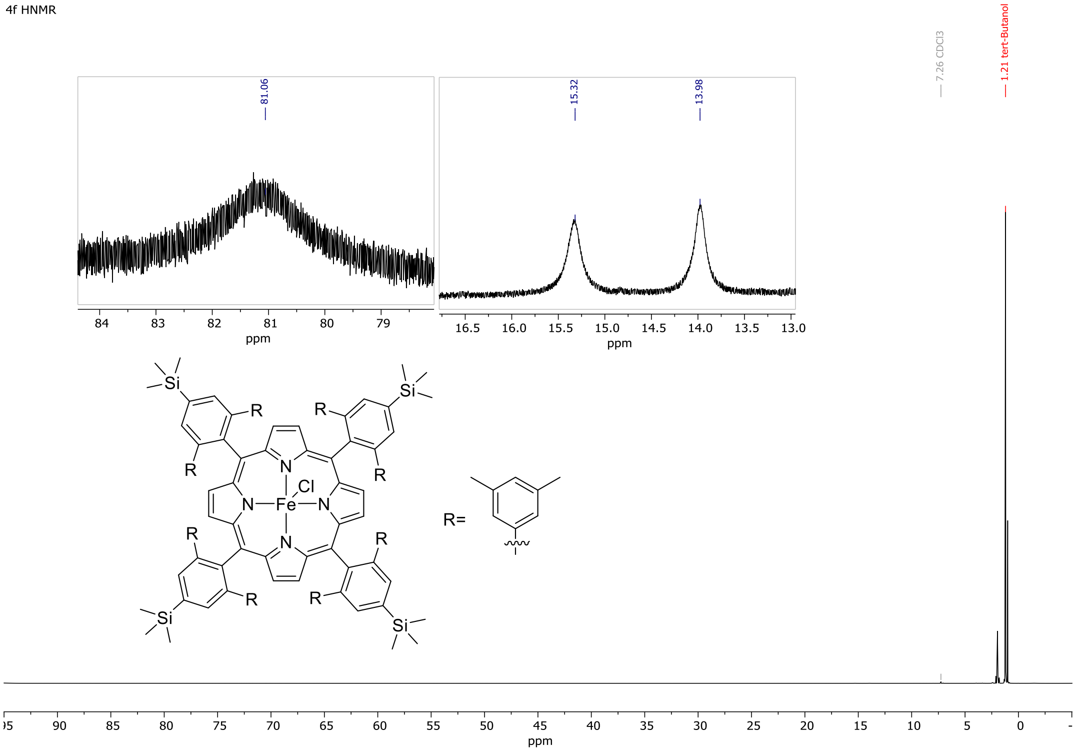

High-Performance Liquid Chromatography

4b

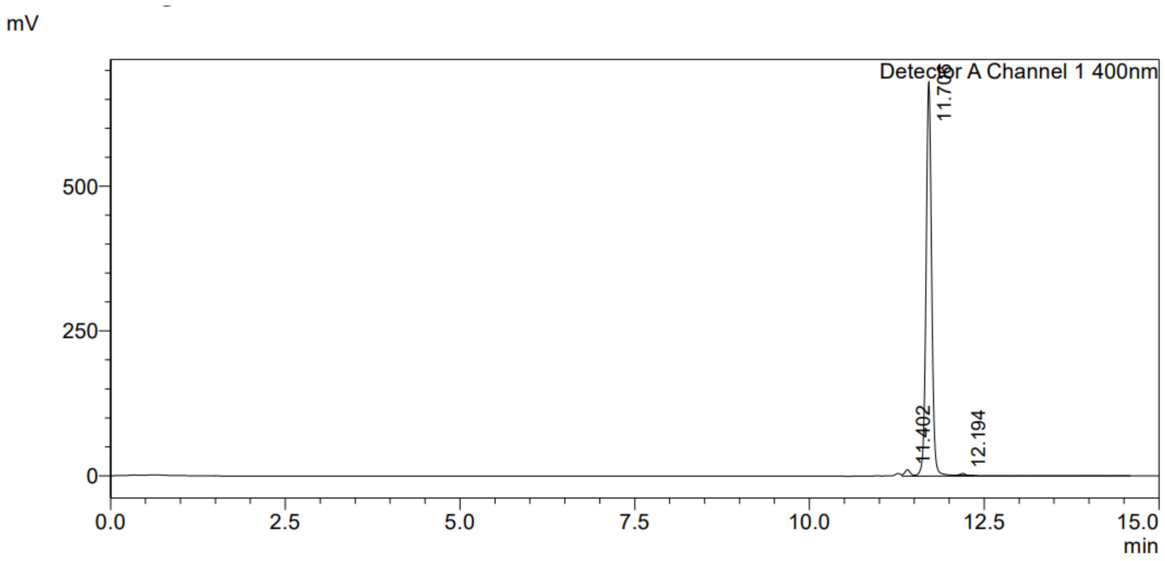

Figure S1. HPLC chromatogram of **4b** confirming >95% purity. Absorbance is measured at 400 nm and the analyte was eluted with a Hexane/DCM gradient of 0-100% DCM over 15 min.

4d

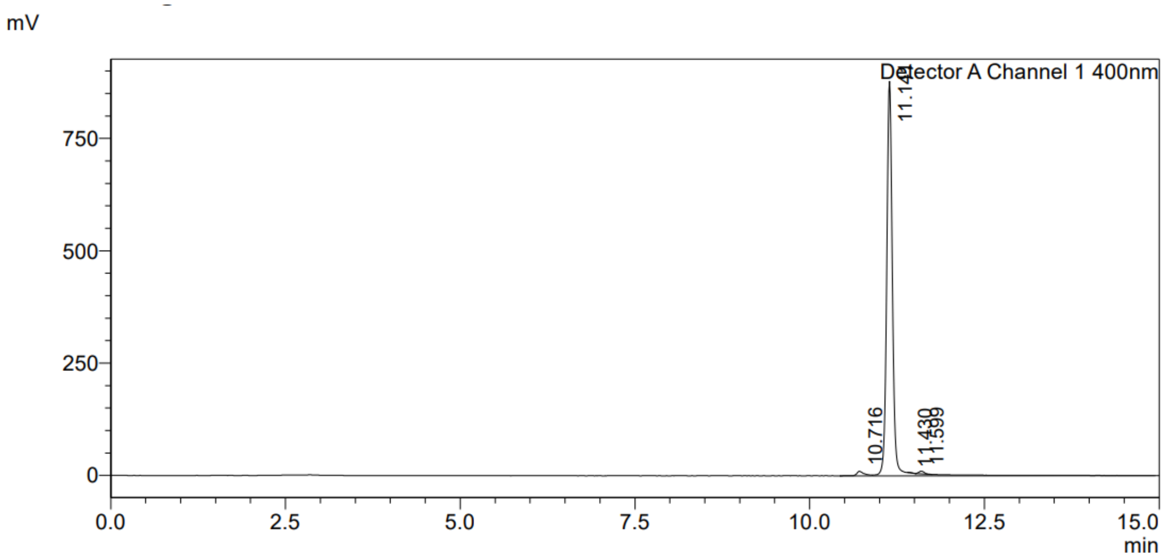

Figure S2. HPLC chromatogram of **4d** confirming >97% purity. Absorbance is measured at 400 nm and the analyte was eluted with a Hexane/DCM gradient of 0-100% DCM over 15 min.

4e

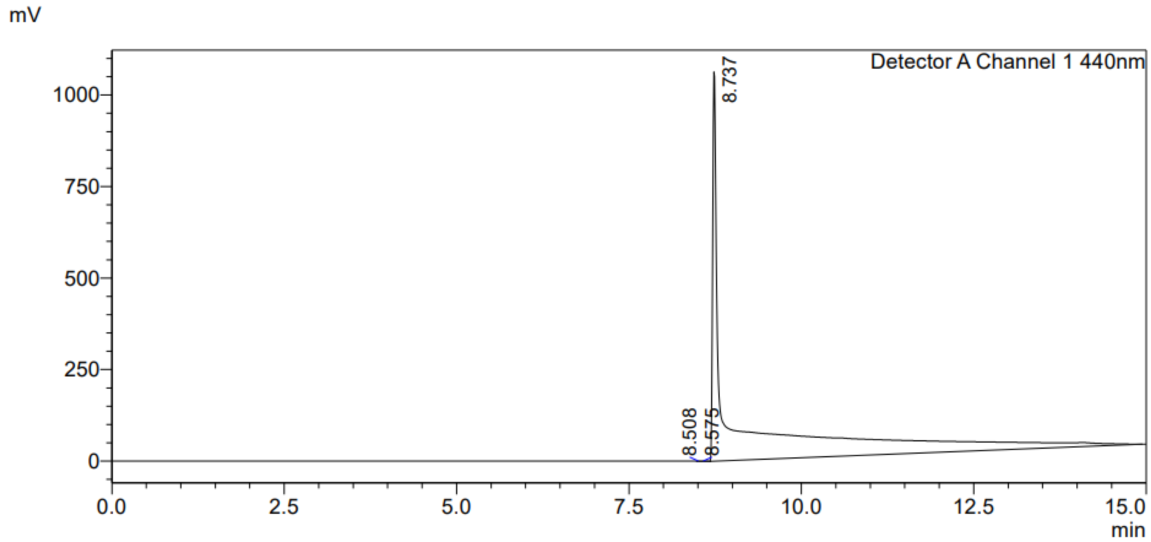

Figure S3. HPLC chromatogram of **4e** confirming >97% purity. Absorbance is measured at 440 nm and the analyte was eluted with a Hexane/DCM gradient of 0-100% DCM over 15 min.

4f

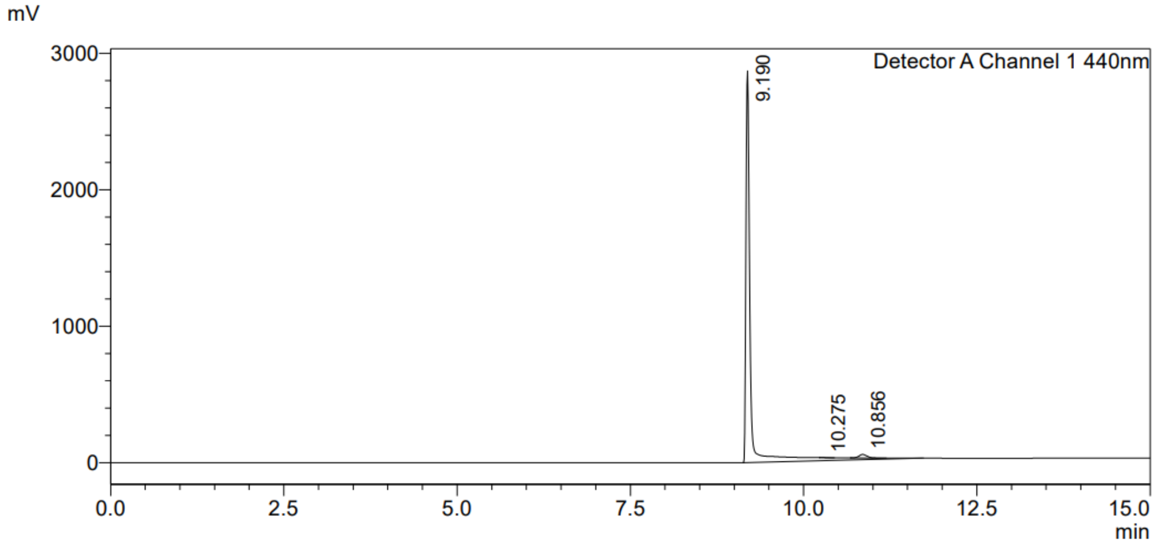

Figure S4. HPLC chromatogram of **4f** confirming >97% purity. Absorbance is measured at 440 nm and the analyte was eluted with a Hexane/DCM gradient of 0-100% DCM over 15 min.

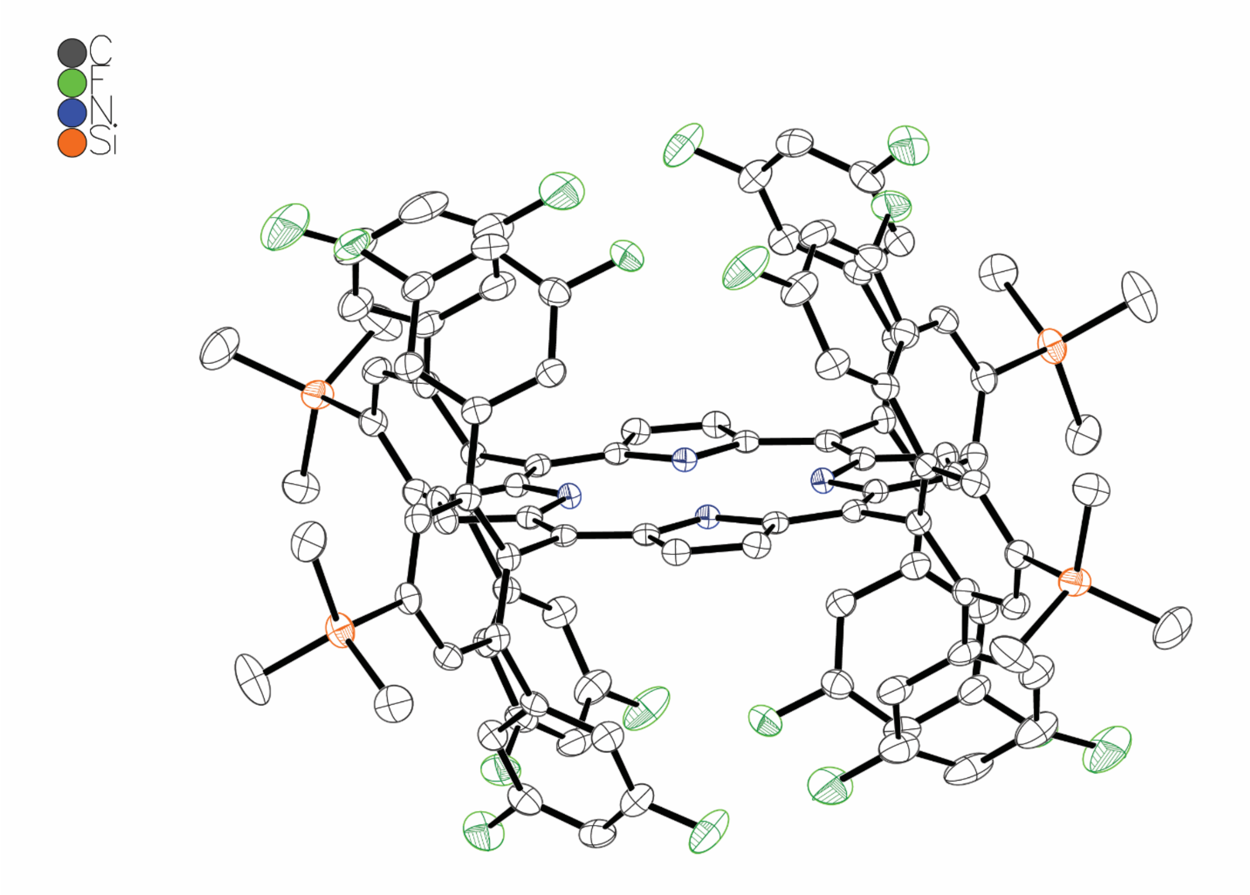

Figure S5. Thermal ellipsoid plot (50% ellipsoids) of the crystal structure of **2b**. H atoms and solvent omitted for clarity.

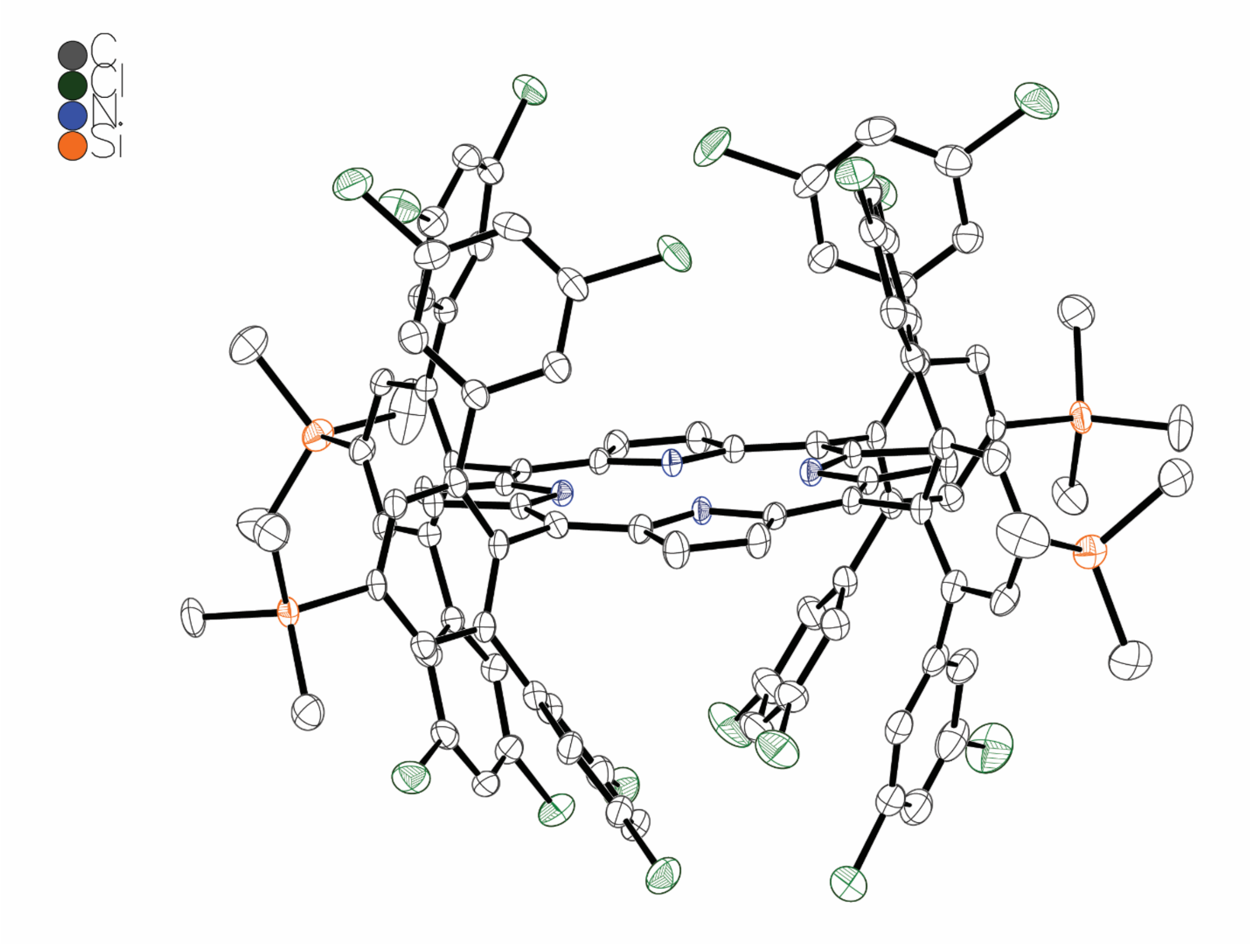

Figure S6. Thermal ellipsoid plot (50% ellipsoids) of the crystal structure of **2c**. H atoms and solvent omitted for clarity.

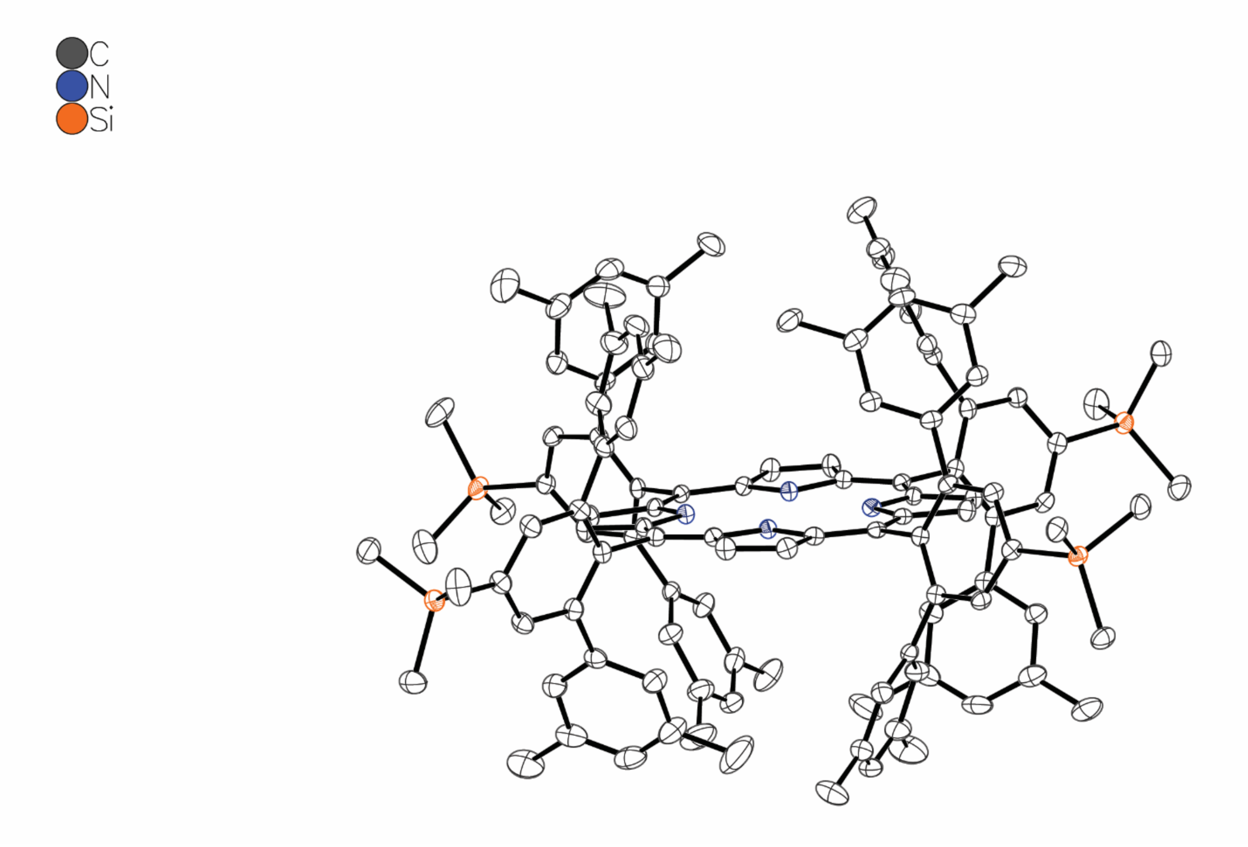

Figure S7. Thermal ellipsoid plot (50% ellipsoids) of the crystal structure of **2d**. H atoms and solvent omitted for clarity.

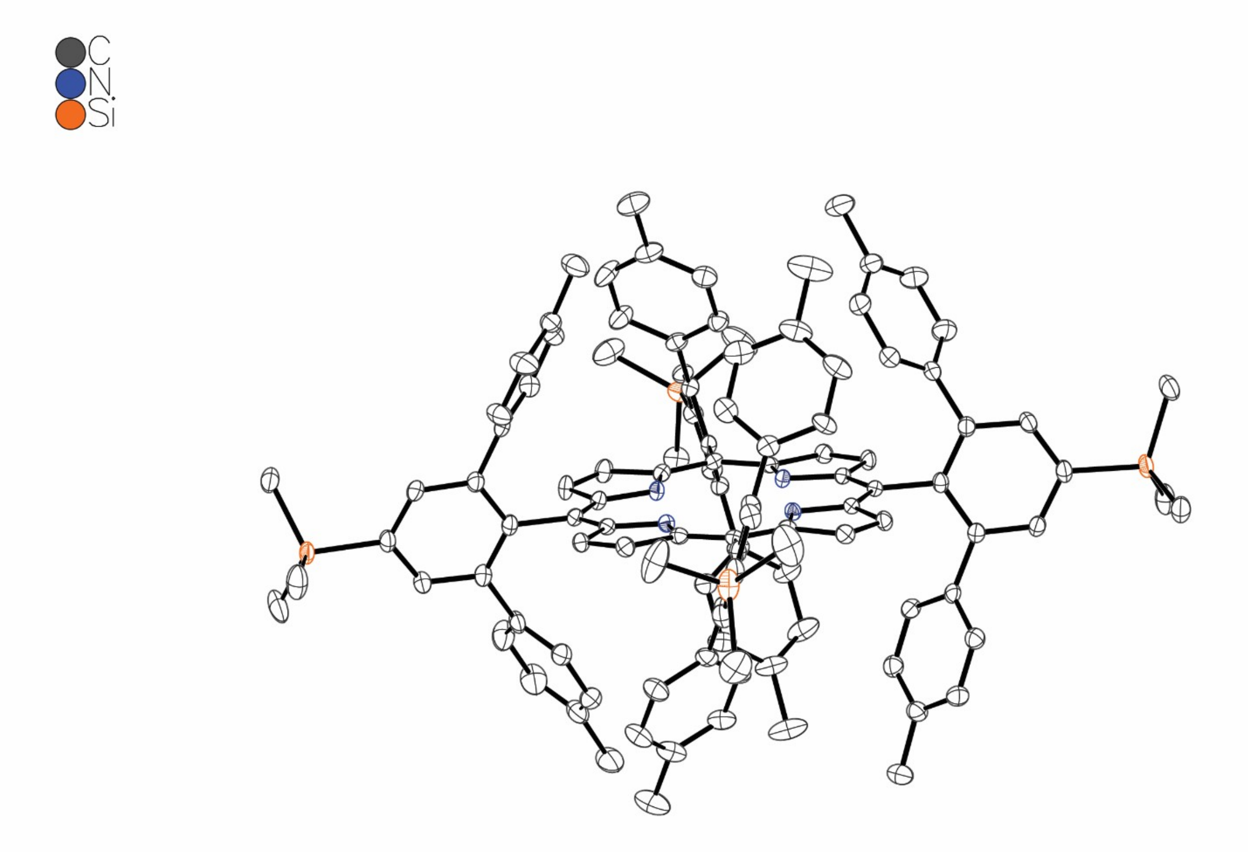

Figure S8. Thermal ellipsoid plot (50% ellipsoids) of the crystal structure of **2e**. H atoms, solvent, and minor components of the disorder omitted for clarity.

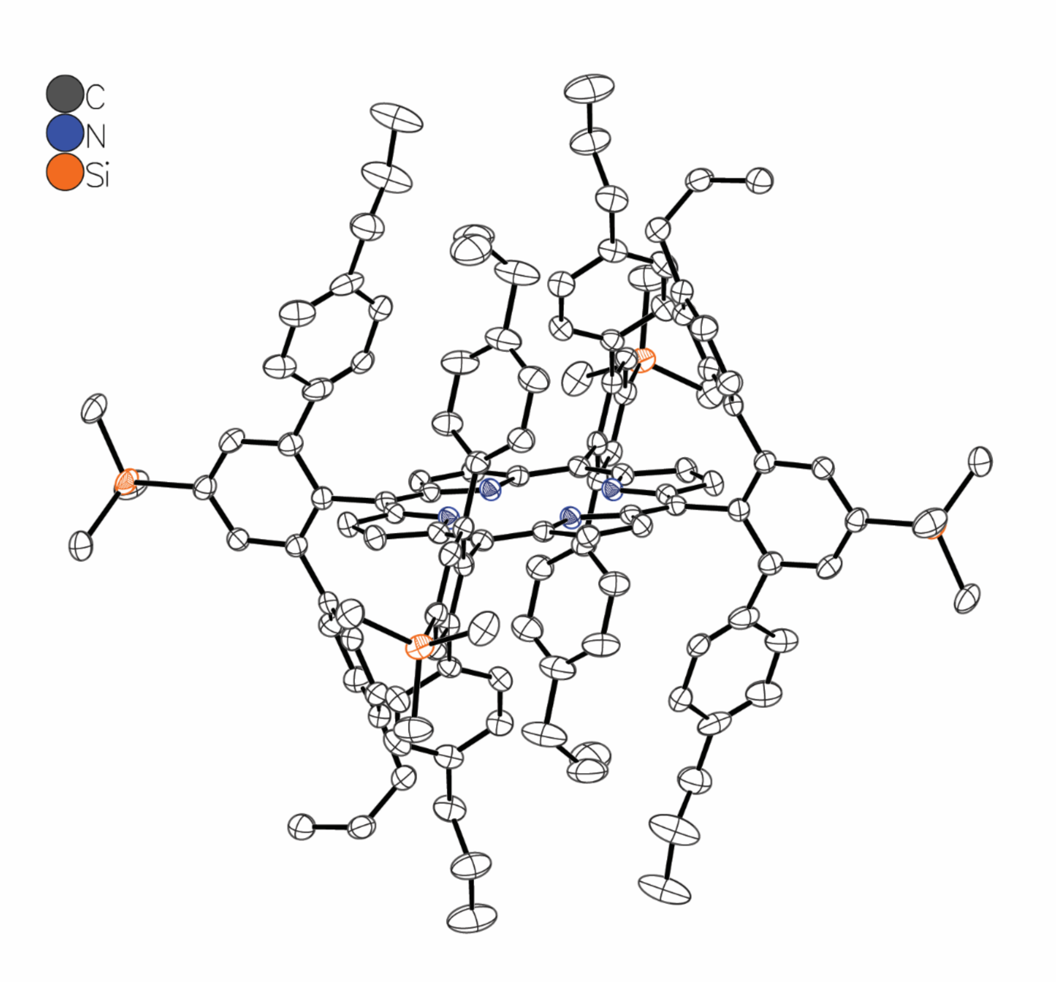

Figure S9. Thermal ellipsoid plot (50% ellipsoids) of the crystal structure of **2f**. H atoms, solvent, and minor components of the disorder omitted for clarity.

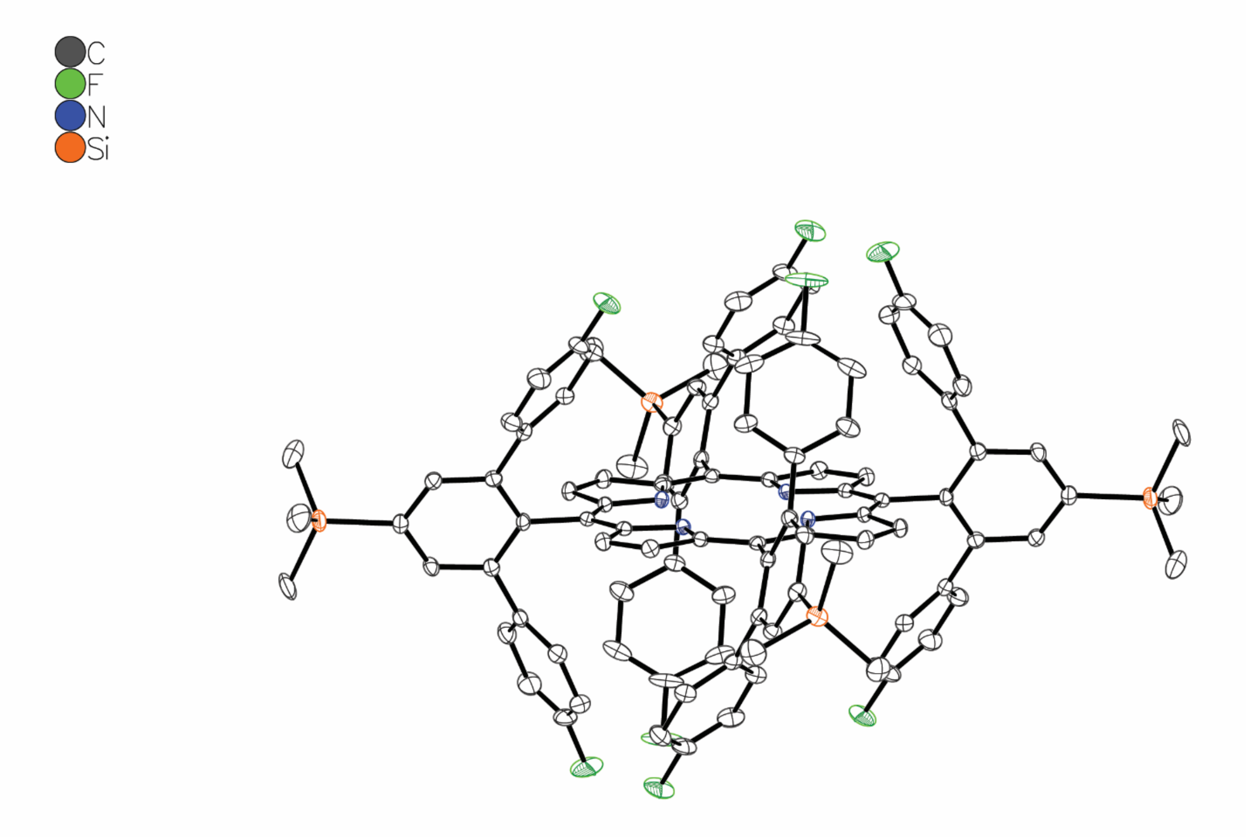

Figure S10. Thermal ellipsoid plot (50% ellipsoids) of the crystal structure of **2h**. H atoms and solvent omitted for clarity.

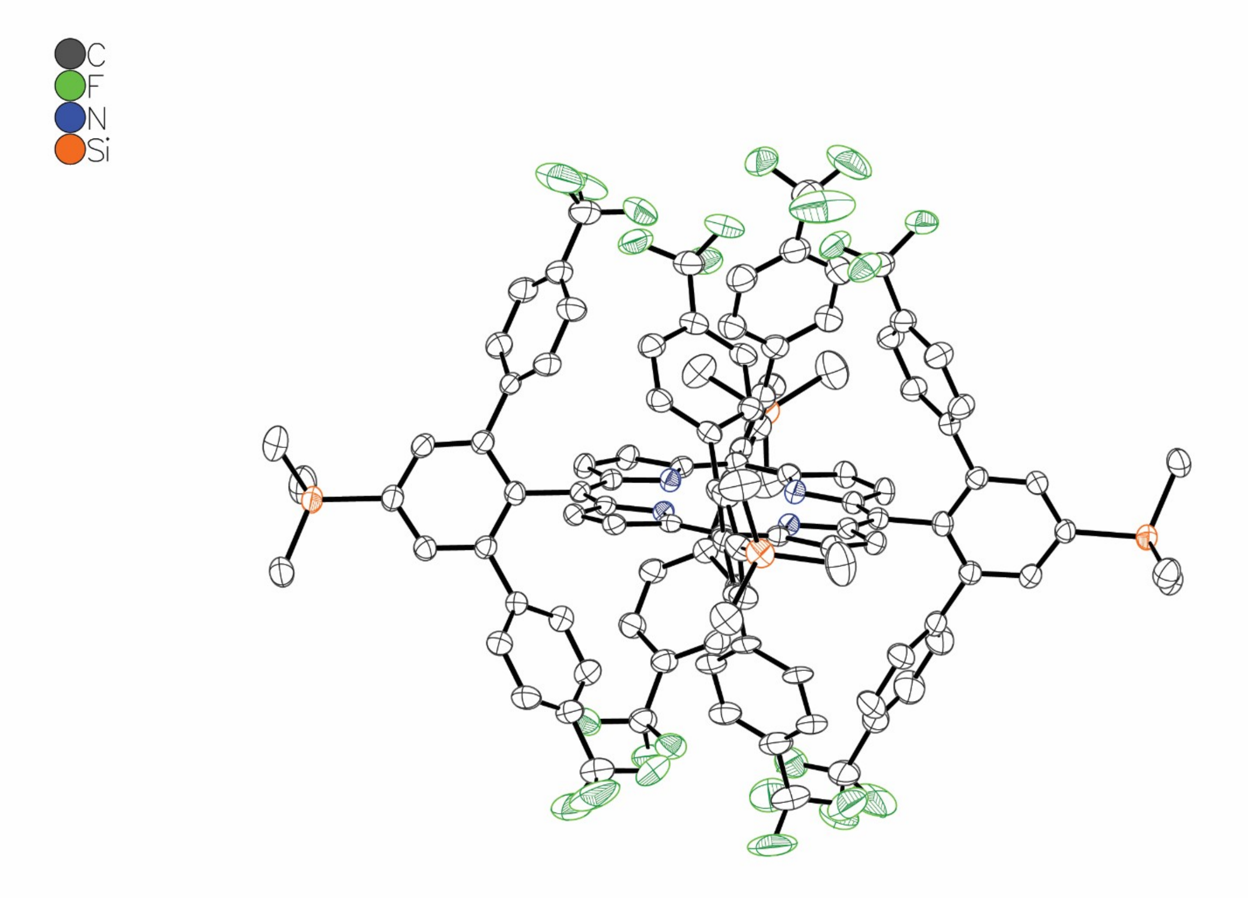

Figure S11. Thermal ellipsoid plot (50% ellipsoids) of the crystal structure of **2i**. H atoms, solvent, and minor components of the disorder omitted for clarity.

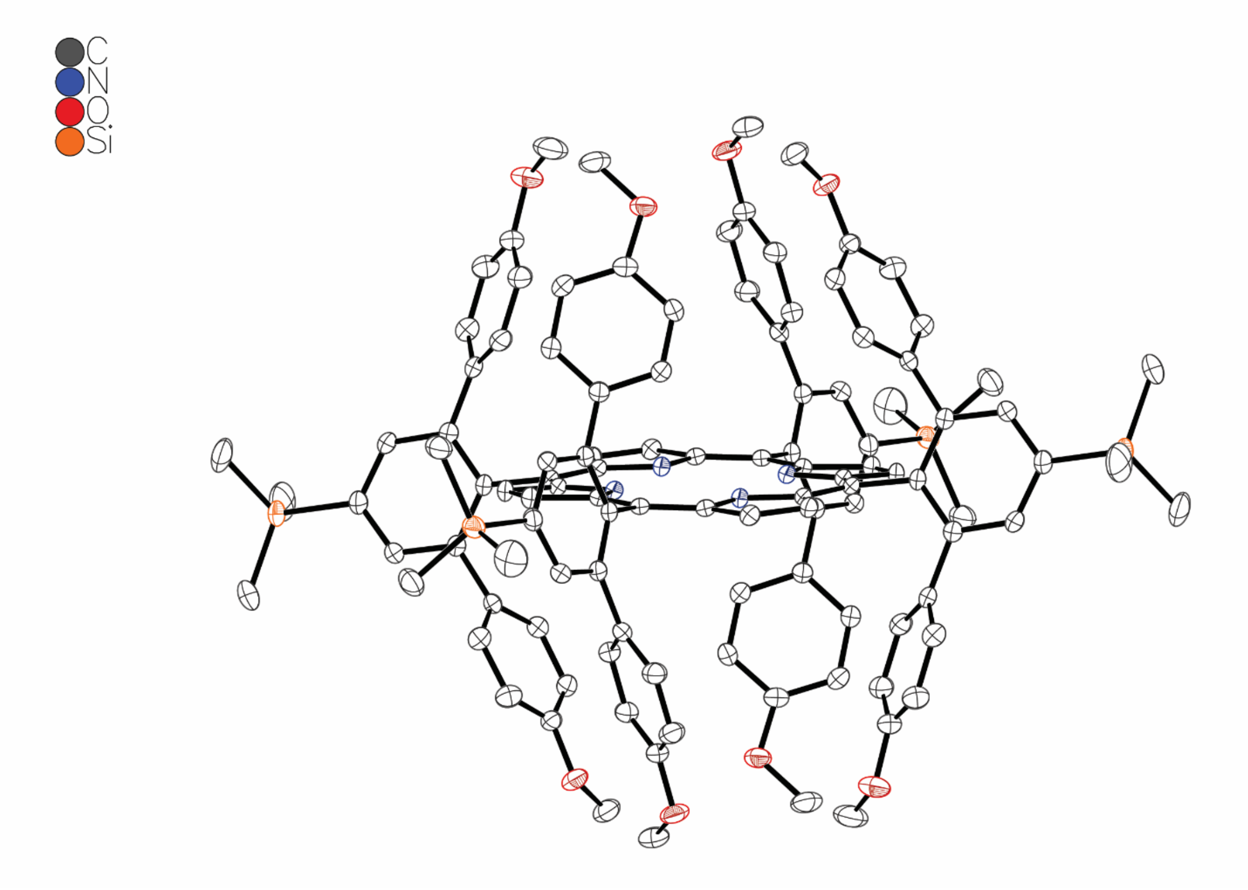

Figure S12. Thermal ellipsoid plot (50% ellipsoids) of the crystal structure of **2k**. H atoms and solvent omitted for clarity.

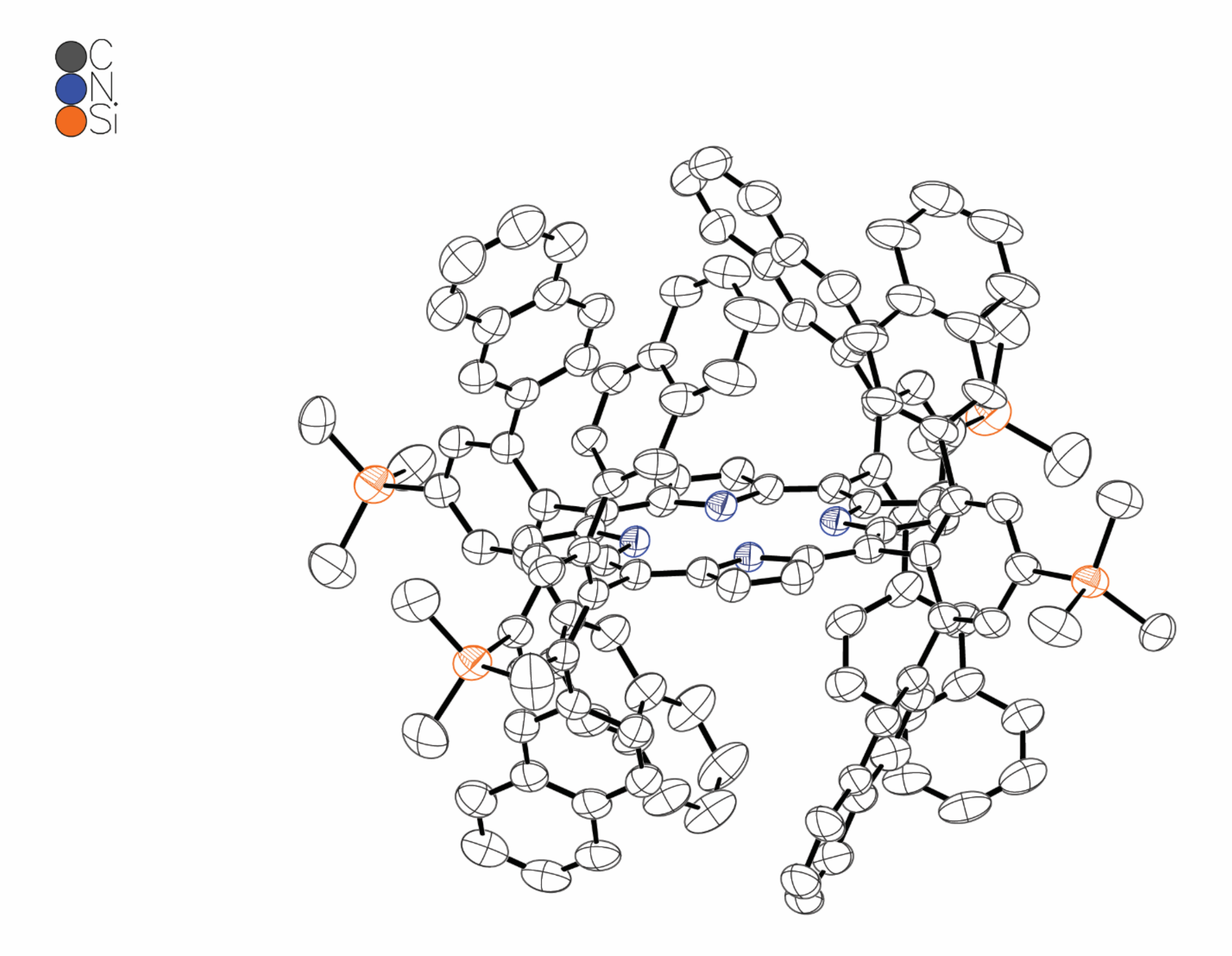

Figure S13. Thermal ellipsoid plot (50% ellipsoids) of the crystal structure of **2l**. H atoms, solvent, and minor components of the disorder omitted for clarity.

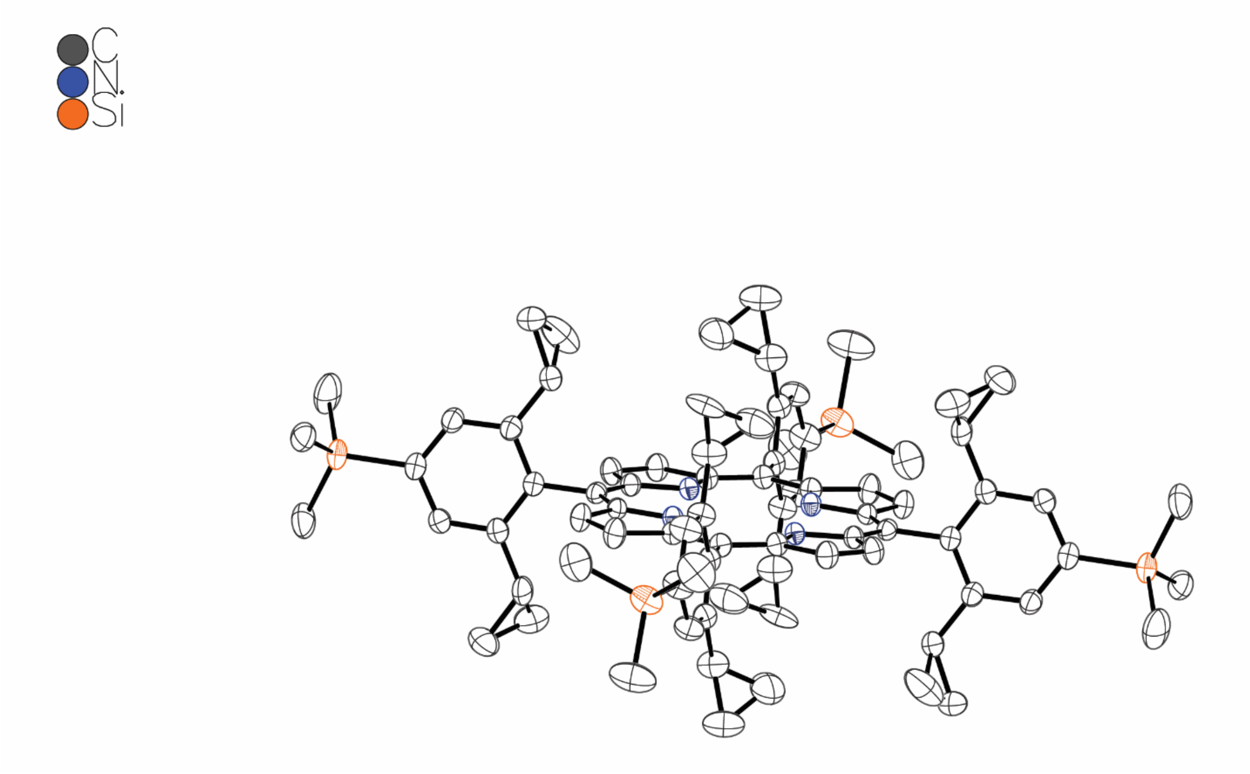

Figure S14. Thermal ellipsoid plot (50% ellipsoids) of the crystal structure of **2m**. H atoms, solvent, and minor components of the disorder omitted for clarity.

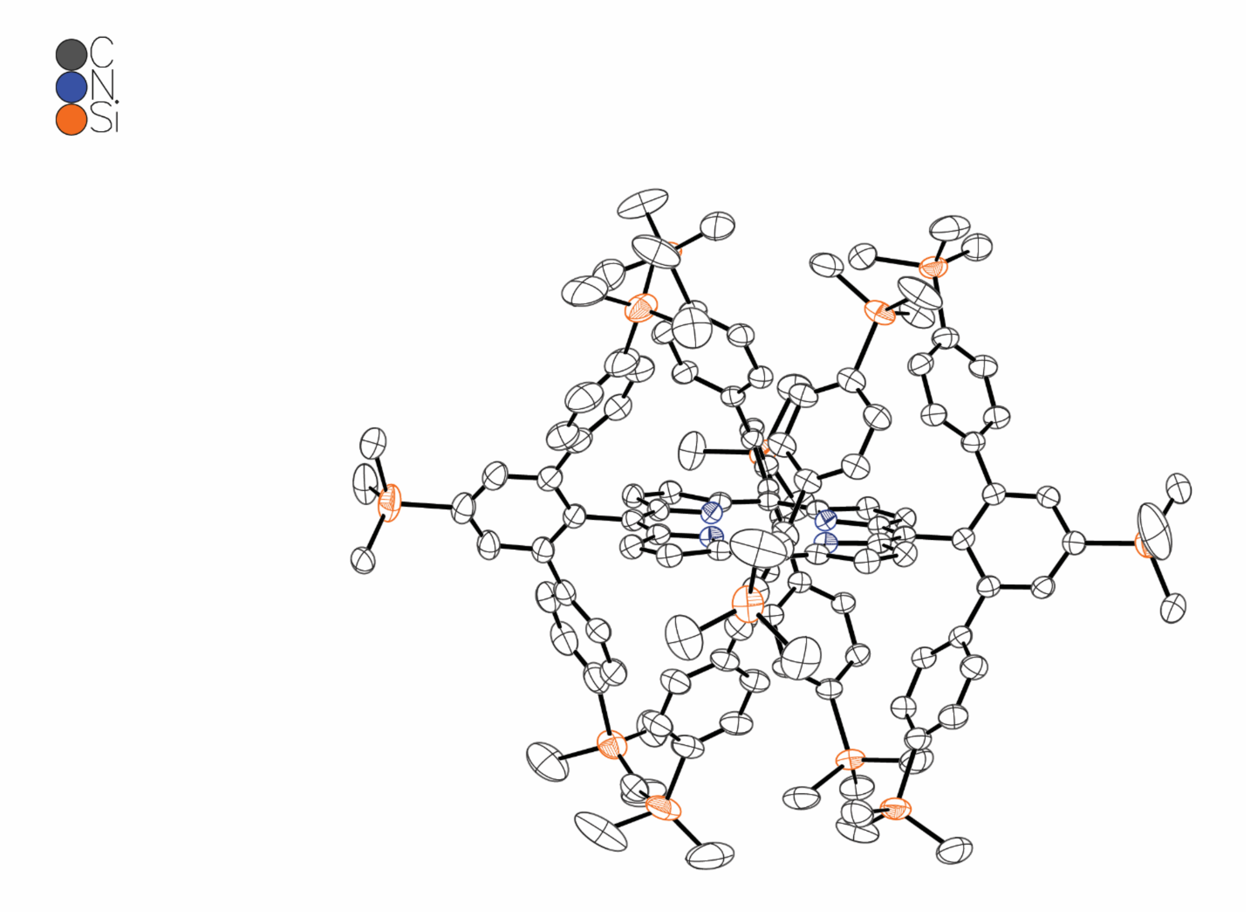

Figure S15. Thermal ellipsoid plot (50% ellipsoids) of the crystal structure of **2o**. H atoms, solvent, and minor components of the disorder omitted for clarity.

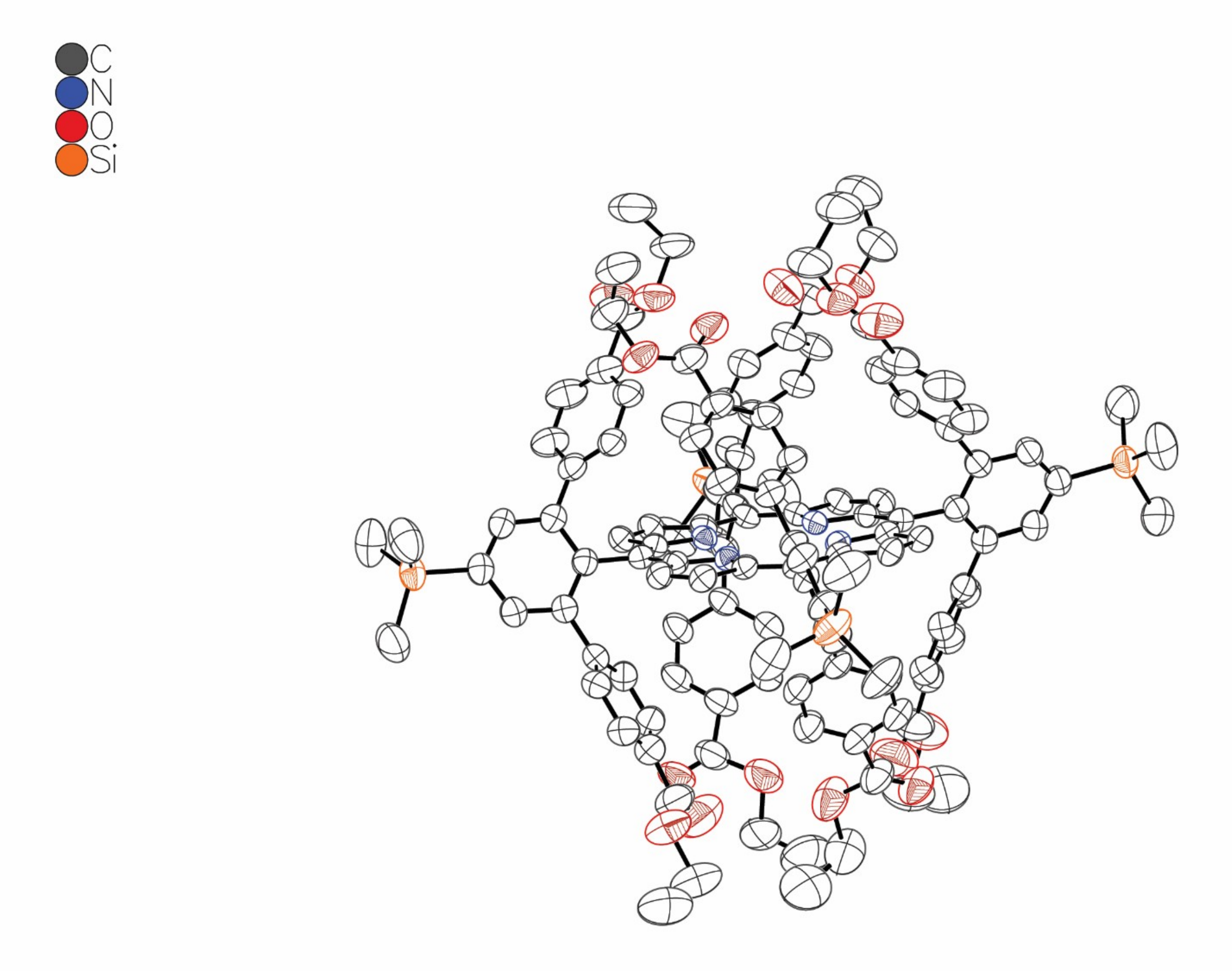

Figure S16. Thermal ellipsoid plot (50% ellipsoids) of the crystal structure of **2p**. H atoms, solvent, and minor components of the disorder omitted for clarity.

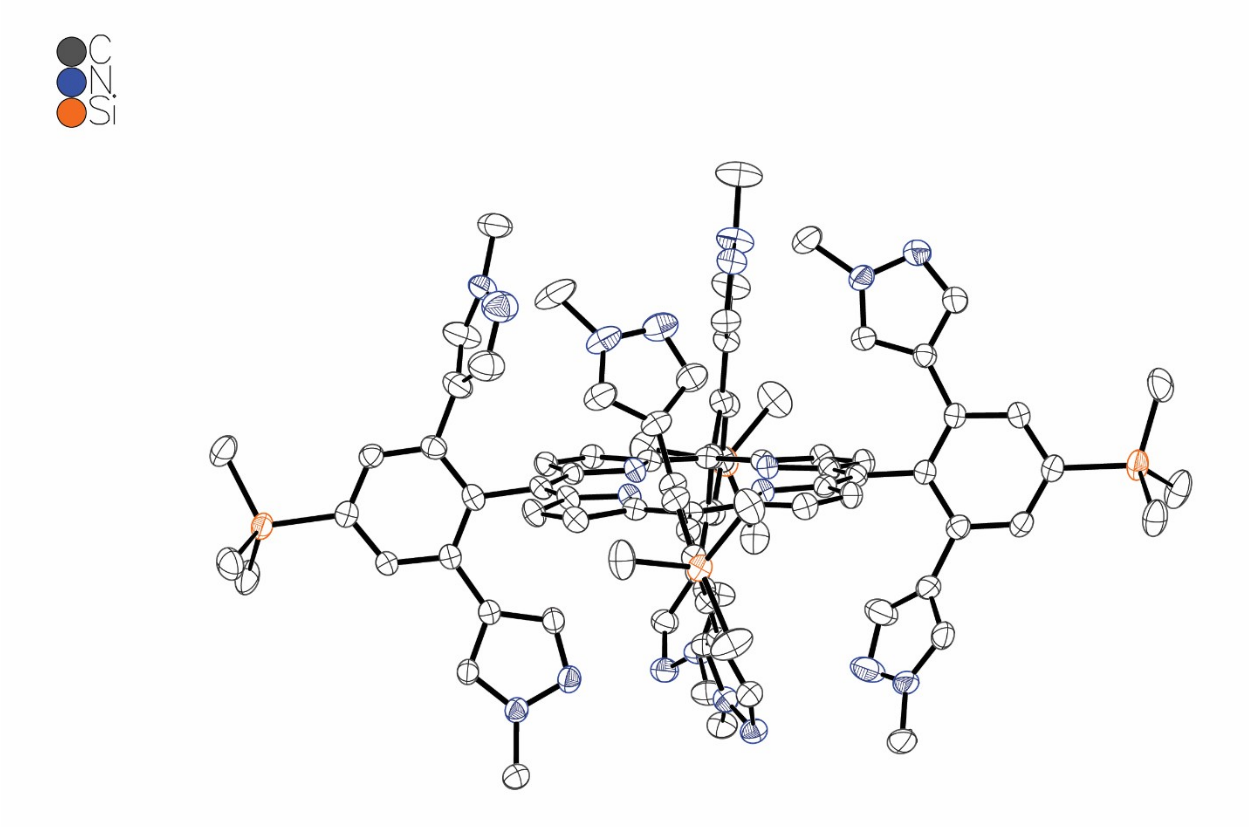

Figure S17. Thermal ellipsoid plot (50% ellipsoids) of the crystal structure of **2q**. H atoms, solvent, and minor components of the disorder omitted for clarity.

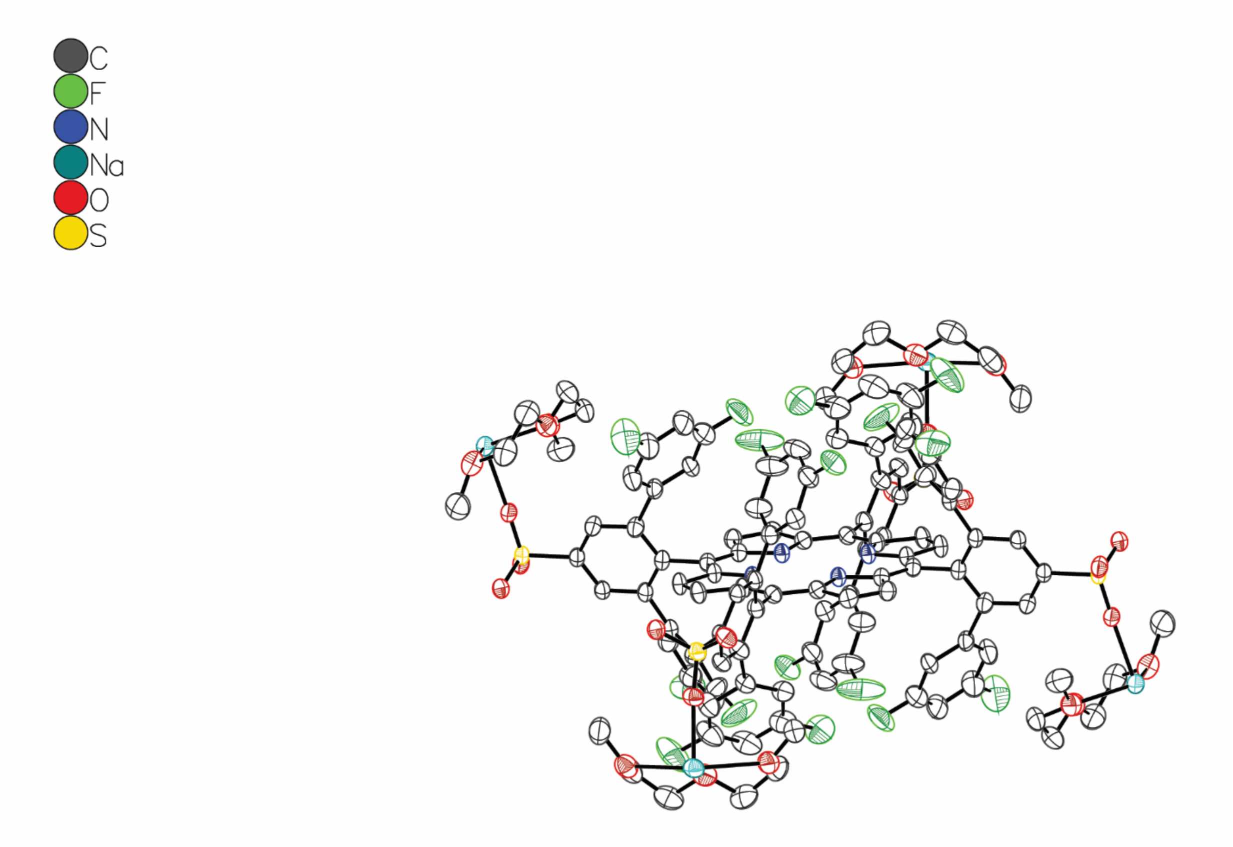

Figure S18. Thermal ellipsoid plot (50% ellipsoids) of the crystal structure of **3b**. H atoms, solvent, and minor components of the disorder omitted for clarity.

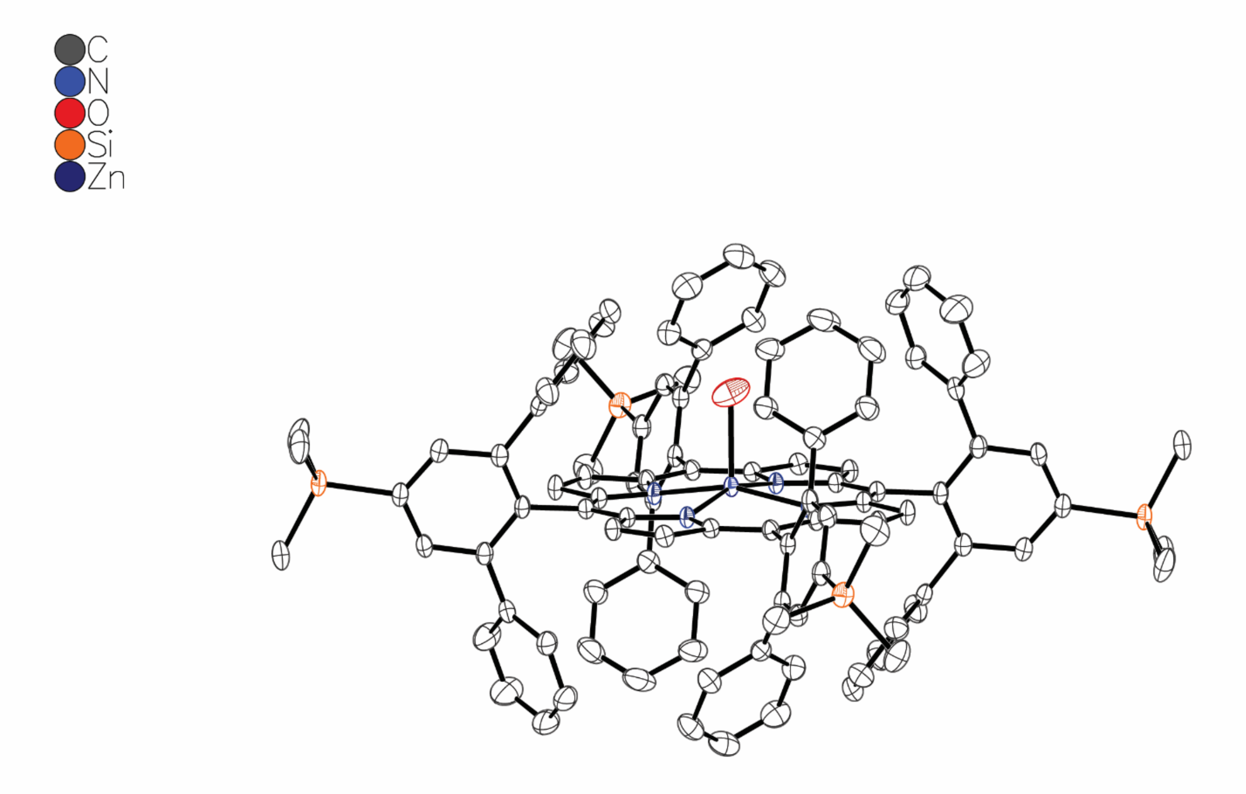

Figure S19. Thermal ellipsoid plot (50% ellipsoids) of the crystal structure of **4a**. H atoms, solvent, and minor components of the disorder omitted for clarity.

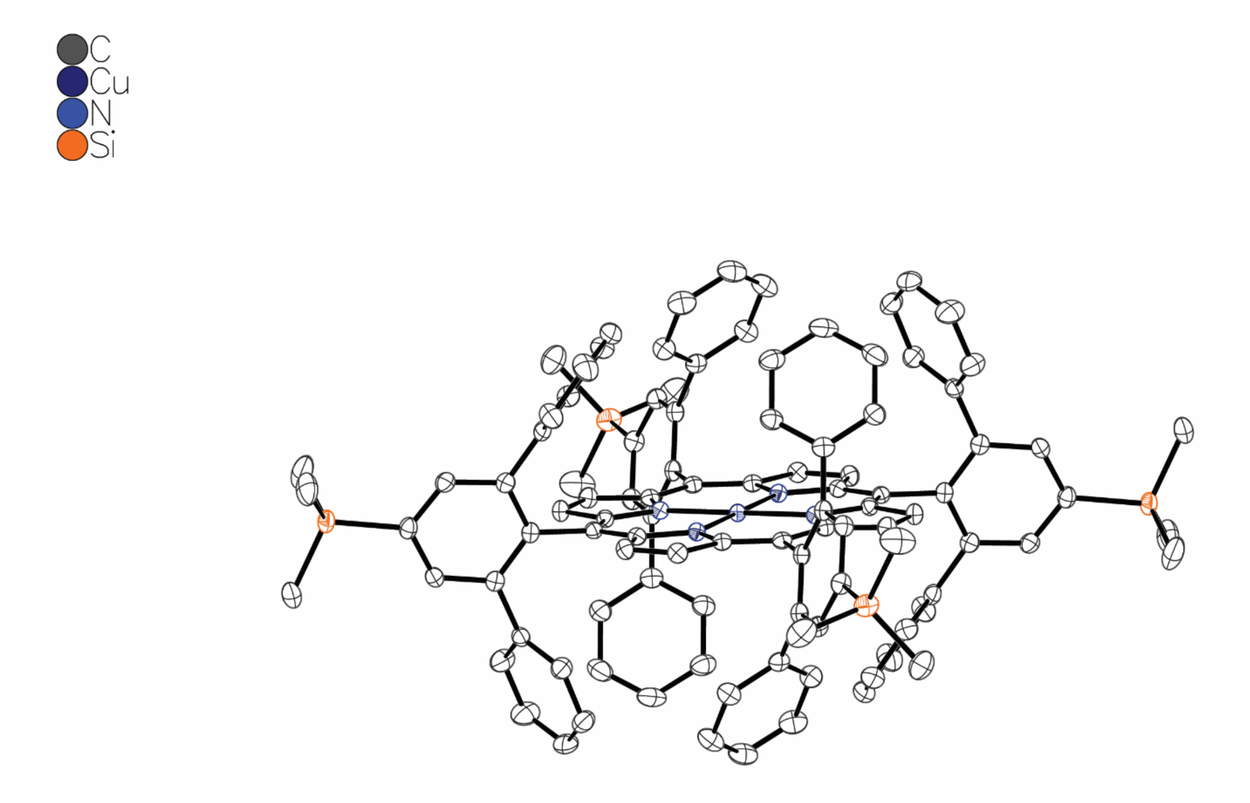

Figure S20. Thermal ellipsoid plot (50% ellipsoids) of the crystal structure of **4b**. H atoms and solvent omitted for clarity.

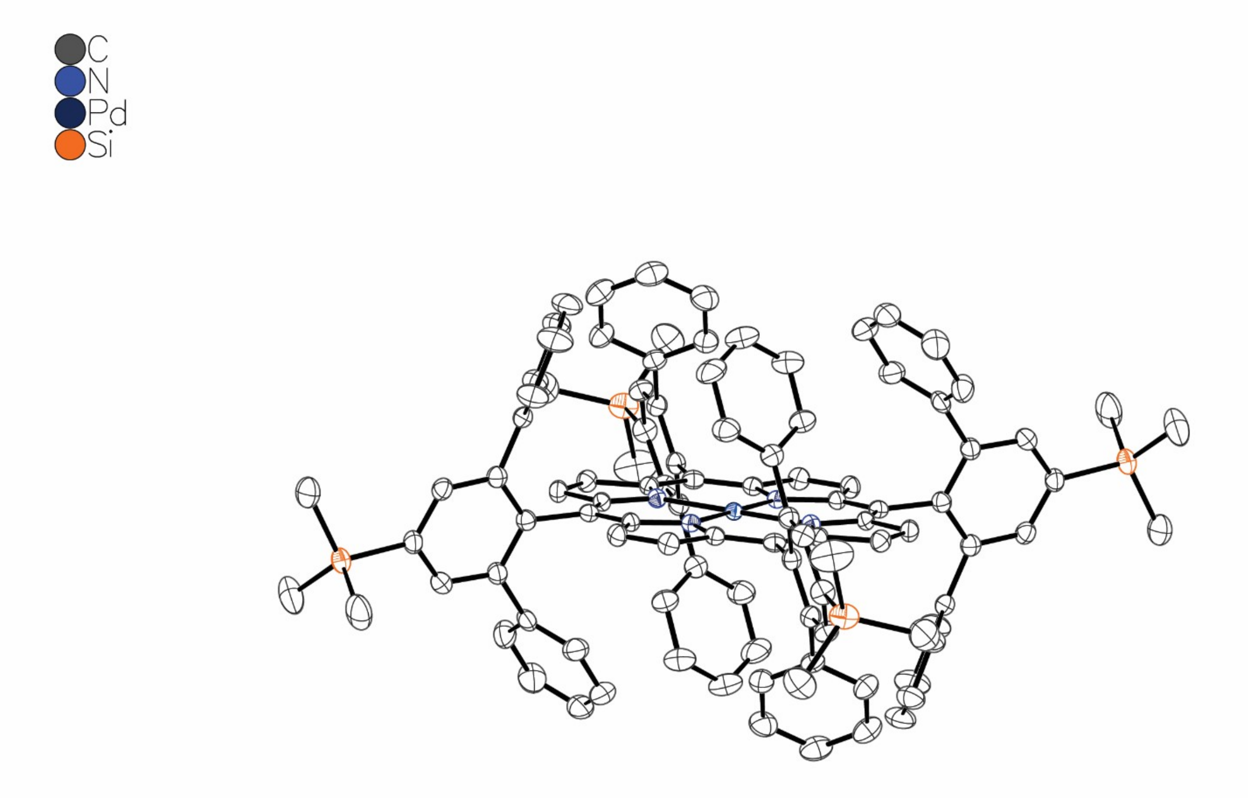

Figure S21. Thermal ellipsoid plot (50% ellipsoids) of the crystal structure of **4c**. H atoms and solvent omitted for clarity.

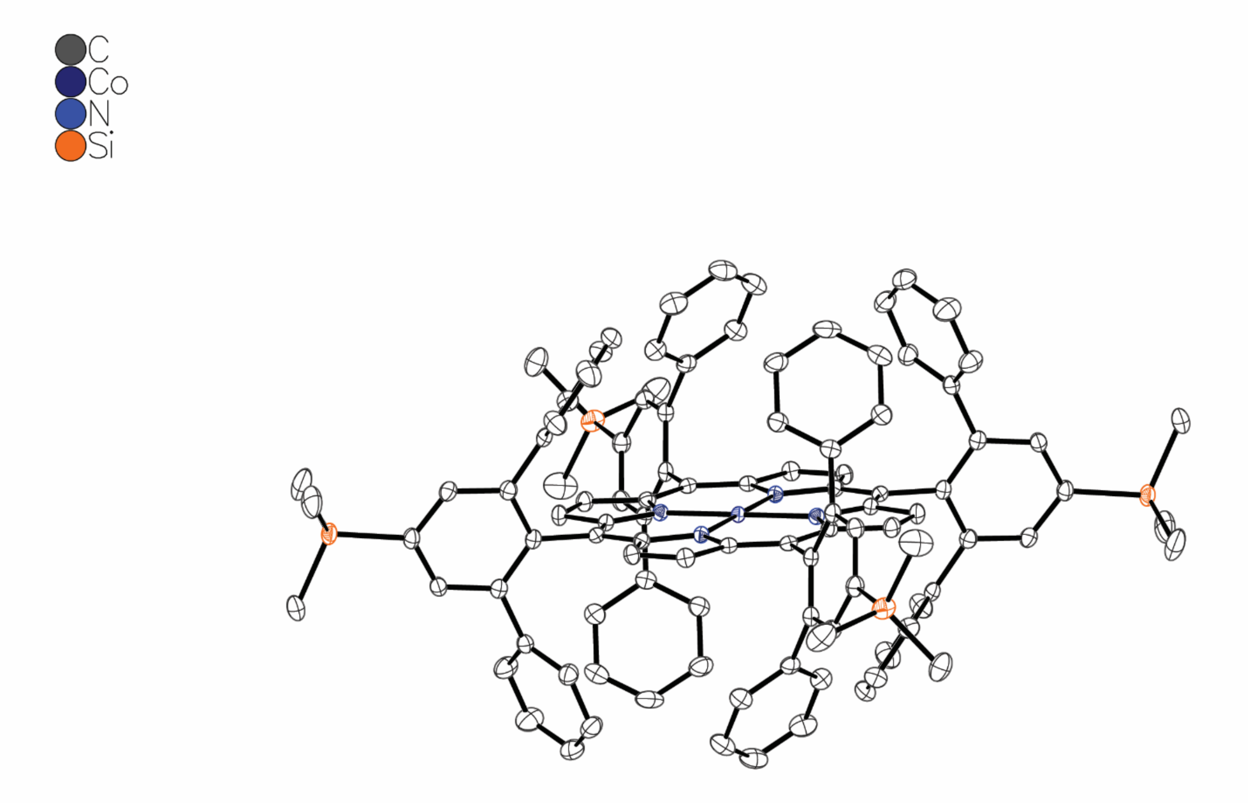

Figure S22. Thermal ellipsoid plot (50% ellipsoids) of the crystal structure of **4d**. H atoms and solvent omitted for clarity.

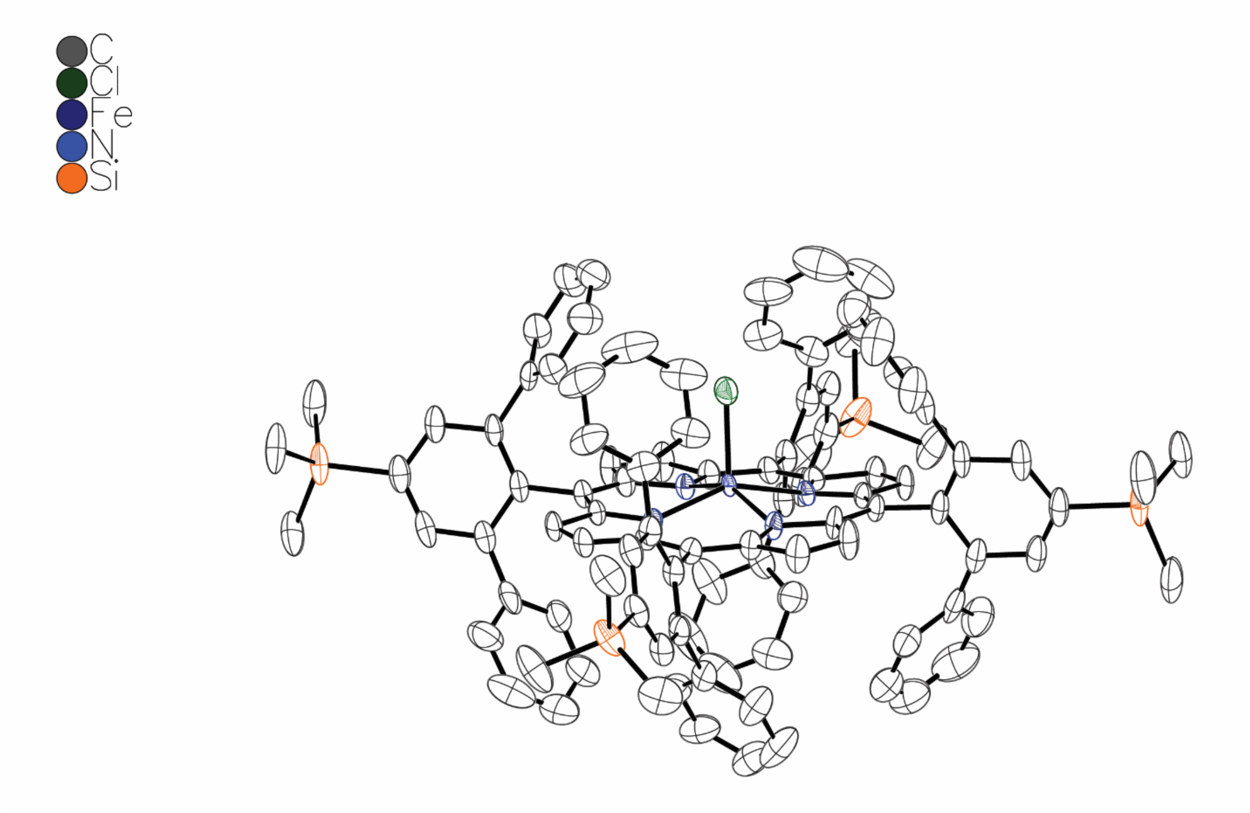

Figure S23. Thermal ellipsoid plot (50% ellipsoids) of the crystal structure of **4e**. H atoms, solvent, and minor components of the disorder omitted for clarity.

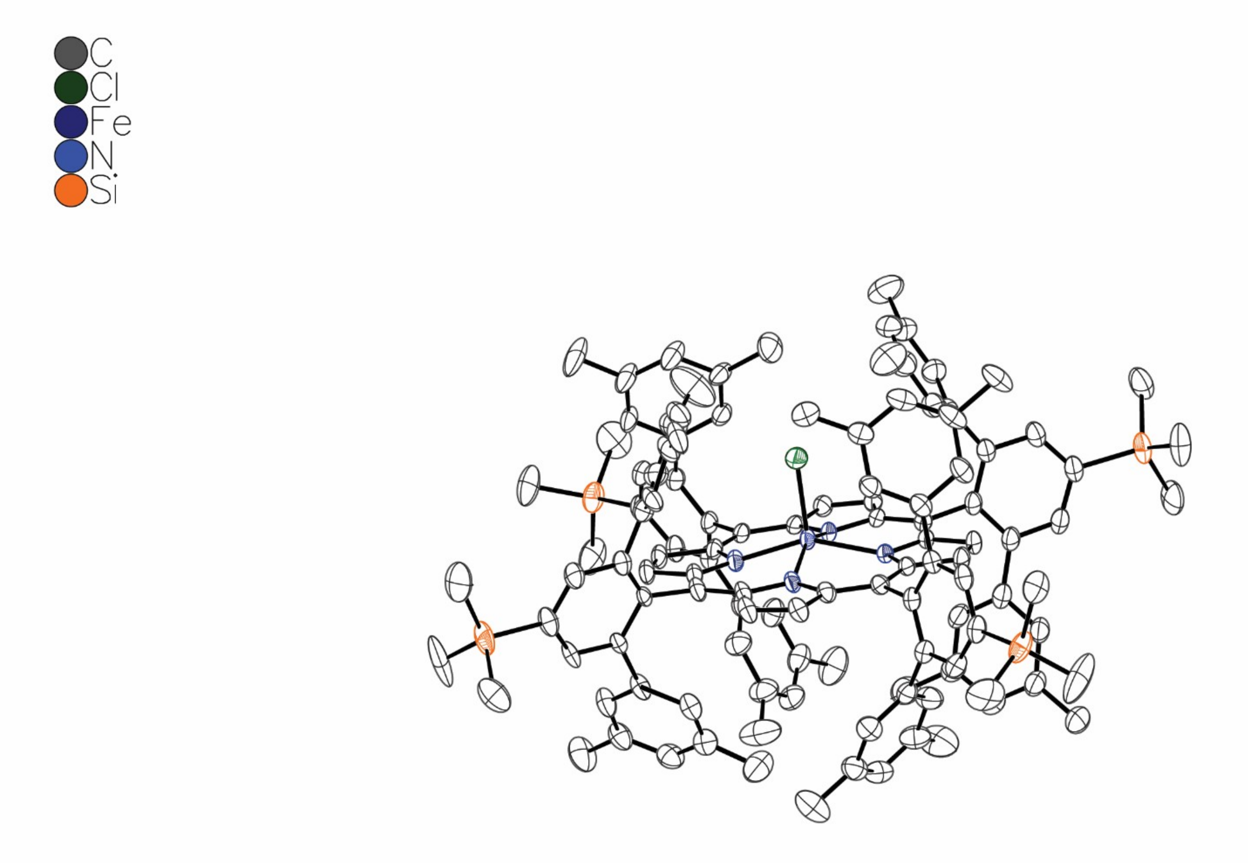

Figure S24. Thermal ellipsoid plot (50% ellipsoids) of the crystal structure of **4f**. H atoms, solvent, and minor components of the disorder omitted for clarity.

References

1. Durrant, J. D.; Votapka, L.; Sørensen, J.; Amaro, R. E., POVME 2.0: An Enhanced Tool for Determining Pocket Shape and Volume Characteristics. *J. Chem. Theory Comput.* **2014,** *10*, 5047-5056.

2. Pettersen, E. F.; Goddard, T. D.; Huang, C. C.; Meng, E. C.; Couch, G. S.; Croll, T. I.; Morris, J. H.; Ferrin, T. E., UCSF ChimeraX: Structure visualization for researchers, educators, and developers. *Protein Sci.* **2020,** *30*, 70-82.

3. Rigaku Oxford Diffraction *CrysAlis^{Pro} software system*, version 1.171.40.78a; Rigaku Corporation: Wroclaw, Poland, 2020.

4. Sheldrick, G. M., *SHELXT* – Integrated space-group and crystal-structure determination. *Acta Crystallogr. Sect. A* **2015,** *71*, 3-8.

5. Sheldrick, G. M., Crystal structure refinement with *SHELXL*. *Acta Crystallogr. Sect. C* **2015,** *71*, 3-8.

6. Dolomanov, O. V.; Bourhis, L. J.; Gildea, R. J.; Howard, J. A. K.; Puschmann, H., *OLEX2*: a complete structure solution, refinement and analysis program. *J. Appl. Crystallogr.* **2009,** *42*, 339-341.

7. Müller, P., Practical suggestions for better crystal structures. *Crystallogr. Rev.* **2009,** *15*, 57-83.
